# Supplementary material for: Study design, rationale and methods of the Revitalising Informal Settlements and their Environments (RISE) study: a cluster randomised controlled trial to evaluate environmental and human health impacts of a water-sensitive intervention in informal settlements in Indonesia and Fiji
Source: BMJ Open. 2021 Jan 8;11(1):e042850. doi: 10.1136/bmjopen-2020-042850 (PMC7798802; doi:10.1136/bmjopen-2020-042850)
Supplement: Author's manuscript [file bmjopen-2020-042850.draft_revisions.pdf]

BMJ Open is committed to open peer review. As part of this commitment we make the peer review history of every article we publish publicly available.

When an article is published we post the peer reviewers' comments and the authors' responses online. We also post the versions of the paper that were used during peer review. These are the versions that the peer review comments apply to.

The versions of the paper that follow are the versions that were submitted during the peer review process. They are not the versions of record or the final published versions. They should not be cited or distributed as the published version of this manuscript.

BMJ Open is an open access journal and the full, final, typeset and author-corrected version of record of the manuscript is available on our site with no access controls, subscription charges or pay-per-view fees (http://bmjopen.bmj.com).

If you have any questions on BMJ Open's open peer review process please email info.bmjopen@bmj.com

BMJ Open

# Study design, rationale and methods of the Revitalising Informal Settlements and their Environments (RISE) study: a cluster randomised controlled trial to evaluate environmental and human health impacts of a water sensitive intervention in informal settlements in Indonesia and Fiji



| | |
|---|---|
| Journal: | *BMJ Open* |
| Manuscript ID | bmjopen-2020-042850 |
| Article Type: | Protocol |
| Date Submitted by the Author: | 16-Jul-2020 |





| | |
|---|---|
| Complete List of Authors: | Leder, Karin; Monash University Faculty of Medicine Nursing and Health Sciences, School of Public Health and Preventive Medicine<br>Openshaw, John; Stanford University, Infectious diseases and Geographic Medicine Division<br>Allotey, P; United Nations University International Institute for Global Health<br>Ansariadi , Ansariadi; Hasanuddin University, Public Health Faculty<br>Barker, S Fiona; Monash University Faculty of Medicine Nursing and Health Sciences, School of Public Health and Preventive Medicine<br>Burge, Kerrie; Monash University, CRC for Water Sensitive Cities<br>Clasen, Thomas; Emory University, Department of Environmental Health Sciences, Rollins School of Public Health<br>Chown, Steven; Monash University, School of Biological Sciences<br>Duffy, Grant; Monash University, School of Biological Sciences<br>Faber, Peter; Monash University, School of Biological Sciences<br>Fleming, Genie; Monash University, School of Biological Sciences<br>Forbes, Andrew; Monash University Faculty of Medicine Nursing and Health Sciences, School of Public Health and Preventive Medicine<br>French, Matthew; Monash University, Monash Sustainable Development Institute<br>Greening, Chris; Monash University Faculty of Medicine Nursing and Health Sciences, Department of Microbiology<br>Henry, Rebekah; Monash University, Civil Engineering<br>Higginson, Ellen; University of Cambridge, Cambridge Institute for Therapeutic Immunology and Infectious Disease<br>Johnston, David W.; Monash University, Centre for Health Economics, Monash Business School<br>Lappan, Rachael; Monash University Faculty of Medicine Nursing and Health Sciences, Department of Microbiology<br>Lin, Audrie; University of California Berkeley, School of Public Health<br>Luby, Stephen; Stanford University, Infectious diseases and Geographic Medicine Division<br>McCarthy, David; Monash University, Civil Engineering<br>O'Toole, Joanne; Monash University Faculty of Medicine Nursing and Health Sciences, School of Public Health and Preventive Medicine |

| | |
|---|---|
| | Ramirez-Lovering, Diego; Monash University, Art, Design and Architecture<br>Reidpath, Daniel; Monash University - Malaysia Campus<br>Simpson, Julie; University of Melbourne School of Population and Global Health, Centre for Epidemiology and Biostatistics<br>Sinharoy, Sheela; Emory University, Rollins School of Public Health<br>Sweeney, Rohan; Monash University, Centre for Health Economics, Monash Business School<br>Taruc, Ruzka R; Hasanuddin University, Public Health Faculty<br>Tela, Autiko; Fiji National University College of Medicine Nursing and Health Sciences - Tamavua Campus, School of Public Health & Primary Care<br>Turagabeci, Amelia; Fiji National University College of Medicine Nursing and Health Sciences - Tamavua Campus, School of Public Health & Primary Care<br>Wardani, Jane; Monash University, Monash Sustainable Development Institute<br>Wong, Tony; Monash University, CRC for Water Sensitive Cities<br>Brown, Rebekah ; Monash University, Monash Sustainable Development Institute |


| | |
|---|---|
| Keywords: | PUBLIC HEALTH, Gastrointestinal infections < GASTROENTEROLOGY, Epidemiology < INFECTIOUS DISEASES, Public health < INFECTIOUS DISEASES, TROPICAL MEDICINE |
| | |


SCHOLARONE™ Manuscripts

*I, the Submitting Author has the right to grant and does grant on behalf of all authors of the Work (as defined in the below author licence), an exclusive licence and/or a non-exclusive licence for contributions from authors who are: i) UK Crown employees; ii) where BMJ has agreed a CC-BY licence shall apply, and/or iii) in accordance with the terms applicable for US Federal Government officers or employees acting as part of their official duties; on a worldwide, perpetual, irrevocable, royalty-free basis to BMJ Publishing Group Ltd ("BMJ") its licensees and where the relevant Journal is co-owned by BMJ to the co-owners of the Journal, to publish the Work in this journal and any other BMJ products and to exploit all rights, as set out in our licence.*

*The Submitting Author accepts and understands that any supply made under these terms is made by BMJ to the Submitting Author unless you are acting as an employee on behalf of your employer or a postgraduate student of an affiliated institution which is paying any applicable article publishing charge ("APC") for Open Access articles. Where the Submitting Author wishes to make the Work available on an Open Access basis (and intends to pay the relevant APC), the terms of reuse of such Open Access shall be governed by a Creative Commons licence – details of these licences and which Creative Commons licence will apply to this Work are set out in our licence referred to above.*

*Other than as permitted in any relevant BMJ Author's Self Archiving Policies, I confirm this Work has not been accepted for publication elsewhere, is not being considered for publication elsewhere and does not duplicate material already published. I confirm all authors consent to publication of this Work and authorise the granting of this licence.*

For peer review only

# Study design, rationale and methods of the Revitalising Informal Settlements and their Environments (RISE) study: a cluster randomised controlled trial to evaluate environmental and human health impacts of a water sensitive intervention in informal settlements in Indonesia and Fiji

Karin Leder[a], John J Openshaw[b], Pascale Allotey[c], Ansariadi Ansariadi [d], S Fiona Barker[a], Kerrie Burge[e], Thomas Clasen[f], Steven L Chown[g], Grant A Duffy[g], Peter A Faber[g], Genie Fleming[g], Andrew B Forbes[a], Matthew French[h], Chris Greening[i], Rebekah Henry[j], Ellen Higginson[k], David Johnston[l], Rachael Lappan[i] , Audrie Lin[m], Stephen P Luby[b], David McCarthy[j], Joanne E O'Toole[a], Diego Ramirez-Lovering[n], Daniel Reidpath[o], Julie A Simpson[p], Sheela S Sinharoy[f], Rohan Sweeney[l], Ruzka R Taruc[d], Autiko Tela[q], Amelia R Turagabeci[q], Jane Wardani[h], Tony Wong[e], Rebekah Brown[h], and the RISE Consortium

[a] School of Public Health and Preventive Medicine, Monash University, Australia
[b] Infectious diseases and Geographic Medicine Division, Stanford University, USA
[c] International Institute for Global Health, United Nations University, Malaysia
[d] Public Health Faculty, Hasanuddin University, Indonesia
[e] CRC for Water Sensitive Cities, Monash University, Australia
[f] Rollins School of Public Health, Emory University, USA
[g] School of Biological Sciences, Monash University, Australia
[h] Monash Sustainable Development Institute, Monash University, Australia
[i] Department of Microbiology, Monash University, Australia
[j] Civil Engineering, Monash University, Australia
[k] Cambridge Institute for Therapeutic Immunology and Infectious Disease, University of Cambridge
[l] Centre for Health Economics, Monash Business School, Monash University, Australia
[m] School of Public Health, University of California, Berkeley, USA
[n] Art, Design and Architecture, Monash University, Australia
[o] Monash University Malaysia, Bandar Sunway, Malaysia
[p] Centre for Epidemiology and Biostatistics, Melbourne School of Population and Global Health University of Melbourne, Australia
[q] School of Public Health & Primary Care, Fiji National University, Fiji

The RISE consortium: Fitriyanty Awaluddin [d], Becky Batagol[r], Lamiya Bata[j], Dieter Bulach[is], Bruce Cahan[t], Brett Davis[h], Mohammed El-Sioufi[n], Dusan Jovanovic [j], Michaela F Prescott[n], Emma Ramsay [g], Briony Rogers[h,u], Maghfira Saifuddaolah [d], Christelle Schang [j], Chi-Wen Tseng [j], Revoni Vamosi [q], Silivia Vilsoni [q], Isoa Vakarewa[v], Andi Zulkifli [d].

[r] Faculty of Law, Monash University, Australia
[s] Medicine, Dentistry and Health Sciences, Melbourne University, Australia
[t] School of Engineering Department of Management Science & Engineering, Stanford University, USA
[u] School of Social Sciences, Monash University, Australia
[v] Live and Learn, Fiji

Corresponding author: Prof K Leder, email: karin.leder@monash.edu

Key Words: RISE, health, environment, informal settlements, water sensitive, planetary health



For peer review only - http://bmjopen.bmj.com/site/about/guidelines.xhtml

## Abstract

**Introduction:** Increasing urban populations have led to the growth of informal settlements, with contaminated environments linked to poor human health through a range of inter-linked pathways. Here we describe the design and methods for the Revitalising Informal Settlements and their Environments (RISE) study, a transdisciplinary randomised trial evaluating impacts of an intervention to upgrade urban informal settlements in two Asia-Pacific countries.

**Methods and analysis**: RISE is a cluster randomised controlled trial among twelve settlements in Makassar, Indonesia and twelve in Suva, Fiji. Six settlements in each country have been randomised to receive the intervention at the outset; the remainder will serve as controls and be offered intervention delivery after trial completion. The intervention involves a water sensitive approach, delivering site-specific, modular, decentralised infrastructure primarily aimed at improving health by decreasing exposure to environmental faecal contamination. Consenting households within each informal settlement site have been enrolled, with longitudinal assessment to involve health and wellbeing surveys, and human and environmental sampling. Primary outcomes will be evaluated in children aged under 5 years and include prevalence and diversity of gastrointestinal pathogens, abundance and diversity of antimicrobial resistance (AMR) genes in gastrointestinal microorganisms, and markers of gastrointestinal inflammation. Diverse secondary outcomes include changes in microbial contamination; abundance and diversity of pathogens and AMR genes in environmental samples; impacts on ecological biodiversity and microclimates; mosquito vector abundance; anthropometric assessments, nutrition markers and systemic inflammation in children; caregiver- and self-reported health symptoms and healthcare utilisation; and measures of individual and community psychological, emotional and economic wellbeing. The study aims to provide proof-of-concept evidence to inform policies on upgrading of informal settlements to improve environments and human health and wellbeing.

**Ethics**: Study protocols have been approved by ethics boards at Monash University, Fiji National University and Hasanuddin University. Registration: ANZCTR (Trial ID: ACTRN12618000633280).




For peer review only - http://bmjopen.bmj.com/site/about/guidelines.xhtml

**Strengths and Limitations of this study**

- A transdisciplinary randomised controlled trial assessing a range of human and environmental health outcomes in urban informal settlements
- The trial design implicitly recognises that human health impacts are interlinked with environmental conditions
- The intervention involves a water sensitive approach in the delivery of site-specific, modular infrastructure to reduce faecal environmental contamination and improve water cycle management
- A participatory co-design process at household and community levels is intended to enhance acceptability, utilisation and upkeep of the intervention
- Primary health outcomes include objective markers of gastrointestinal health in children under five
- Prevalence, abundance and diversity of pathogens and antimicrobial resistance markers will be measured across both environmental and human faecal samples
- Health survey data collection will be complemented by a range of psychological, social, and economic outcomes, assessing impacts at individual and community levels
- A suite of ecological measurements to assess biodiversity, microclimates and vector abundance will be collected
- Follow-up will be maintained for 2 years following completion of the intervention
- Logistic constraints for communities to be involved in an RCT, including population size, limit external validity of findings, although working in two countries, Indonesia and Fiji, improves generalisability
- The RCT design enables settlement-level cluster randomisation, but blinding to the intervention is impossible
- The intervention focuses on reduction of human faecal contamination of the environment and only incidentally addresses other pathways of exposure to faecal contamination



For peer review only

For peer review only - http://bmjopen.bmj.com/site/about/guidelines.xhtml

## INTRODUCTION

Informal settlements are home to more than a billion people, mostly in rapidly growing urban areas of low- and middle-income countries (LMICs). With predictions suggesting that up to three billion people could be living in urban informal settlements by 2050[1], it is imperative to address vulnerabilities faced by informal settlement communities. Informal settlements are often located in inhospitable parts of cities prone to flooding, residents frequently face insecure land tenure, and communities often have inadequate provision of essential centralised urban services including clean water and appropriate sanitation facilities[2]. The confluence of factors faced by residents of informal settlements living in poor conditions results in exposure to compromised ecological conditions and environmental contamination with pathogens and disease vectors, which leads to deleterious impacts on health and wellbeing[3].

The Sustainable Development Goals (SDGs), a set of aspirational goals and targets for attaining sustainable development by 2030, envision that urban communities will become more inclusive, safe, resilient, and sustainable[4]. Reducing environmental exposure to infectious agents transmitted in human faeces is specifically addressed in the SDGs (SDG 6.1 and 6.2); given the impact of such exposures on the burden of disease, they are also implied as a priority by the health goals. However, conventional engineering solutions for water and sanitation—reticulated water and sewerage systems--often do not reach informal urban settlements[2 5 6], mainly as a result of the economic poverty, legal land tenure issues, and lack of political power among residents in these communities. Low-cost solutions, such as household water treatment and on-site sanitation, have had mixed results in recent trials[7-10]. Interventions attempting to address the adverse conditions must therefore consider the complex exposure pathways that link physical aspects of the environment to human health outcomes, and accordingly must interrupt multi-faceted vulnerabilities.

The Revitalising Informal Settlements and their Environments (RISE) trial will implement an intervention designed to address these challenges. The RISE study (www.rise-program.org)[11] will collaborate with communities to design and implement a decentralised wastewater infrastructure which integrates sustainable, water sensitive technology into buildings and landscapes. The infrastructure will be implemented at dwelling, neighborhood, and precinct scales. The focus of the RISE water sensitive intervention is to reduce direct contact with faecal contaminants among informal settlement residents. Delivery of conventional water and sanitation services, namely toilets and hand basins, is incorporated into the RISE study, but in contrast to traditional water, sanitation and hygiene (WASH) approaches, the intervention extends beyond promotion of improved sanitation, drinking water supplies, and handwashing practices[7-10]. Instead, the RISE study will also include settlement-scale infrastructure delivery to address environmental contamination from inadequate attention to management of faecal waste and poorly maintained septic systems, both of which are likely to contribute to suboptimal health impacts in high-density living settings such as occurs in urban informal settlements. The RISE study will also concurrently address upgrading of physical access within communities to limit residents' exposure to environmental pollutants, improve overall water cycle management, diversify water source supplies, and attend to water drainage and flood management. This holistic, settlement-scale approach addresses the recent call for "transformative WASH" or "WASH-plus" solutions[12 13].

Here we describe the rationale of the RISE intervention and the study design and methods of a cluster randomized controlled trial (RCT) to assess the impact of the intervention on the environment and human health and wellbeing. The cluster RCT is being conducted in Makassar, Indonesia and Suva, Fiji in cooperation with local and national governments.

## HYPOTHESES AND AIMS

The hypothesised sequence of change in the RISE study is that implementing a water sensitive intervention in informal settlements leads to improved physical environments, which in turn results in



For peer review only - http://bmjopen.bmj.com/site/about/guidelines.xhtml

reduced human exposure to pathogenic faecal contamination and flooding hazards, resulting in improved human and ecological health and wellbeing (Figure 1). Specifically, we hypothesise that the intervention will reduce environmental contamination with human faeces. We further hypothesise that this will reduce residents' exposure to gastrointestinal pathogens that cause diarrhoeal disease, poor gastrointestinal function, and nutritional deficits, which particularly impact on young children living in informal settlement conditions, as well as reducing exposure to facecal sources of antimicrobial resistance (AMR) genes. Our hypotheses align with the planetary health approach[14], acknowledging the complex interplay between multiple factors which include health, environmental conditions, urbanisation, water and sanitation management, gender and socio-economic equity, and climate change.

We have three central scientific questions and aims that align with our hypotheses.[1]

***Aim 1: Environment***

We will first assess whether the RISE intervention leads to improved environmental characteristics. Urbanisation alters physical environmental conditions such as air temperature, soil contamination, and water quality, and causes transformations in local biodiversity and changes in ecological communities and their functioning[15-18]. Cities tend to have warmer environments than surrounding areas due to the urban heat island effect[19 20], and in tropical informal settlements, high housing density and inadequate formal solid, greywater and faecal waste processing pathways result in considerable eutrophication and faecal contamination of soil and water[21]. Increased populations of mammal (e.g. rats) and arthropod (e.g. mosquito) vectors are often present[22-24], likely due to a combination of thermal changes, habitat modification, resource concentration, and high human population densities[25-27].

Our aim is to assess the impact of delivery of the water sensitive intervention on the physical, thermal, hydrological and microbiological environments. Changes are expected to make residents less vulnerable to the impacts of flooding, reduce the abundance of vectors, increase the diversity of urban ecological communities and alter the composition of microbial communities. Additionally, by improving human excreta management strategies, we aim to decrease faecal contamination of the environment, thereby reducing exposure to pathogens and AMR genes in water and soil.

***Aim 2: Human health***

Our second aim is to assess whether the intervention leads to improved physical health outcomes as a result of improved environmental conditions, as hypothesized above. Prevailing bio-physical conditions in informal settlements are conducive to the spread of infectious diseases. Ample vector breeding sites and poor preventative practices lead to mosquito-borne diseases[28 29]. Children suffer from diarrhoea and poor nutrition [30 31], with contaminated water and soil acting as a source for transmission of pathogens derived from animal, human, and environmental origins[32 33]. Antimicrobial-resistant infections are also a growing threat to human health globally[34], and inadequate faecal waste management leads to environmental contamination which may promote transmission of multi-drug resistant pathogens between humans and the environment[35 36]. Young children under the age of five living in these suboptimal conditions are at especially high risk for under-nutrition, diarrhoea, and poor growth and development. Further worsening their physical health is the high prevalence of environmental enteric dysfunction, a chronic inflammatory condition of the intestine which is associated with poor absorption of nutrients, growth faltering, and impaired response to live enteric vaccines[37 38].

By designing and delivering decentralized infrastructure appropriate to and scalable within informal settlements, we aim to improve faecal sludge handling and reduce contact with faecal contamination in

---

[1] In addition to these three scientific questions, the RISE program includes two other research objectives: (1) to develop novel practices for built environment and community co-design, and (2) to develop the financial and policy frameworks for scaling up the intervention.



For peer review only - http://bmjopen.bmj.com/site/about/guidelines.xhtml

order to interrupt the connection between the human faecal stream and the environment. These impacts are expected to decrease children's exposure to gastrointestinal pathogens, as detected by molecular methods in faecal samples, to decrease environmental enteric dysfunction, and to decrease the abundance and diversity of AMR markers detected in children's gastrointestinal tract. Flood mitigation and improved access within communities will further decrease human contact with the overall reservoir of environmental pathogens, and falling vector abundance may decrease mosquito-borne disease.

***Aim 3: Wellbeing***

Our third aim is to determine whether the intervention affects the physical environment in ways which impact on community wellbeing and individual subjective psychological and economic wellbeing. Changes to the physical environment affect inhabitants' lifestyle, mental health, self-image, perceptions of safety, and social cohesion[39-41]. They also impact on water security, local economic activities, financial wellbeing, time allocation for water collection and time available for paid work[42-46].

We aim to change the physical environment to positively impact inhabitants' evaluative and hedonic subjective wellbeing; change how people allocate their time; and improve economic activity by increasing employment opportunities and time spent on income-generating activities. At a community level, we aim to improve residents' perceptions of social cohesion and, motivated through the local co-design approach during intervention development, improve community resilience by enhancing collective efficacy.

***Additional outcomes***

In addition to these three key scientific aims, we will evaluate the evidence on the feasibility and impact of the approach to explore the potential for scaling up water sensitive interventions throughout the Asia-Pacific region through rigorous generation of evidence. We will engage with governments, investors and implementers to determine priorities and economic drivers, and identify barriers to intervention to address the environmental challenges of urban informal settlements.

## STUDY METHODS AND ANALYSIS

### Overview of the study design and timeline

To evaluate the RISE intervention, we are conducting a cluster RCT involving 12 informal settlements in Suva, Fiji, and 12 in Makassar, Indonesia, with clustering at the settlement level. In each country, half of the sites have been randomised as intervention communities and will receive an early upgrade, with the remaining serving as controls. Monitoring and assessment will be conducted in all settlements over an approximate five-year timeline which will include baseline data collection, the design and build implementation phase of the project, and 2 years of post-build monitoring (Figure 2). After completion of the study, the intervention will be implemented in the 12 control settlements.

### Study settings

The study is being conducted across two countries where large portions of the population are subject to inadequate water and sanitation infrastructure. Indonesia and Fiji were purposively selected to represent a diversity of characteristics across the Asia-Pacific which might influence intervention effects, including climatic factors, water security, tidal inundations, population density, sanitation practices, and socioeconomic and cultural conditions. Within each country, candidate sites were selected in consultation with local government authorities, intervention funders, research partners, communities, and other organisational stakeholders. Formative work consisted of four phases: a) extensive reconnaissance visits to both cities to understand the range of site conditions and to scope potentially suitable sites, b) early engagement with local and national governments to determine priorities and preferences, and to ascertain tenure status for each site, c) household enumeration surveys to determine approximate



For peer review only - http://bmjopen.bmj.com/site/about/guidelines.xhtml

population size and demographics in each settlement, and d) extensive discussions with community leaders and residents to determine their willingness to participate and priorities.

Makassar, the provincial capital of South Sulawesi, has seen rapid urbanisation and population growth in the last decade, with unplanned city growth resulting in a dramatic rise of informal settlements. The 12 settlements of the RISE project are dispersed across the city and represent a diversity of settlement typologies. While the settlements are located in various flood plains and coastal areas, they are all characterised by dense and precariously built housing, little open space, poor vehicular and pedestrian access, and water stressors. Water bodies and soil are typically contaminated with solid waste derived from animal and human sources, and with domestic black and grey wastewater. Seasonal and other flooding is common, requiring makeshift planking for access. Despite some variation in socio-economic status within and across settlements, sites typically house the most economically vulnerable populations. Households across the settlements predominantly live on small parcels of privately-owned land often with one house, or in some instances two or three houses occupied by extended family groups, with varying degrees of land tenure security.

Similar to the settlement sites in Indonesia, the 12 RISE informal settlements in Suva, Fiji, reflect a range of typologies that exist in Fiji and the broader Pacific context. While the urban morphology varies between settlements, they are typically high-to-medium density clusters with some open space and, in some instances, lush vegetation. Some settlements are prone to significant pluvial and fluvial flooding; housing is of varied quality and construction type but is typically characterised by lightweight structures raised off the ground. Tenure status is characteristic of informal settlements in Fiji and the broader Pacific region with limited private land ownership in the selected communities, instead relying on community leases on state land or informal agreements with traditional land-owning units on native lands.

**Settlement eligibility and selection criteria**

Data gathered on potential sites characterised local water supplies, sanitation systems, and flooding events and risk. Inspections were conducted at potential sites to confirm feasibility and accessibility. Site locations and sizes were informed by the UN-Habitat informal settlement selection approach[47]. Final selection criteria consisted of settlement size, environmental, and construction related factors (**Table 1**).

| **Table 1: Selection criteria for informal settlements** | |
|---|---|
| Settlement size, location, and demographics | 1. 30-100 houses, ideally all in one consolidated group<br>2. Physically separated from other settlements with clear physical boundaries<br>3. No conditions calling for future permanent relocation (i.e. landslide risk)<br>4. Representing the most vulnerable populations of the city<br>5. Location in vulnerable areas with presumed high risk of water-borne disease<br>6. Presence of at least 5-10 children under 5 years |
| Land tenure | 7. Occupants with secure tenure (ownership of the house and land that it sits on, or permission to occupy) |
| Environmental conditions | 8. Presence of water stressors including flooding, poor drainage, limited sanitation and water supply |
| Construction related | 9. Potential for replication and scaling of designs |
| Participation | 10. Settlement leaders and inhabitants consenting for health and environmental assessments and infrastructure modification |



For peer review only - http://bmjopen.bmj.com/site/about/guidelines.xhtml

**Site and household recruitment**

Community recruitment occurred after extensive discussion with stakeholders, government, community leaders and householders, and was performed by trained local community field worker teams. The unit of recruitment for assessment of the intervention is at the household level. We have attempted to recruit and enroll all households within the agreed boundaries of each informal settlement, with written informed consent obtained from heads of households. Householders were able to consent to participation in the overall RISE project with or without consenting to additional individual survey and sampling components.

**Description of the intervention**

The water sensitive approach to upgrading implemented in the RISE study integrates urban design and urban water cycle planning and management, focusing on water conservation and reducing environmental pollution by improving the quality of wastewater and stormwater prior to reuse or discharge to the environment. Implementation of water sensitive infrastructure will be site-specific, co-designed with each community, and adapted to the different biophysical and sociocultural conditions in each informal settlement. The intervention includes specific components collectively designed to reduce residents' exposure to faecal contamination, requiring evaluation of critical faecal contamination and community exposure pathways at each site to determine optimal available technology solutions. Delivery of traditional approaches to water and sanitation services such as private toilets and hand basins are incorporated. Importantly, solutions also include installation of broader decentralised water infrastructure options to control and reduce the release of contaminated sewage and household greywater into the environment, improve flood mitigation, lower exposure to polluted environments within settlements through better access options, and provide source water availability through rainwater harvesting and wastewater recycling.

Specific infrastructure options include (Figure 3):

- New integrated 'wetpods': these are comprised of a toilet, hand basin and rainwater tank;
- Toilet connections: pressure sewers and traditional collection and treatment systems (pipes, manholes, septic tanks and drains);
- Wastewater treatment: constructed wastewater treatment wetlands for black water[48-50], and biofilters and biofilter drains for grey water[51-54];
- Stormwater drainage and treatment: this includes resizing, reprofiling and formalising drains, and swales, raingardens and constructed stormwater treatment wetlands and permeable paving[55-58];
- Water supply security: rainwater tanks and collection, connection to municipal water supply, water supply disinfection and protection of existing shallow well water supplies;
- Access roads, utility corridors, raised pathways;
- Flood management: including backflow prevention, minor localised spot-filling, minor terrain modification and flood protection walls.

The water sensitive approach has been shown to successfully address urban water management challenges in an integrated way in high income economies[59-66], and will be adapted for informal settlements in the RISE study. We will initially design and deliver a pilot project in each country to test key water sensitive technologies and evaluate soils and plants best suited to local conditions. Monitoring system performance from the two pilot communities will underpin adaptive design adjustments to full-scale implementation.



For peer review only - http://bmjopen.bmj.com/site/about/guidelines.xhtml

As a socio-technical intervention, infrastructure design and delivery will be adapted to context-specific social and bio-physical characteristics and constraints of each community through a participatory process of co-design. The co-design process will promote engagement and informed decision-making with residents of each settlement, particularly emphasising participation by women, children, elderly, disabled and other groups that may have specific needs. The process is characterised by active community participation to ensure participants' preferences for solutions are heard, including informed choices regarding the physical location of new infrastructure. It brings the international community of researchers and practitioners together with the residents living in the study communities, thereby building understanding and a sense of value of the infrastructure to increase the likelihood of success of long-term operations and maintenance. Communal infrastructure will be delivered by local contractors supervised by the project team. Long-term operation and maintenance responsibilities will be addressed via training and ongoing engagement of community representatives and local authorities. By utilising local construction expertise and engaging and empowering stakeholders at household, community, and city levels, we aim to build knowledge and trust with local communities, institutions, and governments to ensure the infrastructure is built and maintained for sustainable, long term benefit.

**Data collection**

Data collection is conducted by field workers specifically employed for assessment of trial outcomes; this team is separate from the personnel implementing the intervention. Data collection comprises biospecimen sampling at both the settlement and household levels, field data measurements, and household surveys. Specimen collection involves blood and faeces from children under the age of 5 years; environmental soil, water and animal scat samples; and mosquito and rodent trapping, with intended sampling frequency as shown in Table 2. Ecological field data assessment involves continuous passive acoustic monitoring, temperature logging, and flood assessment including the use of satellite imagery. Anthropometric measures are taken annually among children under aged 5 years. Surveys are administered quarterly, with different modules asked of varying-aged participants to assess physical health, healthcare utilisation, psychological wellbeing, financial wellbeing, time-use, and perceptions of social cohesion and collective efficacy. Questions regarding household level data (i.e. household member composition, flooding) are administered preferentially to the adult female head of household and questions regarding children under the age of 15 are preferentially directed to the adult caregiver who has seen them most days in the past 7 days. Questions regarding adult health and wellbeing, financial wellbeing, time-use, and perceptions of social cohesion and collective efficacy are preferentially administered to: (i) primary caregivers where households contain children under 15 years, and (ii) female or (iii) male heads for households with no children. If the preferred respondent is not at home, field teams attempt two re-visits. To maintain participation over the trial period small culturally appropriate gifts will be provided.

**Table 2: Data collection tools and specimens for primary and secondary outcomes**

| | Minimum Frequency* | | | |
|---|---|---|---|---|
| **Primary outcomes** | C | Q | B | A |
| Faeces collection, children < 5 years of age for:<br>• Prevalence, diversity and concentration of bacterial, viral, protozoan, and helminth gastrointestinal pathogens in children < 5 years of age (composite primary outcome)<br>• Concentration of intestinal inflammation markers representing environmental enteropathy in children < 5 years of age | | | X | |



For peer review only - http://bmjopen.bmj.com/site/about/guidelines.xhtml

| | | | | |
|---|---|---|---|---|
| • Diversity and abundance of AMR genes in gastrointestinal microorganisms of children < 5 years of age (composite primary outcome) | | | | |
| **Secondary outcomes** | | | | |
| ***Physical Health Measures*** | | | | |
| Blood collection for haemoglobin and blood inflammatory markers, children < 5 years | | | | X |
| Anthropometry measures (length-for-age, weight-for-age and weight-for-length Z-scores) measure, children < 5 years of age | | | | X |
| ***Surveys: Household and community demographic, health and wellbeing*** | | | | |
| Household members and demographics | | | | X |
| Household member behaviors and exposures | | | X | |
| Access to sanitation, water, and hygiene facilities^ | | | | ^ |
| Caregiver-reported acute symptoms (diarrhoea, respiratory infections and febrile illness), children < 5 years of age | | X | | |
| Healthcare system utilisation, children < 5 years of age | | X | | |
| Self-reported or caregiver-reported acute symptoms, healthcare system utilisation, and subjective general health (5 point rating scale) among adults and children 5-15 years | | | X | |
| Caregiver assessed quality of life (Pediatric Quality of Life Inventory, PedsQL, emotional functioning dimension), children 5-15 years | | | X | |
| Self-reported life satisfaction – multiple domains (10 point rating scale), adults | | | X | |
| Center for Epidemiologic Studies Depression Scale [CES-D-10], adults | | | | X |
| Self-reported major adverse life events, adults | | | X | |
| Time-use surveys, children 5 to 15 years and adults^ | | | | ^ |
| Housing tenure+ and collective efficacy (Likert 5 point scales) | | | | X |
| Indicators of household assets and expenditure^ | | | | ^ |
| Intermittent qualitative surveys and focus groups | NA | | | |
| ***Measures of environmental faecal contamination*** | | | | |
| Soil and water (potable, recreational, wastewater) samples and animal scat samples for chemical and microbiological analyses (including markers of faecal contamination; prevalence, diversity and concentration of pathogens; abundance and diversity of AMR genes) | | X | | |
| ***Ecological measures and biodiversity*** | | | | |
| Indoor and outdoor thermal data logging | X | | | |
| Passive acoustic monitoring | X | | | |
| Aerial and satellite imagery | | | | ^ |
| Flood and rain gauges+ | | | | X |
| Mosquito trapping | | X | | |
| Rodent trapping+ | | X | | |
| *C = continuous; Q = quarterly; B = Biannual; A = Annual ^First and final years of study +Indonesia only | | | | |

## Public involvement

Members of enrolled communities are involved in the design and conduct of this trial, including the design and implementation of the intervention as described above.

## OUTCOMES

### *Primary outcomes*



For peer review only - http://bmjopen.bmj.com/site/about/guidelines.xhtml

We define our first primary outcome as the prevalence and diversity of bacterial, viral, protozoan, and helminthic gastrointestinal pathogens in children under 5 years. Additional primary outcomes are i) the abundance and diversity of AMR genes in gastrointestinal microorganisms of these children; and ii) the concentration of intestinal inflammation and permeability biomarkers in stool representing environmental enteric dysfunction.

We will use various molecular approaches to assess the presence and concentration of pathogens, microbial communities, and AMR genes in human faecal samples. As indicated below, the same methodologies will be applied for processing of environmental samples, thereby optimising assessment of the impact of the intervention on both environmental and human health outcomes. Total nucleic acids will be extracted from faecal samples in-country. Pathogen levels in samples will be measured by quantitative PCR (qPCR) using TaqMan Array Cards [67 68] targeting multiple bacterial, viral, protozoan and helminthic pathogens. rRNA gene amplicon sequencing will measure microbial biodiversity. Shotgun metagenomic sequencing will be used to gain information about the functional capabilities of pathogens in each sample, including the classification and enumeration of AMR genes. Expected environmental enteric dysfunction serological markers include faecal myeloperoxidase[69], faecal α-1-antitrypsin[70], and faecal neopterin[71].

***Secondary outcomes***

*Human health secondary outcomes*

Secondary health outcomes include caregiver- and self-reported acute symptoms, healthcare utilisation, inflammatory markers in blood, haemoglobin levels, and anthropometry measures. Caregiver- and self-reported symptoms will be collected via quarterly standardised questionnaires throughout the trial, including the presence of diarrhoea, respiratory and undifferentiated febrile illness over the 7 days prior to the survey visit[72]. Symptoms will be assessed quarterly in children under the age of 5 and every 6 months in children between 5-15 years of age and adults. In all cases, diarrhoea is defined as ≥ 3 loose or watery stools in a 24-hour period. Healthcare utilisation (visitation to healthcare provider, hospitalisation, antibiotic use) by children under 5 years of age within the three months prior to the visit will be surveyed quarterly; for children 5 -15 years of age and adults surveys will be 6-monthly.

Blood from children under 5 years of age will be taken annually by venipuncture. Haemoglobin levels will be measured in the field using a point-of-care device. Plasma and whole blood will be stored for further analyses which will include markers of nutrition and inflammation. To obtain length-for-age, weight-for-age and weight-for-length z-scores in children under 5 years of age, trained staff will follow standard procedures for anthropometric measurements[73]. Pairs of trained anthropometrists will measure standing (in children between 2 and 5 years of age) or recumbent (less than 2 years of age) length (accurate to 0.1 cm) and weight without clothing (accurate to 0.1 kg) in triplicate. In both cases, the median of the three measurements will be used in the analysis[74].

*Community and individual wellbeing secondary outcomes*

Secondary outcomes related to individual and community wellbeing include self-reported general and emotional wellbeing, time-use, measures of collective efficacy and water security, and indicators of socioeconomic status. We will assess the subjective general health and emotional wellbeing of children aged 5-15 years using the (parent-proxy) Paediatric Quality of Life InventoryTM 4.0 Emotional Functioning Scale (PedsQL)[75]. Subjective general health, life satisfaction and major life events (i.e., family death, serious illness, marriage break-up, bankruptcy, victim of crime) of adults will also be collected 6-monthly[76 77]. Child (age 5-15 years) and adult time-use (e.g. minutes/hours spent on different activities in past week) will also be collected. We will assess collective efficacy to monitor changes in community wellbeing, trust, and feelings of safety[78]. Indicators of socioeconomic status (i.e.,



For peer review only - http://bmjopen.bmj.com/site/about/guidelines.xhtml

education levels, house building materials, assets owned, bank account, primary activity, time spent working and subjective financial wellbeing) will also be collected to facilitate distributional analyses of any observed impacts. We will also estimate the costs of the intervention and report on cost-effectiveness and costs-benefits.

*Ecological and environmental secondary outcomes*

Secondary outcomes for environmental effects include quarterly chemical and microbiological measures to assess faecal contamination of both soil and water (municipal supply, recreational and wastewater), quarterly measures of vector abundance, and continuous measures of the thermal environment and overall biodiversity. We will compare faecal contamination in water and soil samples across intervention and control settlements. Sample collection and transport will be performed using previously described and optimised methods[79 80] and processed for faecal bacteria quantification[81]. Nucleic acid extraction will be performed in-country via methods that enable simultaneous detection of viruses, bacteria, protozoa, and helminths from environmental samples, as confirmed through optimisation experiments already performed. As with human faeces, pathogen presence and concentrations in samples will be measured using TaqMan Array Cards ([67 68] and AMR markers will be detected by shotgun metagenomic sequencing. rRNA gene amplicon sequencing will be performed to determine microbial community composition and diversity[82 83]. The amplicon outputs will also be used for microbial source tracking using the SourceTracker tool[84 85]. Changes to the thermal environment will be assessed at the settlement level through remote sensed data supported by local thermal data and compared with changes to the built environment over time[86]. Continuous temperature tracking will use thermal dataloggers (Thermochron iButtons$^{TM}$). Ten randomly selected houses in each settlement will have thermal data loggers installed and a further 5 thermal data loggers will be deployed across the settlement to measure ambient temperature and humidity. Rainfall data will be derived from multiple (where available) local government-maintained weather stations enhanced with tipping bucket rain gauges installed to maximise coverage across the study area.

We will examine mosquito species variation and abundance over time based on quarterly trapping results. Mosquitoes will be sampled using BG Sentinel II traps[87] with 15 traps deployed per settlement, inside or outside randomly selected houses. Trap location will remain consistent throughout the assessment period. Mosquitoes will be identified to species under dissection microscopes using morphological keys[88-90]. Relative abundance and species composition of rodents in communal areas of settlements will also be surveyed in Makassar using standard traps and a grid-based trapping design to equate trapping effort across sites[91].The influence of season and physical environmental features, including temperature, humidity and rainfall, on the relative abundance of vector species will be examined[92 93]. Continuous passive acoustic monitoring at settlements will provide a measure of overall biodiversity[94]. The acoustic environment will be monitored using one or two (depending on settlement size) SongMeter SM4 bioacoustic recorders per site for human-audible sound frequencies. Additionally, in Indonesia we will deploy a single SongMeter SM4BAT recorder for ultrasonic frequencies emitted by echolocating bats at each site.

***Referral guidelines***

The study will refer participants for treatment to appropriate local healthcare providers in cases of acute malnutrition, anaemia, and intestinal helminth infection. WHO weight for length z-scores will be calculated in the field during anthropometry measurements for children under 5 years of age; children meeting WHO/UNICEF criteria for severe malnutrition (weight for length z-score ≤ 3) will be referred to a local health facility. At times of blood draws, all participating children under age 5 years will have their haemoglobin measured in the field. Children with severe anaemia (haemoglobin < 7.0g/dL) will be



For peer review only - http://bmjopen.bmj.com/site/about/guidelines.xhtml

referred to a local health facility. A subset of faeces samples will be immediately assessed for helminths using the Kato-Katz technique, and individuals with positive results will be referred for treatment.

**Randomisation**

Randomisation was performed separately for each city: March 2019 in Makassar and November 2019 in Suva (Figure 2). Randomisation occurred following settlement recruitment and completion of 12 months of baseline surveys. Settlements (clusters) have been randomly assigned to either intervention or control groups, with equal numbers in each group. Covariate-constrained randomisation was utilised using the cycrand package in Stata V15 to achieve balance between the intervention and control groups on key baseline factors determined *a priori*.

For Makassar, intervention and control groups were first balanced on number of children aged under 5 years (4 settlements in each group: ≤10, 11-30 and >30 children) and flood risk (high and low, 6 settlements in each), producing 90 allocations. Subsequently, the 30 allocations most imbalanced on average asset score were removed, with the score determined from each household's ownership of selected assets. For Suva, intervention and control groups were first balanced on number of children aged under 5 years (<40 children, 8 settlements; >40 children, 4 settlements) and flood risk and site contamination grouping (2 extreme risk, 2 high risk, and 8 medium risk settlements). This resulted in 120 allocations, from which the 30 most imbalanced allocations of asset score were removed, followed by the 30 most imbalanced allocations of average number of children under 5 years. For each of the cities, this resulted in the 60 best balanced allocations being used for the randomisation (total 3600 [60 × 60] possible allocations for the trial). A randomisation ceremony was held in both cities, where a child from the pilot community selected a ping pong ball from a large glass jar that contained 60 ping pong balls numbered 1 to 60, representing the 60 determined random allocations that achieve the balance criteria specified above.

**Sample size**

The choice of 12 settlements per city, each of ~55 dwellings with 5–6 people per dwelling, has been made to balance the need for statistical power with the constraints of construction logistics and cost. Statistical power was calculated using formulae for cluster RCTs with repeated assessments of a cohort[95] (and confirmed by numerical simulation using generalised linear mixed models) for the three primary health outcomes assessed in children aged under 5 years: i) prevalence and diversity of gastrointestinal pathogens present; ii) diversity and abundance of AMR markers; and iii) concentration of intestinal inflammation markers. For measures of diversity and abundance of markers of drug resistance in environmental samples, the power calculations assumed the same intra-cluster correlations as for the health outcomes (see below).

For the health outcomes involving subsampling of an average 30 children under 5 years of age per settlement, there is 80% power to detect, from 6 months post-construction and beyond, a 40% relative reduction in the prevalence of gastrointestinal pathogens (assuming a baseline prevalence of 25%) and a 31% relative reduction in the average count of enteric pathogens per child (assuming a baseline average count of 1.0 and overdispersion factor of 2; with a baseline of 2.0 the relative reduction is 23%), and at 12 months an absolute difference of 0.30 SDs (and 0.34 SDs for environmental assessments) in the average concentration of intestinal inflammation markers and average number and abundance of AMR markers. These calculations assume intra-cluster (i.e. within-settlement) correlations between: two children within the same time period 0.10[7]; two children in different time periods 0.067; and repeated measurements of the same child 0.10. The relative reduction of 30% has been informed by the findings reported in systematic reviews, which have found reductions of 30–50% due to water-quality interventions[96 97].



For peer review only - http://bmjopen.bmj.com/site/about/guidelines.xhtml

### Statistical analysis plan

Analyses will be by intention to treat. Descriptive statistics will report characteristics of settlements and of individuals by randomized arm. Estimation of the effects of the intervention for each outcome will use generalised estimating equations with an exchangeable correlation structure and robust standard errors clustered at settlement level, scaled with a small-sample degrees-of-freedom adjustment[98]. For binary and count outcomes, we will use a logarithmic link to estimate ratios of prevalence and mean counts, respectively; for continuous outcomes, the identity link will be used to estimate differences in means. All models will adjust for the baseline covariates, number of children under 5 years, flood risk category (see Randomisation section) and asset score. To enable temporal adjustment, indicator variables for seasonal patterns (e.g. rainy versus dry), local rainfall data for each site, and an indicator term for any major flood event at a particular settlement in a particular quarter will be included in the models. We will explore relationships between environmental changes and health outcomes by modelling health outcomes at 12-, 18- and 24-months post-construction, using changes in environmental measures at 6-, 12-, or 18-months post-construction, respectively, as principal covariates. We will estimate these effects using generalised estimating equations with pre-construction health and environmental parameters as baseline covariates aggregated at settlement-level, and will include interactions of environmental changes with the intervention for effect-modification assessment, together with adjustment for potential confounders.

Laboratory technicians conducting sample analyses will be to the intervention assignment blinded throughout the study duration through the use of barcodes for sample identification. Owing to the nature of the intervention, participants will not be blinded.

### Study oversight

The RISE study has an independent International Scientific Advisory Panel and a Governance Committee, both of which provide oversight and advice including in relation to any unintended effects of the trial intervention or trial conduct. The study also has a Data Advisory Panel, comprised of representatives across Monash University who provide strategic advice and operational guidance to assure a holistically-advised research data strategy.

### Limitations, justifications and additional analyses

Water and sanitation trials aiming for community-level intervention coverage pose great challenges for trial designers[99]. The relative lack of RCTs of such interventions reflects multiple inherent difficulties, including randomly allocating networked infrastructure, the long follow-up and large sample sizes required to study health outcomes, and the need for a complex engineering and construction phase[13]. The RISE study will deliver site-specific and bespoke interventions to each settlement, and while this is deliberate and a strength of our approach as it will address site-specific needs, it also results in a lack of intervention uniformity across communities. The purposive selection of settlements included and the relatively small size of each community compared with many informal settlement settings, while necessary from a logistics perspective, also limits broad external validity. Furthermore, while we intend to monitor the sustainability of the intervention, the RCT is funded to perform formal monitoring for an initial 2 years post intervention delivery.

Studies such as RISE which involve complex infrastructure upgrades face methodological evaluation challenges and cannot be performed in a blinded fashion, thereby potentially introducing biases in survey-based outcomes. For trials aimed at improving gastrointestinal health among children, the reliability of caregiver-reported diarrhoea is highly variable and in unblinded trials is particularly subject to bias[100]. An alternative approach of testing for organisms in human faeces therefore has advantages, but does not reliably distinguish between non-pathogenic carriage versus causal links of enteropathogens to diarrhoea[101] or impaired nutrient absorption and growth[102 103]. Hence, we will monitor a combination of primary objective outcomes as well as secondary clinical outcome biomarkers to assess children's



For peer review only - http://bmjopen.bmj.com/site/about/guidelines.xhtml

physical health. However, while we have described our planned methodologies for analytical testing, the multi-year timeline of the RISE trial means that analytical methods may improve during the study (for example, methods for measuring environmental enteric dysfunction), and accordingly we may need to make changes to the specific tests mentioned in this protocol. The results of our initial analyses to identify which pathogens and AMR genes are most prevalent in the study areas will inform decisions regarding optimal approaches for detection throughout the study period.

Defining primary versus secondary aims and outcomes from complex multifactorial community upgrades requires somewhat artificial prioritisation of transdisciplinary effects. This reflects that impacts of multi-dimensional interventions on communities occur along an interconnected causal chain rather than in a simple hierarchical and linear cause-and-effect fashion. While we have pre-specified our primary outcomes to reflect objective markers of gastrointestinal health in children under the age of 5 years, our fundamentally transdisciplinary intervention and assessment protocol appropriately accounts for the complex interconnections between ecosystem health, environmental contamination, vector abundance, physical health and wellbeing. Our intervention has potential to improve liveability through improved access and opportunities for use of community spaces, better economic sustainability through improved micro-economies and urban farming opportunities, and climate resilience through the diversification of water supplies and flood management. We will therefore assess a range of additional secondary, albeit equally important, impacts on environmental and ecological health, as well as on psychological, social and economic wellbeing outcomes, which we will measure at both at the individual and community level. Since RISE is a large study with many collaborators the research is expected to answer scientific questions beyond those posed in this protocol. Additionally, data and specimen collection tools may be added to address supplementary questions regarding health and environmental assessments in informal settlements.

## ETHICS AND DISSEMINATION

Ethics review and approval was provided by participating universities and local IRBs, including Monash University Human Research Ethics Committee (Melbourne, Australia; protocol 9396), the Ministry of Research, Technology and Higher Education Ethics Committee of Medical Research at the Faculty of Medicine, Universitas Hasanuddin (Makassar, Indonesia; protocol UH18020110), and the College Human Health Research Ethics Committee (CHREC) at the Fiji Institute of Pacific Health Research (FIPHR) and College of Medicine, Nursing, and Health Sciences at Fiji National University (FNU) (Suva, Fiji; protocol 137.19). Animal ethics for rodent trapping has also been secured (Monash University project ID16351; Universitas Hasanuddin UH18080446). The trial is registered on the Australian New Zealand Clinical Trials Registry (ANZCTR) (Trial ID: ACTRN12618000633280). All study settlements, households, and caregivers/respondents provide informed consent. The data collected in the study will be publicly distributed along with metadata and critical documents (i.e, protocols and questionnaires) following the publication of the primary results from the trials.

For peer review only



For peer review only - http://bmjopen.bmj.com/site/about/guidelines.xhtml

**References**

1. United Nations. Sustainable Development Goals Report, 2019, New York.. Accessed from https://unstats.un.org/sdgs/report/2019/The-Sustainable-Development-Goals-Report-2019.pdf, 13th June 2020.
2. Turley R, Saith R, Bhan N, et al. Slum upgrading strategies involving physical environment and infrastructure interventions and their effects on health and socio-economic outcomes. *Cochrane Database of Systematic Reviews* 2013(1.)
3. Ezeh A, Oyebode O, Satterthwaite D, et al. The history, geography, and sociology of slums and the health problems of people who live in slums. *The Lancet* 2017;389(10068):547-58.
4. United Nations. SDG Indicators, 2020. Accessed from https://unstats.un.org/sdgs/report/2019/Goal-11/, 13th June 2020.
5. Lilford RJ, Oyebode O, Satterthwaite D, et al. Improving the health and welfare of people who live in slums. *The Lancet* 2017;389(10068):559-70.
6. Weimann A, Oni T. A Systematised Review of the Health Impact of Urban Informal Settlements and Implications for Upgrading Interventions in South Africa, a Rapidly Urbanising Middle-Income Country. *International Journal of Environmental Research and Public Health* 2019;16(19):3608.
7. Clasen T, Boisson S, Routray P, et al. Effectiveness of a rural sanitation programme on diarrhoea, soil-transmitted helminth infection, and child malnutrition in Odisha, India: a cluster-randomised trial. *Lancet Glob Health* 2014;2(11):e645-53.
8. Luby SP, Rahman M, Arnold BF, et al. Effects of water quality, sanitation, handwashing, and nutritional interventions on diarrhoea and child growth in rural Bangladesh: a cluster randomised controlled trial. *Lancet Glob Health* 2018;6(3):e302-e15.
9. Null C, Stewart CP, Pickering AJ, et al. Effects of water quality, sanitation, handwashing, and nutritional interventions on diarrhoea and child growth in rural Kenya: a cluster-randomised controlled trial. *Lancet Glob Health* 2018;6(3):e316-e29.
10. Pickering AJ, Djebbari H, Lopez C, et al. Effect of a community-led sanitation intervention on child diarrhoea and child growth in rural Mali: a cluster-randomised controlled trial. *Lancet Glob Health* 2015;3(11):e701-11.
11. Brown R, Leder, K., Wong, T. et al. Improving human and environmental health in urban informal settlements: the Revitalising Informal Settlements and their Environments (RISE) programme *Lancet Planetary Health* 2018;2:S29.
12. Pickering AJ, Null C, Winch PJ, et al. The WASH Benefits and SHINE trials: interpretation of WASH intervention effects on linear growth and diarrhoea. *Lancet Glob Health* 2019;7(8):e1139-e46.
13. Cumming O, Arnold BF, Ban R, et al. The implications of three major new trials for the effect of water, sanitation and hygiene on childhood diarrhea and stunting: a consensus statement. *BMC Med* 2019;17(1):173.
14. Whitmee S, Haines A, Beyrer C, et al. Safeguarding human health in the Anthropocene epoch: report of The Rockefeller Foundation-Lancet Commission on planetary health. *Lancet* 2015;386(10007):1973-2028.
15. Chown SL, Duffy GA. Thermal physiology and urbanization: perspectives on exit, entry and transformation rules. *Functional Ecology* 2015;29(7):902-12.
16. Seto KC, Sánchez-Rodríguez R, Fragkias M. The New Geography of Contemporary Urbanization and the Environment. *Annual Review of Environment and Resources* 2010;35(1):167-94.
17. Elmqvist T, Fragkias M, Goodness J, et al. Urbanization, Biodiversity and Ecosystem Services: Challenges and Opportunities. Dordrecht, Germany: Springer 2013.
18. Seto KC, Güneralp B, Hutyra LR. Global forecasts of urban expansion to 2030 and direct impacts on biodiversity and carbon pools. *PNAS* 2012;109(40):16083-88.
19. Oke TR. The energetic basis of the urban heat island. *Quarterly Journal of the Royal Meteorological Society* 1982;108(455):1-24.
20. Peng S, Piao S, Ciais P, et al. Surface Urban Heat Island Across 419 Global Big Cities. *Environ Sci Technol* 2012;46(2):696-703.
21. Katukiza AY, Ronteltap M, Niwagaba CB, et al. Sustainable sanitation technology options for urban slums. *Biotechnology Advances* 2012;30(5):964-78.
22. Alirol E, Getaz L, Stoll B, et al. Urbanisation and infectious diseases in a globalised world. *Lancet Infect Dis* 2011;11(2):131-41.



For peer review only - http://bmjopen.bmj.com/site/about/guidelines.xhtml

23. Eisenstein M. Disease: Poverty and pathogens. *Nature* 2016;531(7594):S61-63.
24. Himsworth CG, Parsons KL, Jardine C, et al. Rats, cities, people, and pathogens: a systematic review and narrative synthesis of literature regarding the ecology of rat-associated zoonoses in urban centers. *Vector Borne Zoonotic Dis* 2013;13(6):349-59.
25. Franklinos LHV, Jones KE, Redding DW, et al. The effect of global change on mosquito-borne disease. *The Lancet Infectious Diseases* 2019;19(9):e302-e12.
26. Hassell JM, Begon M, Ward MJ, et al. Urbanization and Disease Emergence: Dynamics at the Wildlife–Livestock–Human Interface. *Trends in Ecology & Evolution* 2017;32(1):55-67.
27. Lau CL, Smythe LD, Craig SB, et al. Climate change, flooding, urbanisation and leptospirosis: fuelling the fire? *Trans R Soc Trop Med Hyg* 2010;104(10):631-38.
28. Carvalho S, Magalhães MdAFM, Medronho RdA. Analysis of the spatial distribution of dengue cases in the city of Rio de Janeiro, 2011 and 2012. *Rev Saude Publica* 2017;51:79.
29. Daudé É, Mazumdar S, Solanki V. Widespread fear of dengue transmission but poor practices of dengue prevention: A study in the slums of Delhi, India. *PLoS Med* 2017;12(2):e0171543.
30. Feleke H, Medhin G, Abebe A, et al. Enteric pathogens and associated risk factors among under-five children with and without diarrhea in Wegera District, Northwestern Ethiopia. *Pan Afr Med J* 2018;29:72.
31. Korpe PS, Valencia C, Haque R, et al. Epidemiology and Risk Factors for Cryptosporidiosis in Children From 8 Low-income Sites: Results From the MAL-ED Study. *Clin Infect Dis* 2018;67(11):1660-69.
32. Hagan JE, Moraga P, Costa F, et al. Spatiotemporal Determinants of Urban Leptospirosis Transmission: Four-Year Prospective Cohort Study of Slum Residents in Brazil. *PLoS Negl Trop Dis* 2016;10(1):e0004275.
33. Majorin F, Torondel B, Routray P, et al. Identifying Potential Sources of Exposure Along the Child Feces Management Pathway: A Cross-Sectional Study Among Urban Slums in Odisha, India. *The American Journal of Tropical Medicine and Hygiene* 2017;97(3):861-69.
34. World Health Organisation. Global antimicrobial resistance surveillance system (GLASS) report: Early implementation 2016-2017. Accessed from https://www.who.int/glass/resources/publications/early-implementation-report/en/, June 10th 2020.
35. Pehrsson EC, Tsukayama P, Patel S, et al. Interconnected microbiomes and resistomes in low-income human habitats. *Nature* 2016;533:212-16.
36. Nadimpalli ML, Marks SJ, Montealegre MC, et al. Urban informal settlements as hotspots of antimicrobial resistance and the need to curb environmental transmission. *Nat Microbiol* 2020;5(6):787-95.
37. Keusch GT, Rosenberg IH, Denno DM, et al. Implications of acquired environmental enteric dysfunction for growth and stunting in infants and children living in low- and middle-income countries. *Food Nutr Bull* 2013;34(3):357-64.
38. Naylor C, Lu M, Haque R, et al. Environmental Enteropathy, Oral Vaccine Failure and Growth Faltering in Infants in Bangladesh. *EBioMedicine* 2015;2(11):1759-66.
39. Vlahov D, Freudenberg N, Proietti F, et al. Urban as a Determinant of Health. *J Urban Health* 2007;84(Suppl 1):16-26.
40. Ferguson KT, Cassells RC, MacAllister JW, et al. The physical environment and child development: An international review. *International Journal of Psychology* 2013;48(4):437-68.
41. Guite HF, Clark C, Ackrill G. The impact of the physical and urban environment on mental well-being. *Public Health* 2006;120(12):1117-26.
42. Devoto F, Duflo E, Dupas P, et al. Happiness on Tap: Piped Water Adoption in Urban Morocco. *American Economic Journal: Economic Policy* 2012;4(4):68-99.
43. Evans GW. Child Development and the Physical Environment. *Annual Review of Psychology* 2006;57(1):423-51.
44. Lambert A, Vlaar J, Herrington S, et al. What Is the Relationship between the Neighbourhood Built Environment and Time Spent in Outdoor Play? A Systematic Review. *International Journal of Environmental Research and Public Health* 2019;16(20):3840.
45. Marx B, Stoker T, Suri T. The Economics of Slums in the Developing World. *Journal of Economic Perspectives* 2013;17(4):187-210.




For peer review only

46. Pieter van Dijk M, Etajak S, Mwalwega B, et al. Financing sanitation and cost recovery in the slums of Dar es Salaam and Kampala. *Habitat International* 2014;43:206-13.
47. Moreno EL. Slums of the world: The face of urban poverty in the new millennium. Nairobi, Kenya: UN-HABITAT, 2003.
48. Kadlac RH, Wallace SD. Treatment Wetlands, 2nd Ed. Florida, USA: CRC Press 2008.
49. Wu S, Carvalho PN, Müller JA, et al. Sanitation in constructed wetlands: A review on the removal of human pathogens and fecal indicators. *Science of The Total Environment* 2016;541:8-22.
50. Zhao Z, Song X, Wang Y, et al. Effects of algal ponds on vertical flow constructed wetlands under different sewage application techniques. *Ecological Engineering* 2016;93:120-28.
51. Bratieres K, Fletcher TD, Deletic A, et al. Nutrient and sediment removal by stormwater biofilters: A large-scale design optimisation study. *Water Research* 2008;42(14):3930-40.
52. Hatt BE, Fletcher TD, Deletic A. Hydraulic and Pollutant Removal Performance of Fine Media Stormwater Filtration Systems. *Environ Sci Technol* 2008;42(7):2535-41.
53. Kim H, Seagren EA, Davis AP. Engineered bioretention for removal of nitrate from stormwater runoff. *Water Environ Res* 2003;75(4):355-67.
54. Read J, Wevill T, Fletcher T, et al. Variation among plant species in pollutant removal from stormwater in biofiltration systems. *Water Research* 2008;42(4):893-902.
55. Davies CM, Bavor HJ. The fate of stormwater-associated bacteria in constructed wetland and water pollution control pond systems. *J Appl Microbiol* 2000;89(2):349-60.
56. García JA, Paredes D, Cubillos JA. Effect of plants and the combination of wetland treatment type systems on pathogen removal in tropical climate conditions. *Ecological Engineering* 2013;58:57-62.
57. Walker DJ, Hurl S. The reduction of heavy metals in a stormwater wetland. *Ecological Engineering* 2002;18(4):407-14.
58. Wong T, Breen PF, Somes NLG, et al. Managing Urban Stormwater using Constructed Wetlands, Industry Report 98/7. Melbourne, Australia: Cooperative Research Centre for Catchment Hydrology, 1998.
59. Austrade. China's Sponge City Program: Making China's cities watertight presents opportunities for Australian water technologies. Accessed from https://goo.gl/kHUcqh, May 10th 2020.
60. Cooperative Research Centre for Water Sensitive Cities. Kunshan awarded status as a National Ecological Garden City, 2016. Accessed from https://watersensitivecities.org.au/content/kunshan-awarded-status-national-ecological-garden-city/, May 8th 2020.
61. Jewish National Fund. New Bio-filters Purify Surface Runoff Water for Reuse, 2015. Accessed from http://www.kkl-jnf.org/about-kkl-jnf/green-israel-news/march-2015/biofilters-ramle-bat-yam, May 8th 2020.
62. O'Meara S. Why China Wants to Build Something Called Sponge Cities, CityLab, 2015. Accessed from https://www.citylab.com/design/2015/11/why-china-wants-to-build-sponge-cities/417114/, May 6th 2020.
63. Monitoring Singapore's first bioretention system: Rain garden at Balam Estate. In: Proceedings of Water Sensitive Urban Design; Building the Water Sensiitve Community, Melbourne, Australia, 2012.
64. Active, Beautiful and Clean (ABC) Waters Programme: towards sustainable stormwater management in Singapore. In: Proceedings of the 6th International Water Sensitive Urban Design Conference and Hydropolis, Perth, Australia, 2009.
65. Framework for Stormwater Quality Management in Singapore. Proceedings of the 12th International Conference on Urban Drainage, Porto Algere, Brazil, 2011.
66. A Dual-mode Biofilter System: Case study in Kfar Sava, Israel. Proceedings of the 12th International Conference on Urban Drainage, Porto Alegre, Brazil, 2011.
67. Liu J, Gratz J, Amour C, et al. A laboratory-developed TaqMan Array Card for simultaneous detection of 19 enteropathogens. *J Clin Microbiol* 2013;51(2):472-80.
68. Liu J, Platts-Mills JA, Juma J, et al. Use of quantitative molecular diagnostic methods to identify causes of diarrhoea in children: a reanalysis of the GEMS case-control study. *Lancet* 2016;388(10051):1291-301.
69. Wagner M, Peterson CGB, Ridefelt P, et al. Fecal markers of inflammation used as surrogate markers for treatment outcome in relapsing inflammatory bowel disease. *World J Gastroenterol* 2008;14(36):5584-89.



For peer review only - http://bmjopen.bmj.com/site/about/guidelines.xhtml

70. Bernier JJ, Florent C, Desmazures C, et al. Diagnosis of protein-losing enteropathy by gastrointestinal clearance of alpha1-antitrypsin. *Lancet* 1978;2(8093):763-64.
71. Campbell DI, McPhail G, Lunn PG, et al. Intestinal inflammation measured by fecal neopterin in Gambian children with enteropathy: association with growth failure, Giardia lamblia, and intestinal permeability. *J Pediatr Gastroenterol Nutr* 2004;39(2):153-57.
72. Overbey KN, Schwab KJ, Exum NG. Comparison of 1-week and 2-week recall periods for caregiver-reported diarrhoeal illness in children, using nationally representative household surveys. *Int J Epidemiol* 2019;48(4):1228-39.
73. de Onis M, Onyango AW, Van den Broeck J, et al. Measurement and standardization protocols for anthropometry used in the construction of a new international growth reference. *Food Nutr Bull* 2004;25(1 Suppl):S27-36.
74. Ayele B, Aemere A, Gebre T, et al. Reliability of measurements performed by community-drawn anthropometrists from rural Ethiopia. *PLOS ONE* 2012;7(1):e30345.
75. Varni JW, Seid M, Kurtin PS. PedsQL 4.0: reliability and validity of the Pediatric Quality of Life Inventory version 4.0 generic core scales in healthy and patient populations. *Med Care* 2001;39(8):800-12.
76. Krueger AB, Stone AA. Progress in measuring subjective well-being. *Science* 2014;436(42)
77. Organisation for Economic Co-operation and Development. OECD Guidelines on Measuring Subjective Wellbeing. Paris, France: OECD Publishing, 2013.
78. Grootaert C, Narayan D, Woolcock M, et al. Measuring social capital: an integrated questionnaire. Washington DC: World Bank, 2004.
79. Henry R, Schang C, Chandrasena GI, et al. Environmental monitoring of waterborne Campylobacter: evaluation of the Australian standard and a hybrid extraction-free MPN-PCR method. *Front Microbiol* 2015;6:74.
80. Jones NR, Millman C, van der Es M, et al. Novel Sampling Method for Assessing Human-Pathogen Interactions in the Natural Environment Using Boot Socks and Citizen Scientists, with Application to Campylobacter Seasonality. *Appl Environ Microbiol* 2017;83(14):e00162-17.
81. Schang C, Henry R, Kolotelo PA, et al. Evaluation of Techniques for Measuring Microbial Hazards in Bathing Waters: A Comparative Study. *PLOS ONE* 2016;11(5):e0155848.
82. Caporaso JG, Lauber CL, Walters WA, et al. Ultra-high-throughput microbial community analysis on the Illumina HiSeq and MiSeq platforms. *ISME J* 2012;6(8):1621-24.
83. Caporaso JG, Lauber CL, Walters WA, et al. Global patterns of 16S rRNA diversity at a depth of millions of sequences per sample. *Proc Natl Acad Sci USA* 2011;108 Suppl 1:4516-22.
84. Henry R, Schang C, Coutts S, et al. Into the deep: Evaluation of SourceTracker for assessment of faecal contamination of coastal waters. *Water Research* 2016;93:242-53.
85. Knights D, Kuczynski J, Charlson ES, et al. Bayesian community-wide culture-independent microbial source tracking. *Nat Methods* 2011;8(9):761-3.
86. Fu P, Weng Q. A time series analysis of urbanization induced land use and land cover change and its impact on land surface temperature with Landsat imagery. *Remote Sensing of Environment* 2016;175:205-14.
87. Williams CR, Long SA, Webb CE, et al. Aedes aegypti Population Sampling Using BG-Sentinel Traps in North Queensland Australia: Statistical Considerations for Trap Deployment and Sampling Strategy. *J Med Entomol* 2007;44(2):345-50.
88. Huang YM. The mosquitoes of Polynesia with a pictorial key to some species associated with filariasis and/or dengue fever. *Mosquito Systematics* 1977;9(3):289-322.
89. Reuben R, Tewari SC, Hiriyan J, et al. Illustrated keys to species of Culex (Culex) associated with Japanese encephalitis in Southeast Asia (Diptera: Culicidae). *Mosquito systematics* 1994;26(2):75-96.
90. Rueda L. Pictorial keys for the identification of mosquitoes (Diptera: Culicidae) associated with Dengue Virus Transmission. *Zootaxa* 2004;589.
91. Aplin KP, Brown PR, Jacob J, et al. Field methods for rodent studies in Asia and the Pacific. Canberra, Australia: Australian Centre for International Agricultural Research, 2003.
92. Himsworth CG, Jardine CM, Parsons KL, et al. The Characteristics of Wild Rat (Rattus spp.) Populations from an Inner-City Neighborhood with a Focus on Factors Critical to the Understanding of Rat-Associated Zoonoses. *PLOS ONE* 2014;9(3)



For peer review only - http://bmjopen.bmj.com/site/about/guidelines.xhtml

93. Mordecai EA, Caldwell JM, Grossman MK, et al. Thermal biology of mosquito-borne disease. *Ecology Letters* 2019;22(10):1690-708.
94. Bradfer-Lawrence T, Gardner N, Bunnefeld L, et al. Guidelines for the use of acoustic indices in environmental research. *Methods in Ecology and Evolution* 2019;10(10):1796-807.
95. Hooper R, Teerenstra S, de Hoop E, et al. Sample size calculation for stepped wedge and other longitudinal cluster randomised trials. *Stat Med* 2016;35(26):4718-28.
96. Clasen TF, Alexander KT, Sinclair D, et al. Interventions to improve water quality for preventing diarrhoea. *Cochrane Database Syst Rev* 2015(10):CD004794.
97. Ziegelbauer K, Speich B, Mausezahl D, et al. Effect of sanitation on soil-transmitted helminth infection: systematic review and meta-analysis. *PLoS Med* 2012;9(1):e1001162.
98. Mancl LA, DeRouen TA. A covariance estimator for GEE with improved small-sample properties. *Biometrics* 2001;57(1):126-34.
99. Schmidt WP. The elusive effect of water and sanitation on the global burden of disease. *Trop Med Int Health* 2014;19(5):522-7.
100. Luby SP, Agboatwalla M, Hoekstra RM. The variability of childhood diarrhea in Karachi, Pakistan, 2002-2006. *The American Journal of Tropical Medicine and Hygiene* 2011;84(6):870-77.
101. Taniuchi M, Sobuz SU, Begum S, et al. Etiology of diarrhea in Bangladeshi infants in the first year of life analyzed using molecular methods. *J Infect Dis* 2013;208(11):1794-802.
102. Kau AL, Planer JD, Liu J, et al. Functional characterization of IgA-targeted bacterial taxa from undernourished Malawian children that produce diet-dependent enteropathy. *Sci Transl Med* 2015;7(276):276ra24.
103. Smith MI, Yatsunenko T, Manary MJ, et al. Gut microbiomes of Malawian twin pairs discordant for kwashiorkor. *Science* 2013;339(6119):548-54.

For peer review only

**Authors' contributions**

Devised the project's main conceptual ideas: KL, PA, TC, SLC, ABF, MF, DJ, DRL, SPL, DM, DR, JAS, TW, RB
Contributed to development of protocol details and implementation: KL, JO, AA, SFB, KB, TC, SLC, GAD, PAF, GF, MF, CG, RH, EH, DJ, RL, AL, SPL, DM, JO'T, DRL, SSS, RS, RT, AT, ART TW, RB
Designed the statistical analysis plan: ABF and JAS
Main drafting of the main manuscript: KL, JO
Critical review and approval of the manuscript: All authors

**Funding:** This trial has been primarily funded by the Wellcome Trust "Our Planet, Our Health" grant 205222/Z/16/Z. The intervention is funded by the Asian Development Bank, the New Zealand Ministry of Foreign Affairs and Trade, and Monash University. The funders have no role in the study design, data collection and analysis, decision to publish, or preparation of manuscripts.

**Competing interests:** None

**Acknowledgements:** We acknowledge the hard work and dedication of the RISE project field teams in Fiji and Indonesia, and all the study participants.

For peer review only

Figure 1: The RISE study hypothesised theory of impacts. The water sensitive intervention will improve the physical environmental conditions, resulting in both direct and indirect impacts on ecology and human physical health and wellbeing

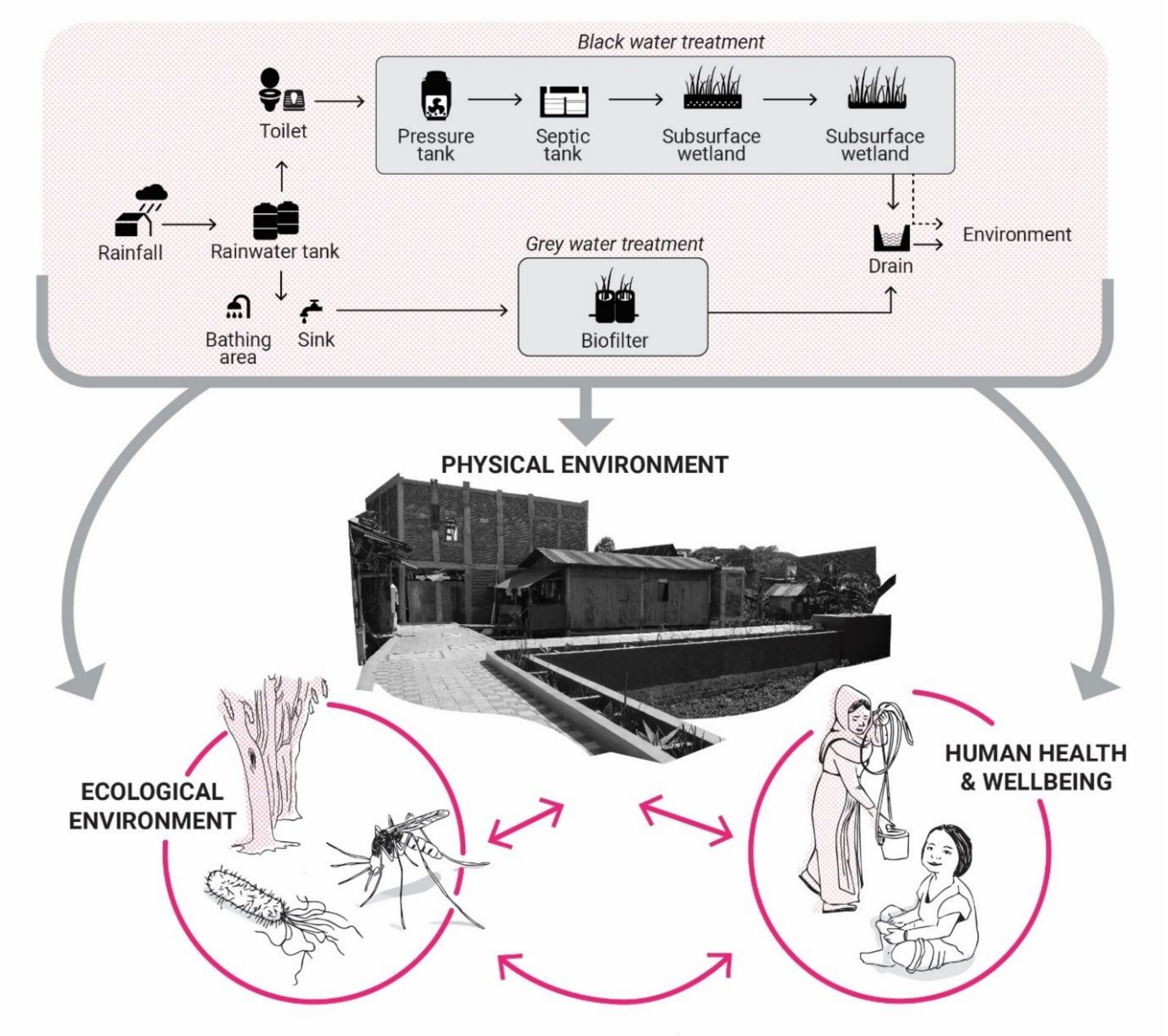

Figure 2: Anticipated RISE study timeline

| | | 2017 | 2018 | 2019 | 2020 | 2021 | 2022 | 2023 | 2024 |
|---|---|---|---|---|---|---|---|---|---|
| | | YEAR 1 | Y2 | Y3 | Y4 | Y5 | Y6 | Y7 | Y8 |
| Indonesia | Intervention | Collection of data on site characteristics, government approvals, protocols development<br>Final site selection, community mobilisation and government approvals | | Intervention sites - co-design, tendering, approvals | Intervention sites - construction | | | Control sites - co-design | Control sites - construction |
| Indonesia | Assessment | | Participant consent<br>Baseline data collection; Randomisation (Mar 2019) | Monitoring across all sites | | | Post-construction monitoring across all sites | | |
| Fiji | Intervention sites | Final site selection, community mobilisation and government approvals | | Intervention sites - co-design, tendering, approvals | Intervention sites - construction | | | Control sites - co-design | Control sites - construction |
| Fiji | Assessment | | Participant consent<br>Baseline data collection; Randomisation (Nov 2019) | Monitoring across all sites | | | Post-construction monitoring across all sites | | |

For peer review only - http://bmjopen.bmj.com/site/about/guidelines.xhtml

Figure 3: Schematics of proposed sanitation intervention

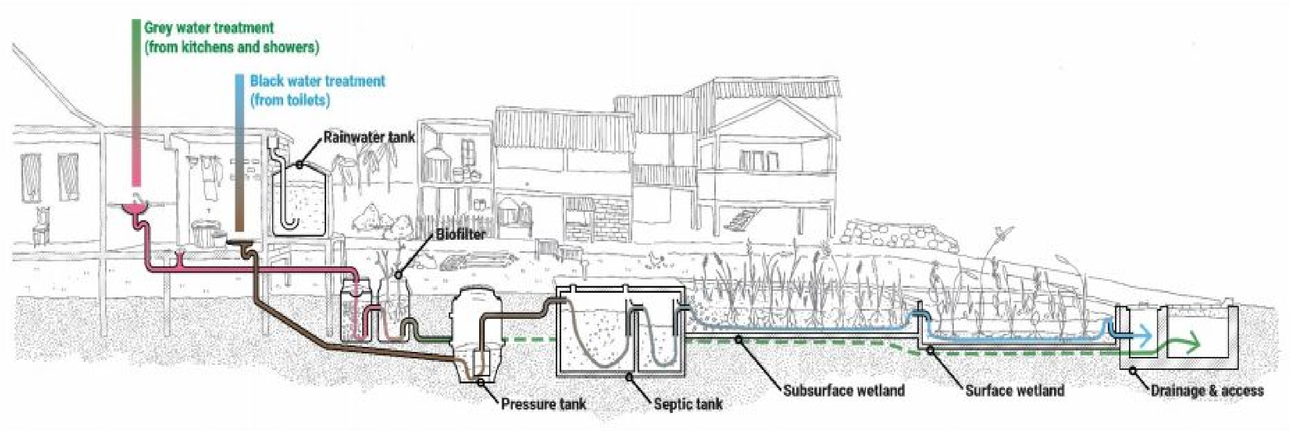


For peer review only

## Is participation voluntary?

Yes, participation in the study is voluntary. You can choose not to participate at all, participate in only some components or in all components of the study.

## How will the consent form be used?

If you are willing to take part in the study, we would like you to provide your consent (CONSENT FORM 1) by verbally agreeing and by signing on the electronic form provided. We will also mark on a paper consent form the statements that you have agreed to, and give you a copy to keep.

## How will my privacy be protected?

All of the answers you give to surveys and results of any sample analyses or measurements taken will be confidential and will not be shared with anyone other than the members of the study team. We will use codes to identify your household and persons within the household. All code information will be stored securely.

Each Tablet used to collect survey and other data will have an encrypted file system with password access. This means that if the Tablet is lost, no one without the password will be able to access the contents of the Tablet. Access to stored data will be password controlled by researchers and any data with any identifying information will be encrypted during transfer.

## Are results of the study to be published?

At the completion of the program, research findings will be published in papers in scientific journals and presented at international conferences but not in a form that will allow individual persons or households to be identified.

## What if I have any questions about the study?

Should you have any questions about the study, please contact:

**Isoa Vakarewa**
Live & Learn Fiji
**T** +679 943 8443
**E** isoa.vakarewa@livelearn.org

## How can I lodge a complaint?

Should you have a complaint concerning the manner in which this research is conducted, please refer to project MUHREC 9396 and contact:

**Petero Vatu**
Live & Learn Fiji
**T** +679 3315467
**E** petero.vatu@livelearn.org

MONASH University

rise
REVITALISING INFORMAL SETTLEMENTS AND THEIR ENVIRONMENTS

# Household Participation

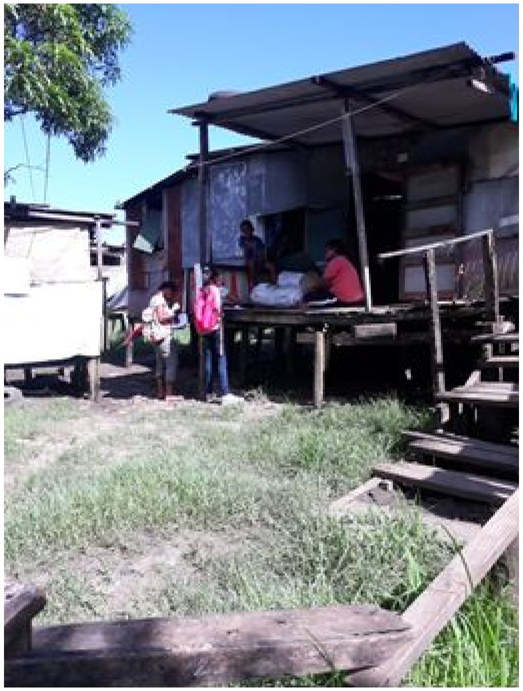

For peer review only - http://bmjopen.bmj.com/site/about/guidelines.xhtml

## What is this study about?

This research study is named the RISE (Revitalising Informal Settlements and their Environment) Study. In this study we are using novel engineering solutions (also called a 'water-sensitive design' approach), and we will examine whether this approach to upgrading water and sanitation management leads to improved environmental and public health outcomes. The water-sensitive design approach includes nature-based systems such as sub-surface wetlands, wetlands and rain gardens.

## Why has my household been invited to take part in the study?

Twelve settlements in Suva, Fiji have been selected for the RISE Study. Your settlement has been chosen for the study because of past problems of water flooding following heavy rain or coastal events; the size and number of households in your settlement, and the fact that your settlement is geographically well suited to the intervention.

## What does participation in the study as a household involve?

Participation in this research program will include the following:

a) Receiving the engineering intervention either in the next 2 years (intervention group – tranche 1) or 2 years later at the end of the study ('control' group – tranche 2).
b) We will attach an identifying number to the outside of your house to help researchers identify your household when they conduct their visits.
c) We will ask you to complete a household survey every 3 months over the period 2018 – 2022. Researchers will visit you and ask you some general questions about the household, as well as questions about your health and wellbeing. The time required for each survey will vary because different combinations of questions will be asked each time. However, your household can generally expect that all surveys will take no more than an hour (except the first survey which may take longer).
d) We may ask to collect water, wastewater, soil and other samples from in and around your house. Surveyors may also ask to take samples from household surfaces such as floors and tables.
e) We will ask whether you would allow photos of how your household stores water (and possibly some photos of other household items) to be taken. This would be done at the same time as surveys are administered or environmental samples are collected.
f) We may ask whether you would allow attachment of temperature monitoring devices ("i-buttons") inside or near your home.
g) We may ask whether you would allow mosquito traps to be installed inside or near your home.
h) We may ask whether you would allow trapping of rodents or rodent activity monitoring around your home.
i) We will ask for your permission to contact you if you move from the settlement anytime during the 5-year program period. If you provide permission. researchers will contact you following your departure from the settlement and invite you to complete a survey about the reasons you moved.
j) We will ask for your permission to contact you following extreme events (such as extreme climatic events). If you provide permission, researchers may contact you and invite you to complete a survey about the impact of such events on your well-being.
k) We may ask for your permission to undertake some additional surveys using SMS technology. If you provide permission, at some point during the study your household may be invited to answer some additional questions via SMS.

## When can I expect changes to my settlement to occur?

The timing of changes at both a community and household level will depend upon whether your settlement is selected to receive the water and sewage infrastructure in the next 2 years (intervention group – tranche 1), or at the end of the study period (control group – tranche 2). Selection of each settlement to the first or second group will be done RANDOMLY.

All 12 settlements will be recruited as a first step. The random selection of each settlement to the first or second group will then be made, and you will be told which group you belong to. All 12 chosen settlements will receive the upgrade.

For peer review only - http://bmjopen.bmj.com/site/about/guidelines.xhtml

rise
REVITALISING INFORMAL SETTLEMENTS AND THEIR ENVIRONMENTS

# CONSENT FORM 1

(GROUP 2: Households)

**Program:** RISE (Revitalising Informal Settlements and their Environments) Study
**Chief Investigator:** Professor Karin Leder

I have been asked to take part in the RISE research program. I have read and understood the explanatory material and I hereby consent, on behalf of my household, to their participation in this program.

| I consent to: | Yes | No |
|---|---|---|
| a) Participation in the 5-year research program, which will include an engineering intervention either during the research program (intervention group – tranche 1) or 2 years later at the end of the study (‘control’ group – tranche 2). | ☐ | ☐ |
| b) We will attach an identifying number to the outside of your house to help researchers identify your household when they conduct their visits. | ☐ | ☐ |
| c) My household taking part in surveys to be administered 3-monthly at my home over the period 2018 – 2022. | ☐ | ☐ |
| d) Collection of samples of water, wastewater, soil and other objects or surfaces in or around my home. | ☐ | ☐ |
| e) Photography of household water storage vessels / household items / house exterior during surveying or when environmental samples are collected. | ☐ | ☐ |
| f) If requested, allow attachment of temperature monitoring devices (“i-buttons”) inside or around my home. | ☐ | ☐ |
| g) If requested, allow mosquito traps to be installed inside or around my home. | ☐ | ☐ |
| h) If requested, allow trapping of rodents or rodent activity monitoring around my home. | ☐ | ☐ |
| i) Allowing researchers to contact me if I leave the settlement and move to live elsewhere. | ☐ | ☐ |
| j) Allowing researchers to contact me after extreme events (such as extreme climatic events) that might impact on my home. | ☐ | ☐ |
| k) Allowing researchers to contact me to invite me to participate in SMS surveys that may arise during the study period. | ☐ | ☐ |

**Contact details (where consent to i, j or k) is given:**

(Mobile telephone number)…………………………………………………………………………………..

**Name of Participant** ____________________

**Participant Signature** ____________________ **Date** __________

For peer review only

### What if I have any questions about the study?

Should you have any questions about the study, please contact:

**Isoa Vakarewa**
Live & Learn Fiji
**T** +679 943 8443
**E** isoa.vakarewa@livelearn.org

### How can I lodge a complaint?

Should you have a complaint concerning the manner in which this research is conducted, please refer to project MUHREC 9396 and contact:

**Petero Vatu**
Live & Learn Fiji
**T** +679 3315467
**E** petero.vatu@livelearn.org

MONASH University

rise

REVITALISING INFORMAL SETTLEMENTS AND THEIR ENVIRONMENTS

# Participation of children less than 5-Years Old

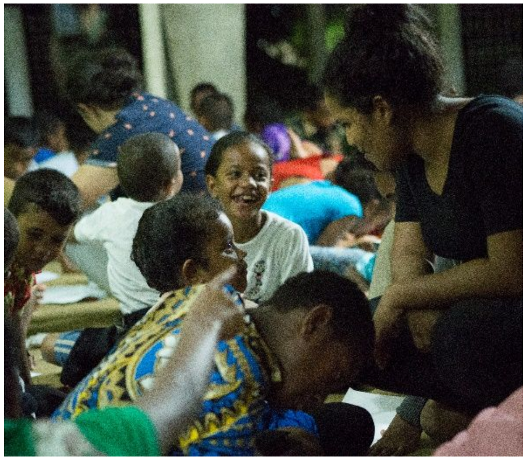

For peer review only - http://bmjopen.bmj.com/site/about/guidelines.xhtml

## What does participation in the study involve for my child(ren) less than 5 years old?

Formal parental or guardian signed consent is requested for the collection and analysis of blood and faecal samples, permission for height and weight measurements, and access to health records for children less than 5 years old, with details as follows:

a) Collection of faecal samples 3-monthly. This involves the collection of freshly passed stool.

   Where a faecal sample is not immediately available at the time of the household visit, the parent/caregiver will be given instructions on how to collect a faecal sample and provided with a sample collection kit. The sample will then be picked up by field workers.

b) Collection of venous blood samples once a year by a person medically trained to take blood, and subsequent analysis of these samples.

c) Measurement of the height and weight of children under 5 years old once a year. This will be performed by a trained person and will be conducted in your presence.

d) Faecal and venous blood samples collected as part of this program will be analysed and summary results will be reported back to the community and in scientific papers at the end of the study. No reports will allow identification of the individual person from whom samples were taken.

   Some of these samples will be frozen and retained and may be used by named researchers in this program in future research programs, for which ethics approval will be sought. You are asked to consent to this use of stored collected samples in future studies. You are also asked to consent to use of data or-further examination from samples already analysed if new techniques to obtain (better) results become available.

e) Consent for access to records of children aged less than 5 years since their birth which contains information on their vaccinations, presentations to healthcare for illnesses and growth records.

## Is participation voluntary?

Yes, participation in the study is voluntary. You can choose at any time to withdraw your child(ren) from some or all components of the study.

## What are possible risks to my child(ren)?

Collecting blood and faecal samples are minimal-risk procedures. Blood collection will be conducted by experienced, qualified staff employing appropriate procedures to reduce risks to children, and will follow the "World Health Organisation guidelines on drawing blood".

## How will the consent form be used?

If you are willing to take part in the study, we would like you to provide your consent (CONSENT FORM 3) by verbally agreeing and by signing on the electronic form provided. We will also mark on a paper consent forms the statements that you have agreed to, and give you a copy to keep.

## How will my privacy be protected?

All of the answers you give to surveys and results of any sample analyses or measurements taken will be confidential and will not be shared with anyone other than the members of the study team. We will use codes to identify your household and persons within the household. All code information will be stored securely.

## Are results of the study to be published?

At the completion of the program, research findings will be published in papers in scientific journals and presented at international conferences but not in a form that will allow individual persons or households to be identified.

## Will I get payment for participating in the study?

No, there will be no direct payment for study participation.

rise

REVITALISING INFORMAL SETTLEMENTS AND THEIR ENVIRONMENTS

# CONSENT FORM 3

(GROUP 4: Children less than 5years old)

**Program:** RISE (Revitalising Informal Settlements and their Environments) Study

**Chief Investigator:** Professor Karin Leder

I have been asked to take part in the RISE research program specified above. I have read and understood the Explanatory material and I hereby consent to the participation of my children in this program.

| I consent my child(ren) as named below, together with their age (year of birth), to participate in the following: | Yes | No |
|---|---|---|
| **CHILDREN LESS THAN 5 YEARS OLD** | | |
| a) Up to quarterly collection of faecal samples from my child and subsequent analysis of samples | ☐ | ☐ |
| b) Collection of venous blood samples once per year and subsequent analysis of samples. | ☐ | ☐ |
| c) Yearly measurement of height and weight | ☐ | ☐ |
| d) The faecal and blood samples provided during this research, and associated data, may be used by named researchers in this program in future research programs | ☐ | ☐ |
| e) Researchers accessing local health centre records for vaccination, medical presentations and growth measurements since the birth of my child | ☐ | ☐ |

| **CHILDREN < 5 YEARS OLD** | |
|---|---|
| **NAME** | **AGE (Year of birth)** |
| | |
| | |
| | |
| | |
| | |

Name of Parent / Guardian ______________________________

Parent / Guardian Signature ______________________ Date ______________

For peer review only

**Checkllist SPIRIT -complete**

| Item no | Description | Information | Found on what Page of the protocol? - current Page numbers |
|---|---|---|---|
| 1 | Descriptive title identifying the study design, population, interventions, and, if applicable, trial acronym | All information in current title: "Study design, rationale and assessment methods of the Revitalising Informal Settlements and their Environments (RISE) study: a cluster randomised controlled trial to evaluate a human health impacts of a water sensitive intervention in informal settlements in Indonesia and Fiji" | Pg. 1 |
| 2a | Trial identifier and registry name. If not yet registered, name of intended registry | Registration: Australian New Zealand Clinical Trials Registry (ANZCTR) (Trial ID: ACTRN12618000633280). | Abstract (Page 2) and 'Ethics and Dissemination' section (Page 15) |
| 2b | All items from the World Health Organization Trial Registration Data Set | See WHO Trial Registration dataset collection | See WHO Trial Registration dataset collection |
| 3 | Date and version identifier | Article submission will be of version 1 with date of submission identifier | Version 1; 16/07/2020 |
| 4 | Sources and types of financial, material, and other support | See text prepared for end of article: Funding: This trial has been primarily funded by the Wellcome Trust "Our Planet, Our Health" grant 205222/Z/16/Z | See Funding (Pg.21) |
| 5a | Names, affiliations, and roles of protocol contributors | | Pages 1 & 21 |
| 5b | Name and contact information for the trial sponsor | Professor Rebekah Brown Senior Vice-Provost & Vice-Provost (Research) and Program Director RISE Revitalising Informal Settlements and their Environments (RISE) Monash University Office of the Provost and Senior Vice-President Chancellery 27 Chancellors Walk Clayton VIC 3800 Australia T: +61 3 9905 2122 E: rebekah.brown@monash.edu | See WHO Trial Registration data set |
| 5c | Role of study sponsor and funders, if any, in study design; collection, management, analysis, and interpretation of data; writing of the report; and the decision to submit the report for publication, including whether they will have ultimate authority over any of these activities | The funders have no role in study design, data collection and analysis, decision to publish, or preparation of manuscripts.' | See 4 above (Pg. 21) |
| 5d | Composition, roles, and responsibilities of the coordinating centre, steering committee, endpoint adjudication committee, data management team, and other individuals or groups overseeing the trial, if applicable (see Item 21a for data monitoring committee) | See Study oversight section | See Page 14 |
| 6a | Description of research question and justification for undertaking the trial, including summary of relevant studies (published and unpublished) examining benefits and harms for each intervention | This information is included in Introduction, Hypotheses, and Aims | Pg. 4, 5, 6 |
| 6b | Explanation for choice of comparators | Yes – clearly explained in Study Methods and Analysis: "RISE is a parallel-cluster RCT | Pg. 6 |

For peer review only

| | | | |
|---|---|---|---|
| | | involving 12 informal settlements in Suva, Fiji, and 12 in Makassar, Indonesia. In each country, half of the sites have been randomised to receive an early upgrade ('intervention' communities), and the remaining will serve as controls". | |
| 7 | Specific objectives or hypotheses | See Hypotheses, and Aims | Pg. 4, 5, 6 |
| 8 | Description of trial design including type of trial (eg, parallel group, crossover, factorial, single group), allocation ratio, and framework (eg, superiority, equivalence, noninferiority, exploratory) | Parallel design in title and throughout (superiority trial framework)<br>Statistical plan | Pg. 14 |
| 9 | Description of study settings (eg, community clinic, academic hospital) and list of countries where data will be collected. Reference to where list of study sites can be obtained | See Study settings | Pg. 6,7 |
| 10 | Inclusion and exclusion criteria for participants. If applicable, eligibility criteria for study centres and individuals who will perform the interventions (eg, surgeons, psychotherapists) | Inclusion and exclusion criteria given in section 'Settlement eligibility and selection criteria" and provided in Table 1 | Pg 7 |
| 11a | Interventions for each group with sufficient detail to allow replication, including how and when they will be administered | The intervention is fully described in Section 'Description of the intervention' – including codesign which allows for some variation between sites | Pg 8,9 |
| 11b | Criteria for discontinuing or modifying allocated interventions for a given trial participant (eg, drug dose change in response to harms, participant request, or improving/worsening disease) | Not applicable as the intervention is being implemented at the community level | NA |
| 11c | Strategies to improve adherence to intervention protocols, and any procedures for monitoring adherence (eg, drug tablet return, laboratory tests) | Long-term operation and maintenance responsibilities will be addressed via training and ongoing engagement of community representatives and local authorities. | Pg. 9 |
| 11d | Relevant concomitant care and interventions that are permitted or prohibited during the trial | Standard hygiene messages will be provided to households in both early- and delayed-intervention communities | NA |
| 12 | Primary, secondary, and other outcomes, including the specific measurement variable (eg, systolic blood pressure), analysis metric (eg, change from baseline, final value, time to event), method of aggregation (eg, median, proportion), and time point for each outcome. Explanation of the clinical relevance of chosen efficacy and harm outcomes is strongly recommended | All primary and secondary outcomes are given under the Outcomes heading on Page 8 and onwards. The specific measurement variables are stated in Table 2<br>We state that presence, abundance and diversity will be compared. We detail the time point for each outcome.<br>We describe median values for height / weight measurements but otherwise method of aggregation in outcomes section; otherwise details in sample size section.<br>Action levels (harm) for Hb and Z score plus helminth given in 'Referral guidelines' Page 13/14 | Table 2 Page 9- 10<br>Outcomes section, Pages 11, 12<br>Referral guidelines Page 12,13<br>Sample size Page 13 |
| 13 | Time schedule of enrolment, interventions (including any run-ins and washouts), assessments, and visits for participants. A schematic | Schematic diagram is provided | See Fig 2 |

For peer review only

| | | | |
|---|---|---|---|
| | diagram is highly recommended (see Figure) | | |
| 14 | Estimated number of participants needed to achieve study objectives and how it was determined, including clinical and statistical assumptions supporting any sample size calculations | See sample size section | Page 13 |
| 15 | Strategies for achieving adequate participant enrolment to reach target sample size | Community cooperation was obtained prior to enrolment of HH– see section Site and HH recruitment Page 8. | Page 8 |
| 16a | Method of generating the allocation sequence (eg, computer-generated random numbers), and list of any factors for stratification. To reduce predictability of a random sequence, details of any planned restriction (eg, blocking) should be provided in a separate document that is unavailable to those who enrol participants or assign interventions | See Section 'Randomisation' | Page 13 |
| 16b | Mechanism of implementing the allocation sequence (eg, central telephone; sequentially numbered, opaque, sealed envelopes), describing any steps to conceal the sequence until interventions are assigned | See 'Randomisation' section | Page 13 |
| 16c | Who will generate the allocation sequence, who will enrol participants, and who will assign participants to interventions | Statisticians generated the allocation sequence (see details in 'Randomisation section).<br>'Household recruitment was performed by trained local community field worker teams fluent in local languages.' | Page 13<br><br>Page 8 |
| 17a | Who will be blinded after assignment to interventions (eg, trial participants, care providers, outcome assessors, data analysts), and how | Lab technicians will be blinded through the use of barcodes for sample identification' | Page 14 |
| 17b | If blinded, circumstances under which unblinding is permissible, and procedure for revealing a participant's allocated intervention during the trial | Not applicable: no blinding of participants | Not applicable |
| 18a | Plans for assessment and collection of outcome, baseline, and other trial data, including any related processes to promote data quality (eg, duplicate measurements, training of assessors) and a description of study instruments (eg, questionnaires, laboratory tests) along with their reliability and validity, if known. Reference to where data collection forms can be found, if not in the protocol | Human anthropometrics will be collected as triplicate measurements –<br>Local trained community field are employed<br>Validated Questionnaires to be used | Page 12<br><br>Page 8<br>Page 12 |
| 18b | Plans to promote participant retention and complete follow-up, including list of any outcome data to be collected for participants who discontinue or deviate from intervention protocols | To maintain participation over the trial period small culturally appropriate gifts will be provided. | Page 9 |

For peer review only

| | | | |
|---|---|---|---|
| 19 | Plans for data entry, coding, security, and storage, including any related processes to promote data quality (eg, double data entry; range checks for data values). Reference to where details of data management procedures can be found, if not in the protocol | Details available in Ethics application (available on request) | Details available in Ethics application (available on request) |
| 20a | Statistical methods for analysing primary and secondary outcomes. Reference to where other details of the statistical analysis plan can be found, if not in the protocol | See statistical section | Page 14 |
| 20b | Methods for any additional analyses (eg, subgroup and adjusted analyses) | See statistical section | Page 14 |
| 20c | Definition of analysis population relating to protocol non-adherence (eg, as randomised analysis), and any statistical methods to handle missing data (eg, multiple imputation) | Will be analysed as intention to treat | Page 14 |
| 21a | Composition of data monitoring committee (DMC); summary of its role and reporting structure; statement of whether it is independent from the sponsor and competing interests; and reference to where further details about its charter can be found, if not in the protocol. Alternatively, an explanation of why a DMC is not needed | See study oversight section<br>The RISE study has an independent International Scientific Advisory Panel and a Governance Committee, both of which provide oversight and advice including in relation to any unintended effects of the trial intervention. The study also has a Data Advisory Panel, comprised of representatives across Monash University who provide strategic advice and operational guidance to assure a holistically-advised research data strategy. | Pg 14 |
| 21b | Description of any interim analyses and stopping guidelines, including who will have access to these interim results and make the final decision to terminate the trial | See Study Oversight | Page 14 |
| 22 | Plans for collecting, assessing, reporting, and managing solicited and spontaneously reported adverse events and other unintended effects of trial interventions or trial conduct | See study oversight section<br><br>"The RISE study has an independent International Scientific Advisory Panel and a Governance Committee, both of which provide oversight and advice, including in relation to any unintended effects of the trial intervention or trial conduct. | Page 14 |
| 23 | Frequency and procedures for auditing trial conduct, if any, and whether the process will be independent from investigators and the sponsor | See study oversight section | Page 14 |
| 24 | Plans for seeking research ethics committee/institutional review board (REC/IRB) approval | See 'Ethics and dissemination section' | Page 15 |
| 25 | Plans for communicating important protocol modifications (eg, changes to eligibility criteria, outcomes, analyses) to relevant parties (eg, investigators, REC/IRBs, trial participants, trial registries, journals, regulators) | Trial registries, investigators and human research ethics committees are notified of changes, as relevant. | NA |

For peer review only

| | | | |
|---|---|---|---|
| 26a | Who will obtain informed consent or assent from potential trial participants or authorised surrogates, and how (see Item 32) | See Site and HH recruitment | Pg 8 |
| 26b | Additional consent provisions for collection and use of participant data and biological specimens in ancillary studies, if applicable | See attached Consent and participation forms | See attached Consent and participation forms |
| 27 | How personal information about potential and enrolled participants will be collected, shared, and maintained in order to protect confidentiality before, during, and after the trial | See attached Consent and participation forms | See attached Consent and participation forms |
| 28 | Financial and other competing interests for principal investigators for the overall trial and each study site | See competing interests = none | Page 21 |
| 29 | Statement of who will have access to the final trial dataset, and disclosure of contractual agreements that limit such access for investigators | See Dissemination | Page 15 |
| 30 | Provisions, if any, for ancillary and post-trial care, and for compensation to those who suffer harm from trial participation | Not applicable as the intervention is being implemented at the community level | NA |
| 31a | Plans for investigators and sponsor to communicate trial results to participants, healthcare professionals, the public, and other relevant groups (eg, via publication, reporting in results databases, or other data sharing arrangements), including any publication restrictions | The data collected in the study will be publicly distributed along with metadata and critical documents (i.e, protocols and questionnaires) following the publication of the primary results from the trials. | In Ethics and Dissemination Section (Page 15) |
| 31b | Authorship eligibility guidelines and any intended use of professional writers | See Author's contribution | Page 21 |
| 31c | Plans, if any, for granting public access to the full protocol, participant-level dataset, and statistical code | See Ethics and Dissemination Section | Page 15 |
| 32 | Model consent form and other related documentation given to participants and authorised surrogates | See attached Consent and participation forms | See attached Consent and participation forms 1 |
| 33 | Plans for collection, laboratory evaluation, and storage of biological specimens for genetic or molecular analysis in the current trial and for future use in ancillary studies, if applicable | We will use various molecular approaches to assess the presence and concentration of pathogens, microbial communities, and AMR genes in human faecal samples p See Primary Outcomes<br>See attached Consent and participation forms | Page 11, 12<br>Appendix 2– child consent + PIC |

For peer review only

BMJ Open

# Study design, rationale and methods of the Revitalising Informal Settlements and their Environments (RISE) study: a cluster randomised controlled trial to evaluate environmental and human health impacts of a water sensitive intervention in informal settlements in Indonesia and Fiji

| | |
|---|---|
| Journal: | *BMJ Open* |
| Manuscript ID | bmjopen-2020-042850.R1 |
| Article Type: | Protocol |
| Date Submitted by the Author: | 06-Nov-2020 |
| Complete List of Authors: | Leder, Karin; Monash University Faculty of Medicine Nursing and Health Sciences, School of Public Health and Preventive Medicine<br>Openshaw, John; Stanford University, Infectious diseases and Geographic Medicine Division<br>Allotey, P; United Nations University International Institute for Global Health<br>Ansariadi , Ansariadi; Hasanuddin University, Public Health Faculty<br>Barker, S Fiona; Monash University Faculty of Medicine Nursing and Health Sciences, School of Public Health and Preventive Medicine<br>Burge, Kerrie; Monash University, CRC for Water Sensitive Cities<br>Clasen, Thomas; Emory University, Department of Environmental Health Sciences, Rollins School of Public Health<br>Chown, Steven; Monash University, School of Biological Sciences<br>Duffy, Grant; Monash University, School of Biological Sciences<br>Faber, Peter; Monash University, School of Biological Sciences<br>Fleming, Genie; Monash University, School of Biological Sciences<br>Forbes, Andrew; Monash University Faculty of Medicine Nursing and Health Sciences, School of Public Health and Preventive Medicine<br>French, Matthew; Monash University, Monash Sustainable Development Institute<br>Greening, Chris; Monash University Faculty of Medicine Nursing and Health Sciences, Department of Microbiology<br>Henry, Rebekah; Monash University, Civil Engineering<br>Higginson, Ellen; University of Cambridge, Cambridge Institute for Therapeutic Immunology and Infectious Disease<br>Johnston, David W.; Monash University, Centre for Health Economics, Monash Business School<br>Lappan, Rachael; Monash University Faculty of Medicine Nursing and Health Sciences, Department of Microbiology<br>Lin, Audrie; University of California Berkeley, School of Public Health<br>Luby, Stephen; Stanford University, Infectious diseases and Geographic Medicine Division<br>McCarthy, David; Monash University, Civil Engineering<br>O'Toole, Joanne; Monash University Faculty of Medicine Nursing and Health Sciences, School of Public Health and Preventive Medicine |

| | |
|---|---|
| | Ramirez-Lovering, Diego; Monash University, Art, Design and Architecture<br>Reidpath, Daniel; Monash University - Malaysia Campus<br>Simpson, Julie; University of Melbourne School of Population and Global Health, Centre for Epidemiology and Biostatistics<br>Sinharoy, Sheela; Emory University, Rollins School of Public Health<br>Sweeney, Rohan; Monash University, Centre for Health Economics, Monash Business School<br>Taruc, Ruzka R; Hasanuddin University, Public Health Faculty<br>Tela, Autiko; Fiji National University College of Medicine Nursing and Health Sciences - Tamavua Campus, School of Public Health & Primary Care<br>Turagabeci, Amelia; Fiji National University College of Medicine Nursing and Health Sciences - Tamavua Campus, School of Public Health & Primary Care<br>Wardani, Jane; Monash University, Monash Sustainable Development Institute<br>Wong, Tony; Monash University, CRC for Water Sensitive Cities<br>Brown, Rebekah ; Monash University, Monash Sustainable Development Institute |
| <b>Primary Subject Heading</b>: | Public health |
| Secondary Subject Heading: | Infectious diseases |
| Keywords: | PUBLIC HEALTH, Gastrointestinal infections < GASTROENTEROLOGY, Epidemiology < INFECTIOUS DISEASES, Public health < INFECTIOUS DISEASES, TROPICAL MEDICINE |
| | |

SCHOLARONE™
Manuscripts

*I, the Submitting Author has the right to grant and does grant on behalf of all authors of the Work (as defined in the below author licence), an exclusive licence and/or a non-exclusive licence for contributions from authors who are: i) UK Crown employees; ii) where BMJ has agreed a CC-BY licence shall apply, and/or iii) in accordance with the terms applicable for US Federal Government officers or employees acting as part of their official duties; on a worldwide, perpetual, irrevocable, royalty-free basis to BMJ Publishing Group Ltd ("BMJ") its licensees and where the relevant Journal is co-owned by BMJ to the co-owners of the Journal, to publish the Work in this journal and any other BMJ products and to exploit all rights, as set out in our licence.*

*The Submitting Author accepts and understands that any supply made under these terms is made by BMJ to the Submitting Author unless you are acting as an employee on behalf of your employer or a postgraduate student of an affiliated institution which is paying any applicable article publishing charge ("APC") for Open Access articles. Where the Submitting Author wishes to make the Work available on an Open Access basis (and intends to pay the relevant APC), the terms of reuse of such Open Access shall be governed by a Creative Commons licence – details of these licences and which Creative Commons licence will apply to this Work are set out in our licence referred to above.*

*Other than as permitted in any relevant BMJ Author's Self Archiving Policies, I confirm this Work has not been accepted for publication elsewhere, is not being considered for publication elsewhere and does not duplicate material already published. I confirm all authors consent to publication of this Work and authorise the granting of this licence.*

For peer review only

# Study design, rationale and methods of the Revitalising Informal Settlements and their Environments (RISE) study: a cluster randomised controlled trial to evaluate environmental and human health impacts of a water sensitive intervention in informal settlements in Indonesia and Fiji

Karin Leder[a], John J Openshaw[b], Pascale Allotey[c], Ansariadi Ansariadi [d], S Fiona Barker[a], Kerrie Burge[e], Thomas Clasen[f], Steven L Chown[g], Grant A Duffy[g], Peter A Faber[g], Genie Fleming[g], Andrew B Forbes[a], Matthew French[h], Chris Greening[i], Rebekah Henry[j], Ellen Higginson[k], David Johnston[l], Rachael Lappan[i] , Audrie Lin[m], Stephen P Luby[b], David McCarthy[j], Joanne E O'Toole[a], Diego Ramirez-Lovering[n], Daniel Reidpath[o], Julie A Simpson[p], Sheela S Sinharoy[f], Rohan Sweeney[l], Ruzka R Taruc[d], Autiko Tela[q], Amelia R Turagabeci[q], Jane Wardani[h], Tony Wong[e], Rebekah Brown[h], and the RISE Consortium

[a] School of Public Health and Preventive Medicine, Monash University, Australia
[b] Infectious diseases and Geographic Medicine Division, Stanford University, USA
[c] International Institute for Global Health, United Nations University, Malaysia
[d] Public Health Faculty, Hasanuddin University, Indonesia
[e] CRC for Water Sensitive Cities, Monash University, Australia
[f] Rollins School of Public Health, Emory University, USA
[g] School of Biological Sciences, Monash University, Australia
[h] Monash Sustainable Development Institute, Monash University, Australia
[i] Department of Microbiology, Monash University, Australia
[j] Civil Engineering, Monash University, Australia
[k] Cambridge Institute for Therapeutic Immunology and Infectious Disease, University of Cambridge
[l] Centre for Health Economics, Monash Business School, Monash University, Australia
[m] School of Public Health, University of California, Berkeley, USA
[n] Art, Design and Architecture, Monash University, Australia
[o] Monash University Malaysia, Bandar Sunway, Malaysia
[p] Centre for Epidemiology and Biostatistics, Melbourne School of Population and Global Health University of Melbourne, Australia
[q] School of Public Health & Primary Care, Fiji National University, Fiji

The RISE consortium: Fitriyanty Awaluddin [d], Becky Batagol[r], Lamiya Bata[j], Dieter Bulach[is], Bruce Cahan[t], Brett Davis[h], Mohammed El-Sioufi[n], Dusan Jovanovic [j], Michaela F Prescott[n], Emma Ramsay [g], Briony Rogers[h,u], Maghfira Saifuddaolah [d], Christelle Schang [j], Chi-Wen Tseng [j], Revoni Vamosi [q], Silivia Vilsoni [q], Isoa Vakarewa[v], Andi Zulkifli [d].

[r] Faculty of Law, Monash University, Australia
[s] Medicine, Dentistry and Health Sciences, Melbourne University, Australia
[t] School of Engineering Department of Management Science & Engineering, Stanford University, USA
[u] School of Social Sciences, Monash University, Australia
[v] Live and Learn, Fiji

Corresponding author: Prof K Leder, email: karin.leder@monash.edu

Key Words: RISE, health, environment, informal settlements, water sensitive, planetary health



For peer review only - http://bmjopen.bmj.com/site/about/guidelines.xhtml

**Abstract**

**Introduction:** Increasing urban populations have led to the growth of informal settlements, with contaminated environments linked to poor human health through a range of inter-linked pathways. Here we describe the design and methods for the Revitalising Informal Settlements and their Environments (RISE) study, a transdisciplinary randomised trial evaluating impacts of an intervention to upgrade urban informal settlements in two Asia-Pacific countries.

**Methods and analysis**: RISE is a cluster randomised controlled trial among twelve settlements in Makassar, Indonesia and twelve in Suva, Fiji. Six settlements in each country have been randomised to receive the intervention at the outset; the remainder will serve as controls and be offered intervention delivery after trial completion. The intervention involves a water sensitive approach, delivering site-specific, modular, decentralised infrastructure primarily aimed at improving health by decreasing exposure to environmental faecal contamination. Consenting households within each informal settlement site have been enrolled, with longitudinal assessment to involve health and wellbeing surveys, and human and environmental sampling. Primary outcomes will be evaluated in children aged under 5 years and include prevalence and diversity of gastrointestinal pathogens, abundance and diversity of antimicrobial resistance (AMR) genes in gastrointestinal microorganisms, and markers of gastrointestinal inflammation. Diverse secondary outcomes include changes in microbial contamination; abundance and diversity of pathogens and AMR genes in environmental samples; impacts on ecological biodiversity and microclimates; mosquito vector abundance; anthropometric assessments, nutrition markers and systemic inflammation in children; caregiver- and self-reported health symptoms and healthcare utilisation; and measures of individual and community psychological, emotional and economic wellbeing. The study aims to provide proof-of-concept evidence to inform policies on upgrading of informal settlements to improve environments and human health and wellbeing.

**Ethics**: Study protocols have been approved by ethics boards at Monash University, Fiji National University and Hasanuddin University. Registration: ANZCTR (Trial ID: ACTRN12618000633280).



For peer review only - http://bmjopen.bmj.com/site/about/guidelines.xhtml

**Strengths and Limitations of this study**

- RISE is a transdisciplinary randomised controlled trial (RCT) assessing a range of human and environmental health outcomes in urban informal settlements in Makassar, Indonesia and Suva, Fiji.
- The intervention involves a participatory co-design process to deliver site-specific, water-sensitive, modular infrastructure to reduce faecal environmental contamination and improve water cycle management.
- Primary health outcomes include objective markers of gastrointestinal health in children under five; secondary outcomes include measures of prevalence and diversity of pathogens and antimicrobial resistance markers in both environmental and human samples, as well as ecological, psychological, social and economic impacts.
- The RCT design enables settlement-level cluster randomisation, but blinding to the intervention is not possible.
- Logistic constraints include the number and size of communities enrolled, which limits external validity of findings.



For peer review only - http://bmjopen.bmj.com/site/about/guidelines.xhtml

## INTRODUCTION

Informal settlements are home to more than a billion people, mostly in rapidly growing urban areas of low- and middle-income countries (LMICs). With predictions suggesting that up to three billion people could be living in urban informal settlements by 2050[1], it is imperative to address vulnerabilities faced by informal settlement communities. Informal settlements are often located in inhospitable parts of cities prone to flooding, residents frequently face insecure land tenure, and communities often have inadequate provision of essential centralised urban services including clean water and appropriate sanitation facilities[2]. The confluence of factors faced by residents of informal settlements living in poor conditions results in exposure to compromised ecological conditions and environmental contamination with pathogens and disease vectors, which leads to deleterious impacts on health and wellbeing[3].

The Sustainable Development Goals (SDGs), a set of aspirational goals and targets for attaining sustainable development by 2030, envision that urban communities will become more inclusive, safe, resilient, and sustainable[4]. Reducing environmental exposure to infectious agents transmitted in human faeces is specifically addressed in the SDGs (SDG 6.1 and 6.2); given the impact of such exposures on the burden of disease, they are also implied as a priority by the health goals. However, conventional engineering solutions for water and sanitation—reticulated water and sewerage systems--often do not reach informal urban settlements[2 5 6], mainly as a result of the economic poverty, legal land tenure issues, and lack of political power among residents in these communities. Low-cost solutions, such as household water treatment and on-site sanitation, have had mixed results in recent trials[7-10]. Interventions attempting to address the adverse conditions must therefore consider the complex exposure pathways that link physical aspects of the environment to human health outcomes, and accordingly must interrupt multi-faceted vulnerabilities.

The Revitalising Informal Settlements and their Environments (RISE) trial will implement an intervention designed to address these challenges. The RISE study (www.rise-program.org)[11] will collaborate with communities to design and implement a decentralised wastewater infrastructure which integrates sustainable, water sensitive technology into buildings and landscapes. The infrastructure will be implemented at dwelling, neighborhood, and precinct scales. The focus of the RISE water sensitive intervention is to reduce direct contact with faecal contaminants among informal settlement residents. Delivery of conventional water and sanitation services, namely toilets and hand basins, is incorporated into the RISE study, but in contrast to traditional water, sanitation and hygiene (WASH) approaches, the intervention extends beyond promotion of improved sanitation, drinking water supplies, and handwashing practices[7-10]. Instead, the RISE study will also include settlement-scale infrastructure delivery to address environmental contamination from inadequate attention to management of faecal waste and poorly maintained septic systems, both of which are likely to contribute to suboptimal health impacts in high-density living settings such as occurs in urban informal settlements. The RISE study will also concurrently address upgrading of physical access within communities to limit residents' exposure to environmental pollutants, improve overall water cycle management, diversify water source supplies, and attend to water drainage and flood management. This holistic, settlement-scale approach addresses the recent call for "transformative WASH" or "WASH-plus" solutions[12 13].

Here we describe the rationale of the RISE intervention and the study design and methods of a cluster randomized controlled trial (RCT) to assess the impact of the intervention on the environment and human health and wellbeing. The cluster RCT is being conducted in Makassar, Indonesia and Suva, Fiji in cooperation with local and national governments.

## HYPOTHESES AND AIMS

The hypothesised sequence of change in the RISE study is that implementing a water sensitive intervention in informal settlements leads to improved physical environments, which in turn results in



For peer review only - http://bmjopen.bmj.com/site/about/guidelines.xhtml

reduced human exposure to pathogenic faecal contamination and flooding hazards, resulting in improved human and ecological health and wellbeing (Figure 1). Specifically, we hypothesise that the intervention will reduce environmental contamination with human faeces. We further hypothesise that this will reduce residents' exposure to gastrointestinal pathogens that cause diarrhoeal disease, poor gastrointestinal function, and nutritional deficits, which particularly impact on young children living in informal settlement conditions, as well as reducing exposure to faecal sources of antimicrobial resistance (AMR) genes. Our hypotheses align with the planetary health approach[14], acknowledging the complex interplay between multiple factors which include health, environmental conditions, urbanisation, water and sanitation management, gender and socio-economic equity, and climate change.

We have three central scientific questions and aims that align with our hypotheses.[1]

***Aim 1: Environment***

We will first assess whether the RISE intervention leads to improved environmental characteristics. Urbanisation alters physical environmental conditions such as air temperature, soil contamination, and water quality, and causes transformations in local biodiversity and changes in ecological communities and their functioning[15-18]. Cities tend to have warmer environments than surrounding areas due to the urban heat island effect[19 20], and in tropical informal settlements, high housing density and inadequate formal solid, greywater and faecal waste processing pathways result in considerable eutrophication and faecal contamination of soil and water[21]. Increased populations of mammal (e.g. rats) and arthropod (e.g. mosquito) vectors are often present[22-24], likely due to a combination of thermal changes, habitat modification, resource concentration, and high human population densities[25-27].

Our aim is to assess the impact of delivery of the water sensitive intervention on the physical, thermal, hydrological and microbiological environments. Changes are expected to make residents less vulnerable to the impacts of flooding, reduce the abundance of vectors, increase the diversity of urban ecological communities and alter the composition of microbial communities. Additionally, by improving human excreta management strategies, we aim to decrease faecal contamination of the environment, thereby reducing exposure to pathogens and AMR genes in water and soil.

***Aim 2: Human health***

Our second aim is to assess whether the intervention leads to improved physical health outcomes as a result of improved environmental conditions, as hypothesized above. Prevailing bio-physical conditions in informal settlements are conducive to the spread of infectious diseases. Ample vector breeding sites and poor preventative practices lead to mosquito-borne diseases[28 29]. Children suffer from diarrhoea and poor nutrition [30 31], with contaminated water and soil acting as a source for transmission of pathogens derived from animal, human, and environmental origins[32 33]. Antimicrobial-resistant infections are also a growing threat to human health globally[34], and inadequate faecal waste management leads to environmental contamination which may promote transmission of multi-drug resistant pathogens between humans and the environment[35 36]. Young children under the age of five living in these suboptimal conditions are at especially high risk for under-nutrition, diarrhoea, and poor growth and development. Further worsening their physical health is the high prevalence of environmental enteric dysfunction, a chronic inflammatory condition of the intestine which is associated with poor absorption of nutrients, growth faltering, and impaired response to live enteric vaccines[37 38].

By designing and delivering decentralized infrastructure appropriate to and scalable within informal settlements, we aim to improve faecal sludge handling and reduce contact with faecal contamination in

[1] In addition to these three scientific questions, the RISE program includes two other research objectives: (1) to develop novel practices for built environment and community co-design, and (2) to develop the financial and policy frameworks for scaling up the intervention.



For peer review only - http://bmjopen.bmj.com/site/about/guidelines.xhtml

order to interrupt the connection between the human faecal stream and the environment. These impacts are expected to decrease children's exposure to gastrointestinal pathogens, as detected by molecular methods in faecal samples, to decrease environmental enteric dysfunction, and to decrease the abundance and diversity of AMR markers detected in children's gastrointestinal tract. Flood mitigation and improved access within communities will further decrease human contact with the overall reservoir of environmental pathogens, and falling vector abundance may decrease mosquito-borne disease.

***Aim 3: Wellbeing***

Our third aim is to determine whether the intervention affects the physical environment in ways which impact on community wellbeing and individual subjective psychological and economic wellbeing. Changes to the physical environment affect inhabitants' lifestyle, mental health, self-image, perceptions of safety, and social cohesion[39-41]. They also impact on water security, local economic activities, financial wellbeing, time allocation for water collection and time available for paid work[42-46].

We aim to change the physical environment to positively impact inhabitants' evaluative and hedonic subjective wellbeing; change how people allocate their time; and improve economic activity by increasing employment opportunities and time spent on income-generating activities. At a community level, we aim to improve residents' perceptions of social cohesion and, motivated through the local co-design approach during intervention development, improve community resilience by enhancing collective efficacy.

***Additional outcomes***

In addition to these three key scientific aims, we will evaluate the evidence on the feasibility and impact of the approach to explore the potential for scaling up water sensitive interventions throughout the Asia-Pacific region through rigorous generation of evidence. We will engage with governments, investors and implementers to determine priorities and economic drivers, and identify barriers to intervention to address the environmental challenges of urban informal settlements.

## STUDY METHODS AND ANALYSIS

### Overview of the study design and timeline

To evaluate the RISE intervention, we are conducting a cluster RCT involving 12 informal settlements in Suva, Fiji, and 12 in Makassar, Indonesia, with clustering at the settlement level. In each country, half of the sites have been randomised as intervention communities and will receive an early upgrade. The remaining communities will serve as controls and will receive only standard, basic hygiene and sanitation messages. Monitoring and assessment will be conducted in all settlements over an approximate five-year timeline which will include baseline data collection, the design and build implementation phase of the project, and 2 years of post-build monitoring (Figure 2). After completion of the study, the intervention will be implemented in the 12 control settlements.

### Study settings

The study is being conducted across two countries where large portions of the population are subject to inadequate water and sanitation infrastructure. Indonesia and Fiji were purposively selected to represent a diversity of characteristics across the Asia-Pacific which might influence intervention effects, including climatic factors, water security, tidal inundations, population density, sanitation practices, and socioeconomic and cultural conditions. Within each country, candidate sites were selected in consultation with local government authorities, intervention funders, research partners, communities, and other organisational stakeholders. Formative work consisted of four phases: a) extensive reconnaissance visits to both cities to understand the range of site conditions and to scope potentially suitable sites, b) early engagement with local and national governments to determine priorities and preferences, and to ascertain tenure status for each site, c) household enumeration surveys to determine approximate



For peer review only - http://bmjopen.bmj.com/site/about/guidelines.xhtml

population size and demographics in each settlement, and d) extensive discussions with community leaders and residents to determine their willingness to participate and priorities.

Makassar, the provincial capital of South Sulawesi, has seen rapid urbanisation and population growth in the last decade, with unplanned city growth resulting in a dramatic rise of informal settlements. The 12 settlements of the RISE project are dispersed across the city and represent a diversity of settlement typologies. While the settlements are located in various flood plains and coastal areas, they are all characterised by dense and precariously built housing, little open space, poor vehicular and pedestrian access, and water stressors. Water bodies and soil are typically contaminated with solid waste derived from animal and human sources, and with domestic black and grey wastewater. Seasonal and other flooding is common, requiring makeshift planking for access. Despite some variation in socio-economic status within and across settlements, sites typically house the most economically vulnerable populations. Households across the settlements predominantly live on small parcels of privately-owned land often with one house, or in some instances two or three houses occupied by extended family groups, with varying degrees of land tenure security.

Similar to the settlement sites in Indonesia, the 12 RISE informal settlements in Suva, Fiji, reflect a range of typologies that exist in Fiji and the broader Pacific context. While the urban morphology varies between settlements, they are typically high-to-medium density clusters with some open space and, in some instances, lush vegetation. Some settlements are prone to significant pluvial and fluvial flooding; housing is of varied quality and construction type but is typically characterised by lightweight structures raised off the ground. Tenure status is characteristic of informal settlements in Fiji and the broader Pacific region with limited private land ownership in the selected communities, instead relying on community leases on state land or informal agreements with traditional land-owning units on native lands.

**Settlement eligibility and selection criteria**

Data gathered on potential sites characterised local water supplies, sanitation systems, and flooding events and risk. Inspections were conducted at potential sites to confirm feasibility and accessibility. Site locations and sizes were informed by the UN-Habitat informal settlement selection approach[47]. Key inclusion criteria for settlements consisted of location in urban areas of Makassar and Suva, suitability for delivering the planned intervention, and community receptiveness and commitment to study participation. Final selection criteria consisted of settlement size, environmental, and construction related factors (**Table 1**). The key individual inclusion criterion is residence in a settlement enrolled in the trial.

| **Table 1: Selection criteria for informal settlements** | |
|---|---|
| Settlement size, location, and demographics | 1. 30-100 houses, ideally all in one consolidated group<br>2. Physically separated from other settlements with clear physical boundaries<br>3. No conditions calling for future permanent relocation (i.e. landslide risk)<br>4. Representing the most vulnerable populations of the city<br>5. Location in vulnerable areas with presumed high risk of water-borne disease<br>6. Presence of at least 5-10 children under 5 years |
| Land tenure | 7. Occupants with secure tenure (ownership of the house and land that it sits on, or permission to occupy) |
| Environmental conditions | 8. Presence of water stressors including flooding, poor drainage, limited sanitation and water supply |
| Construction related | 9. Potential for replication and scaling of designs |



For peer review only - http://bmjopen.bmj.com/site/about/guidelines.xhtml

| | |
|---|---|
| Participation | 10. Settlement leaders and inhabitants consenting for health and environmental assessments and infrastructure modification |

**Site and household recruitment**

Community recruitment occurred after extensive discussion with stakeholders, government, community leaders and householders, and was performed by trained local community field worker teams. The unit of recruitment for assessment of the intervention is at the household level. We have attempted to recruit and enroll all households within the agreed boundaries of each informal settlement, with written informed consent obtained from heads of households. Householders were able to consent to participation in the overall RISE project with or without consenting to additional individual survey and sampling components.

**Description of the intervention**

The water sensitive approach to upgrading implemented in the RISE study integrates urban design and urban water cycle planning and management, focusing on water conservation and reducing environmental pollution by improving the quality of wastewater and stormwater prior to reuse or discharge to the environment. Implementation of water sensitive infrastructure will be site-specific, co-designed with each community, and adapted to the different biophysical and sociocultural conditions in each informal settlement. The intervention includes specific components collectively designed to reduce residents' exposure to faecal contamination, requiring evaluation of critical faecal contamination and community exposure pathways at each site to determine optimal available technology solutions. Delivery of traditional approaches to water and sanitation services such as private toilets and hand basins are incorporated. Importantly, solutions also include installation of broader decentralised water infrastructure options to control and reduce the release of contaminated sewage and household greywater into the environment, improve flood mitigation, lower exposure to polluted environments within settlements through better access options, and provide source water availability through rainwater harvesting and wastewater recycling.

Specific infrastructure options include (Figure 3):

- New integrated 'wetpods': these are comprised of a toilet, hand basin and rainwater tank;
- Toilet connections: pressure sewers and traditional collection and treatment systems (pipes, manholes, septic tanks and drains);
- Wastewater treatment: constructed wastewater treatment wetlands for black water[48-50], and biofilters and biofilter drains for grey water[51-54];
- Stormwater drainage and treatment: this includes resizing, reprofiling and formalising drains, and swales, raingardens and constructed stormwater treatment wetlands and permeable paving[55-58];
- Water supply security: rainwater tanks and collection, connection to municipal water supply, water supply disinfection and protection of existing shallow well water supplies;
- Access roads, utility corridors, raised pathways;
- Flood management: including backflow prevention, minor localised spot-filling, minor terrain modification and flood protection walls.

The water sensitive approach has been shown to successfully address urban water management challenges in an integrated way in high income economies[59-66], and will be adapted for informal settlements in the RISE study. We will initially design and deliver a pilot project in each country to test key water sensitive technologies and evaluate soils and plants best suited to local conditions. Monitoring system performance from the two pilot communities will underpin adaptive design adjustments to full-scale implementation.


For peer review only - http://bmjopen.bmj.com/site/about/guidelines.xhtml

As a socio-technical intervention, infrastructure design and delivery will be adapted to context-specific social and bio-physical characteristics and constraints of each community through a participatory process of co-design. The co-design process will promote engagement and informed decision-making with residents of each settlement, particularly emphasising participation by women, children, elderly, disabled and other groups that may have specific needs. The process is characterised by active community participation to ensure participants' preferences for solutions are heard, including informed choices regarding the physical location of new infrastructure. It brings the international community of researchers and practitioners together with the residents living in the study communities, thereby building understanding and a sense of value of the infrastructure to increase the likelihood of success of long-term operations and maintenance. Communal infrastructure will be delivered by local contractors supervised by the project team. Long-term operation and maintenance responsibilities will be addressed via training and ongoing engagement of community representatives and local authorities. By utilising local construction expertise and engaging and empowering stakeholders at household, community, and city levels, we aim to build knowledge and trust with local communities, institutions, and governments to ensure the infrastructure is built and maintained for sustainable, long term benefit.

**Data collection**

Data collection is conducted by field workers specifically employed for assessment of trial outcomes; this team is separate from the personnel implementing the intervention. Data collection comprises biospecimen sampling at both the settlement and household levels, field data measurements, and household surveys. Specimen collection involves blood and faeces from children under the age of 5 years; environmental soil, water and animal scat samples; and vector trapping, with intended sampling frequency as shown in Table 2. Ecological field data assessment involves continuous passive acoustic monitoring, temperature logging, and flood assessment including the use of satellite imagery. Anthropometric measures are taken annually among children under aged 5 years. Surveys are administered quarterly, with different modules asked of varying-aged participants to assess physical health, healthcare utilisation, psychological wellbeing, financial wellbeing, time-use, and perceptions of social cohesion and collective efficacy. Questions regarding household level data (i.e. household member composition, flooding) are administered preferentially to the adult female head of household and questions regarding children under the age of 15 are preferentially directed to the adult caregiver who has seen them most days in the past 7 days. Questions regarding adult health and wellbeing, financial wellbeing, time-use, and perceptions of social cohesion and collective efficacy are preferentially administered to: (i) primary caregivers where households contain children under 15 years, and (ii) female or (iii) male heads for households with no children. If the preferred respondent is not at home, field teams attempt two re-visits. To maintain participation over the trial period and to show appreciation for the time spent by participants on study-related activities, we will provide small culturally appropriate gifts of nominal value, as recommended by our in-country partners.

**Table 2: Data collection tools and specimens for primary and secondary outcomes**

| | Minimum Frequency* | | | |
|---|---|---|---|---|
| **Primary outcomes** | C | Q | B | A |
| Faeces collection, children < 5 years of age for:<br>• Prevalence, diversity and concentration of bacterial, viral, protozoan, and helminth gastrointestinal pathogens in children < 5 years of age (composite primary outcome)<br>• Concentration of intestinal inflammation markers representing environmental enteropathy in children < 5 years of age | | | X | |



For peer review only - http://bmjopen.bmj.com/site/about/guidelines.xhtml

| | | | | |
|---|---|---|---|---|
| • Diversity and abundance of AMR genes in gastrointestinal microorganisms of children < 5 years of age (composite primary outcome) | | | | |
| **Secondary outcomes** | | | | |
| ***Physical Health Measures*** | | | | |
| Blood collection for haemoglobin and blood inflammatory markers, children < 5 years | | | | X |
| Anthropometry measures (length-for-age, weight-for-age and weight-for-length Z-scores) measure, children < 5 years of age | | | | X |
| ***Surveys: Household and community demographic, health and wellbeing*** | | | | |
| Household members and demographics | | | | X |
| Household member behaviors and exposures | | | X | |
| Access to sanitation, water, and hygiene facilities^ | | | | ^ |
| Caregiver-reported acute symptoms (diarrhoea, respiratory infections and febrile illness), children < 5 years of age | | X | | |
| Healthcare system utilisation, children < 5 years of age | | X | | |
| Self-reported or caregiver-reported acute symptoms, healthcare system utilisation, and subjective general health (5 point rating scale) among adults and children 5-15 years | | | X | |
| Caregiver assessed quality of life (Pediatric Quality of Life Inventory, PedsQL, emotional functioning dimension), children 5-15 years | | | X | |
| Self-reported life satisfaction – multiple domains (10 point rating scale), adults | | | X | |
| Center for Epidemiologic Studies Depression Scale [CES-D-10], adults | | | | X |
| Self-reported major adverse life events, adults | | | X | |
| Time-use surveys, children 5 to 15 years and adults^ | | | | ^ |
| Housing tenure+ and collective efficacy (Likert 5 point scales) | | | | X |
| Indicators of household assets and expenditure^ | | | | ^ |
| Intermittent qualitative surveys and focus groups | NA | | | |
| ***Measures of environmental faecal contamination*** | | | | |
| Soil and water (potable, recreational, wastewater) samples and animal scat samples for chemical and microbiological analyses (including markers of faecal contamination; prevalence, diversity and concentration of pathogens; abundance and diversity of AMR genes) | | X | | |
| ***Ecological measures and biodiversity*** | | | | |
| Indoor and outdoor thermal data logging | X | | | |
| Passive acoustic monitoring | X | | | |
| Aerial and satellite imagery | | | | ^ |
| Flood and rain gauges+ | | | | X |
| Mosquito trapping | | X | | |
| Rodent trapping+ | | | | ^ |
| *C = continuous; Q = quarterly; B = Biannual; A = Annual ^First and final years of study +Indonesia baseline survey only | | | | |

**Public involvement**

Members of enrolled communities are involved in the design and conduct of this trial, including the design and implementation of the intervention as described above.

**OUTCOMES**

***Primary outcomes***



For peer review only - http://bmjopen.bmj.com/site/about/guidelines.xhtml

We define our first primary outcome as the prevalence and diversity of bacterial, viral, protozoan, and helminthic gastrointestinal pathogens in children under 5 years. Additional primary outcomes are i) the abundance and diversity of AMR genes in gastrointestinal microorganisms of these children; and ii) the concentration of intestinal inflammation and permeability biomarkers in stool representing environmental enteric dysfunction.

We will use various molecular approaches to assess the presence and concentration of pathogens, microbial communities, and AMR genes in human faecal samples. As indicated below, the same methodologies will be applied for processing of environmental samples, thereby optimising assessment of the impact of the intervention on both environmental and human health outcomes. Total nucleic acids will be extracted from faecal samples in-country. Pathogen levels in samples will be measured by quantitative PCR (qPCR) using TaqMan Array Cards [67 68] targeting >30 bacterial, viral, protozoan and helminthic pathogens. rRNA gene amplicon sequencing will measure microbial biodiversity. Shotgun metagenomic sequencing will be used to gain information about the functional capabilities of pathogens in each sample, including the classification and enumeration of AMR genes. Additionally, over 80 AMR genes will be directly detected by qPCR. Expected environmental enteric dysfunction serological markers include faecal myeloperoxidase[69], faecal α-1-antitrypsin[70], and faecal neopterin[71].

### ***Secondary outcomes***

*Human health secondary outcomes*

Secondary health outcomes include caregiver- and self-reported acute symptoms, healthcare utilisation, inflammatory markers in blood, haemoglobin levels, and anthropometry measures. Caregiver- and self-reported symptoms will be collected via quarterly standardised questionnaires throughout the trial, including the presence of diarrhoea, respiratory and undifferentiated febrile illness over the 7 days prior to the survey visit[72]. Symptoms will be assessed quarterly in children under the age of 5 and every 6 months in children between 5-15 years of age and adults. In all cases, diarrhoea is defined as ≥ 3 loose or watery stools in a 24-hour period. Healthcare utilisation (visitation to healthcare provider, hospitalisation, antibiotic use) by children under 5 years of age within the three months prior to the visit will be surveyed quarterly; for children 5 -15 years of age and adults surveys will be 6-monthly.

Blood from children under 5 years of age will be taken annually by venipuncture. Haemoglobin levels will be measured in the field using a point-of-care device. Plasma and whole blood will be stored for further analyses which will include markers of nutrition and inflammation. To obtain length-for-age, weight-for-age and weight-for-length z-scores in children under 5 years of age, trained staff will follow standard procedures for anthropometric measurements[73]. Pairs of trained anthropometrists will measure standing (in children between 2 and 5 years of age) or recumbent (less than 2 years of age) length (accurate to 0.1 cm) and weight without clothing (accurate to 0.1 kg) in triplicate. In both cases, the median of the three measurements will be used in the analysis[74].

*Community and individual wellbeing secondary outcomes*

Secondary outcomes related to individual and community wellbeing include self-reported general and emotional wellbeing, time-use, measures of collective efficacy and water security, and indicators of socioeconomic status. We will assess the subjective general health and emotional wellbeing of children aged 5-15 years using the (parent-proxy) Paediatric Quality of Life InventoryTM 4.0 Emotional Functioning Scale (PedsQL)[75]. Subjective general health, life satisfaction and major life events (i.e., family death, serious illness, marriage break-up, bankruptcy, victim of crime) of adults will also be collected 6-monthly[76 77]. Child (age 5-15 years) and adult time-use (e.g. minutes/hours spent on different activities in past week) will also be collected, and gender aspects examined. We will assess collective efficacy to monitor changes in community wellbeing, trust, and feelings of safety[78]. Indicators



For peer review only - http://bmjopen.bmj.com/site/about/guidelines.xhtml

of socioeconomic status (i.e., education levels, house building materials, assets owned, bank account, primary activity, time spent working and subjective financial wellbeing) will also be collected to facilitate distributional analyses of any observed impacts. We will also estimate the costs of the intervention and report on cost-effectiveness and costs-benefits.

*Ecological and environmental secondary outcomes*

Secondary outcomes for environmental effects include quarterly chemical and microbiological measures to assess faecal contamination of both soil and water (municipal supply, recreational and wastewater), quarterly measures of vector abundance, and continuous measures of the thermal environment and overall biodiversity. We will compare faecal contamination in water and soil samples across intervention and control settlements. Sample collection and transport will be performed using previously described and optimised methods[79 80] and processed for faecal bacteria quantification[81]. Nucleic acid extraction will be performed in-country via methods that enable simultaneous detection of viruses, bacteria, protozoa, and helminths from environmental samples, as confirmed through optimisation experiments already performed. As with human faeces, pathogen presence and concentrations in samples will be measured using TaqMan Array Cards ([67 68] and AMR markers will be detected by qPCR and shotgun metagenomic sequencing. rRNA gene amplicon sequencing will be performed to determine microbial community composition and diversity[82 83]. The amplicon outputs will also be used for microbial source tracking using the SourceTracker tool[84 85]. Changes to the thermal environment will be assessed at the settlement level through remote sensed data supported by local thermal data and compared with changes to the built environment over time[86]. Continuous temperature tracking will use thermal dataloggers (Thermochron iButtons$^{TM}$). Ten randomly selected houses in each settlement will have thermal data loggers installed and a further 5 thermal data loggers will be deployed across the settlement to measure ambient temperature and humidity. Rainfall data will be derived from multiple (where available) local government-maintained weather stations enhanced with tipping bucket rain gauges installed to maximise coverage across the study area.

We will examine mosquito species variation and abundance over time based on quarterly trapping results. Mosquitoes will be sampled using BG Sentinel II traps[87] with 15 traps deployed per settlement, inside or outside randomly selected houses. Trap location will remain consistent throughout the assessment period. Mosquitoes will be identified to species under dissection microscopes using morphological keys[88-90]. Relative abundance and species composition of rodents in communal areas of settlements will also be surveyed in Makassar at baseline using standard traps and a grid-based trapping design to equate trapping effort across sites[91].The influence of season and physical environmental features, including temperature, humidity and rainfall, on the relative abundance of vector species will be examined[92 93]. Continuous passive acoustic monitoring at settlements will provide a measure of overall biodiversity[94]. The acoustic environment will be monitored using one or two (depending on settlement size) SongMeter SM4 bioacoustic recorders per site for human-audible sound frequencies. Additionally, in Indonesia we will deploy a single SongMeter SM4BAT recorder for ultrasonic frequencies emitted by echolocating bats at each site.

***Referral guidelines***

The study will refer participants for treatment to appropriate local healthcare providers in cases of acute malnutrition, anaemia, and intestinal helminth infection. WHO weight for length z-scores will be calculated in the field during anthropometry measurements for children under 5 years of age; children meeting WHO/UNICEF criteria for severe malnutrition (weight for length z-score ≤ 3) will be referred to a local health facility. At times of blood draws, all participating children under age 5 years will have their haemoglobin measured in the field. Children with severe anaemia (haemoglobin < 7.0g/dL) will be



For peer review only - http://bmjopen.bmj.com/site/about/guidelines.xhtml

referred to a local health facility. A subset of faeces samples will be immediately assessed for helminths using the Kato-Katz technique, and individuals with positive results will be referred for treatment.

**Randomisation**

Randomisation was performed separately for each city: March 2019 in Makassar and November 2019 in Suva (Figure 2). Randomisation occurred following settlement recruitment and completion of 12 months of baseline surveys. Settlements (clusters) have been randomly assigned to either intervention or control groups, with equal numbers in each group. Covariate-constrained randomisation was utilised using the cycrand package in Stata V15 to achieve balance between the intervention and control groups on key baseline factors determined *a priori*.

For Makassar, intervention and control groups were first balanced on number of children aged under 5 years (4 settlements in each group: ≤10, 11-30 and >30 children) and flood risk (high and low, 6 settlements in each), producing 90 allocations. Subsequently, the 30 allocations most imbalanced on average asset score were removed, with the score determined from each household's ownership of selected assets. For Suva, intervention and control groups were first balanced on number of children aged under 5 years (<40 children, 8 settlements; >40 children, 4 settlements) and flood risk and site contamination grouping (2 extreme risk, 2 high risk, and 8 medium risk settlements). This resulted in 120 allocations, from which the 30 most imbalanced allocations of asset score were removed, followed by the 30 most imbalanced allocations of average number of children under 5 years. For each of the cities, this resulted in the 60 best balanced allocations being used for the randomisation (total 3600 [60 × 60] possible allocations for the trial). A randomisation ceremony was held in both cities, where a child from the pilot community selected a ping pong ball from a large glass jar that contained 60 ping pong balls numbered 1 to 60, representing the 60 determined random allocations that achieve the balance criteria specified above.

**Sample size**

The choice of 12 settlements per city, each of ~55 dwellings with 5–6 people per dwelling, has been made to balance the need for statistical power with the constraints of construction logistics and cost. Statistical power was calculated using formulae for cluster RCTs with repeated assessments of a cohort[95] (and confirmed by numerical simulation using generalised linear mixed models) for the three primary health outcomes assessed in children aged under 5 years: i) prevalence of gastrointestinal pathogens and diversity (number of bacterial, viral and parasitic gastrointestinal pathogens per sample); ii) diversity and abundance of AMR markers; and iii) concentration of intestinal inflammation markers. For measures of diversity and abundance of markers of drug resistance in environmental samples, the power calculations assumed the same intra-cluster correlations as for the health outcomes (see below).

For the health outcomes involving subsampling of an average 30 children under 5 years of age per settlement, there is 80% power to detect, from 6 months post-construction and beyond, a 40% relative reduction in the prevalence of gastrointestinal pathogens (assuming a baseline prevalence of 25%) and a 31% relative reduction in the average count of enteric pathogens per child (assuming a baseline average count of 1.0 and overdispersion factor of 2; with a baseline of 2.0 the relative reduction is 23%), and at 12 months an absolute difference of 0.30 SDs (and 0.34 SDs for environmental assessments) in the average concentration of intestinal inflammation markers and average number and abundance of AMR markers. These calculations assume intra-cluster (i.e. within-settlement) correlations between: two children within the same time period 0.10[7]; two children in different time periods 0.067; and repeated measurements of the same child 0.10. The relative reduction of 30% has been informed by the findings reported in systematic reviews, which have found reductions of 30–50% due to water-quality interventions[96 97].



For peer review only - http://bmjopen.bmj.com/site/about/guidelines.xhtml

### Statistical analyses

Analyses will be by intention to treat. Descriptive statistics will report characteristics of settlements and of individuals by randomized arm. Estimation of the effects of the intervention for each outcome will use generalised estimating equations with an exchangeable correlation structure and robust standard errors clustered at settlement level, scaled with a small-sample degrees-of-freedom adjustment[98]. Data from 6-, 12-, 18- and 24-months follow-up visits will be included in the analysis and the intervention effect estimated for the 6 month follow-up timepoint for prevalence and mean number of gastrointestinal pathogens, and at 12 months follow-up for diversity and abundance of AMR markers and for intestinal inflammation markers. For binary and count outcomes, we will use a logarithmic link to estimate ratios of prevalence and mean counts, respectively; for continuous outcomes, the identity link will be used to estimate differences in means. All models will adjust for the baseline covariates, number of children under 5 years, flood risk category (see Randomisation section) and asset score. To enable temporal adjustment, indicator variables for seasonal patterns (e.g. rainy versus dry), local rainfall data for each site, and an indicator term for any major flood event at a particular settlement in a particular quarter will be included in the models. For households that drop out of the study, we will employ multiple imputation using imputation models that preserve the hierarchical data structure. Additional sensitivity analyses will exclude households who moved into the settlement post-construction. There will be no adjustment to the p-values for the assessment of multiple primary health outcomes, however, all findings will be fully reported and interpreted based on incremental evidence.

We will explore relationships between environmental changes and health outcomes by modelling health outcomes at 12-, 18- and 24-months post-construction, using changes in environmental measures at 6-, 12-, or 18-months post-construction, respectively, as principal covariates. We will estimate these effects using generalised estimating equations with pre-construction health and environmental parameters as baseline covariates aggregated at settlement-level, and will include interactions of environmental changes with the intervention for effect-modification assessment, together with adjustment for potential confounders.

Laboratory technicians conducting sample analyses will be blinded to the intervention assignment throughout the study duration through the use of barcodes for sample identification. Owing to the nature of the intervention, community participants will not be blinded.

### Study oversight

The RISE study has an independent International Scientific Advisory Panel (ISAP) and a Governance Committee, both of which provide oversight and advice including in relation to any unintended effects of the trial intervention or trial conduct. The ISAP committee will advise on ethical issues that arise. The study also has a Data Advisory Panel, comprised of representatives across Monash University who provide strategic advice and operational guidance to assure a holistically-advised research data strategy. Given the nature of this trial, there are no stopping rules. Results of the intervention implementation in the first 12 settlements will be used to inform and optimise rollout in the control settlements following completion of the trial.

### Limitations, justifications and additional analyses

Water and sanitation trials aiming for community-level intervention coverage pose great challenges for trial designers[99]. The relative lack of RCTs of such interventions reflects multiple inherent difficulties, including randomly allocating networked infrastructure, the long follow-up and large sample sizes required to study health outcomes, and the need for a complex engineering and construction phase[13]. The RISE study will deliver site-specific and bespoke interventions to each settlement, and while this is deliberate and a strength of our approach as it will address site-specific needs, it also results in a lack of intervention uniformity across communities. The purposive selection of settlements included and the relatively small



For peer review only - http://bmjopen.bmj.com/site/about/guidelines.xhtml

size of each community compared with many informal settlement settings, while necessary from a logistics perspective, also limits broad external validity. Furthermore, while we intend to monitor the sustainability of the intervention, the RCT is funded to perform formal monitoring for an initial 2 years post intervention delivery.

Complete settlement revitalisation should ideally encompass a broad suite of measures incorporating comprehensive upgrading of solid waste management; improvements in housing conditions, roads, street lighting and other amenities in communal areas; and delivering a range of other services. However, to test our specific hypothesis (and given logistic and financial constraints), our planned site-specific intervention is restricted in scope to addressing many of these issues predominantly as they relate to our primary focus of delivering improved water and sanitation infrastructure and reduced flooding impacts, in order to minimise human exposure to environmental faecal contamination. For example, attention to solid waste management is important to ensure that the wetlands, which treat the wastewater on-site, will function as expected and not become blocked with solid waste, as this would reduce the ability of wetland plants to remove contaminants from the wastewater and potentially become a breeding site for vectors (i.e. mosquitoes).

Studies such as RISE which involve complex infrastructure upgrades face methodological evaluation challenges and cannot be performed in a blinded fashion, thereby potentially introducing biases in survey-based outcomes. For trials aimed at improving gastrointestinal health among children, the reliability of caregiver-reported diarrhoea is highly variable and in unblinded trials is particularly subject to bias[100]. An alternative approach of testing for organisms in human faeces therefore has advantages, but does not reliably distinguish between non-pathogenic carriage versus causal links of enteropathogens to diarrhoea[101] or impaired nutrient absorption and growth[102 103]. Hence, we will monitor a combination of primary objective outcomes as well as secondary clinical outcome biomarkers to assess children's physical health. However, while we have described our planned methodologies for analytical testing, the multi-year timeline of the RISE trial means that analytical methods may improve during the study (for example, methods for measuring environmental enteric dysfunction), and accordingly we may need to make changes to the specific tests mentioned in this protocol. The results of our initial analyses to identify which pathogens and AMR genes are most prevalent in the study areas will inform decisions regarding optimal approaches for detection throughout the study period.

Defining primary versus secondary aims and outcomes from complex multifactorial community upgrades requires somewhat artificial prioritisation of transdisciplinary effects. This reflects that anticipated impacts of multi-dimensional interventions on communities occur along an interconnected causal chain rather than in a simple hierarchical and linear cause-and-effect fashion. While we have pre-specified our primary outcomes to reflect objective markers of gastrointestinal health in children under the age of 5 years, our fundamentally transdisciplinary intervention and assessment protocol appropriately accounts for the complex interconnections between ecosystem health, environmental contamination, vector abundance, physical health and wellbeing. Our intervention has potential to improve liveability through improved access and opportunities for use of community spaces, better economic sustainability through improved micro-economies and urban farming opportunities, and climate resilience through the diversification of water supplies and flood management. We will therefore assess a range of additional secondary, albeit equally important, impacts on environmental and ecological health, as well as on psychological, social and economic wellbeing outcomes, which we will measure at both the individual and community level.

There are significant challenges with both implementing and measuring impacts of infrastructure upgrades, and for determining which components of an intervention contribute most effectively to observed outcomes[104-106]. Co-design interventions and upgrading programs have often failed to include a counterfactual or have taken a quasi-experimental approach[107], and previous studies have



For peer review only - http://bmjopen.bmj.com/site/about/guidelines.xhtml

often used narrowly focused or proxy markers of effect rather than direct outcome measures[104-107]. Our planned pragmatic cluster-RCT approach to rigorously assess intervention effects is a notable strength. However, the RISE program involves a complex intervention implemented into complex dynamic communities. Thus, we anticipate a range of outcomes, including potentially important consequences that we cannot accurately prespecify. While we can be most confident of the design-based inference from prespecified outcomes, we also foresee contributing broadly to literature on the impact of the interventions across a wider array of outcomes beyond those explicitly mentioned in this protocol, with findings interpreted based on direction, magnitude and incremental evidence. Additionally, data and specimen collection tools not mentioned here will be appended to address supplementary questions, thereby enabling examination of additional health, environmental and social assessments in the participating informal settlements.

## ETHICS AND DISSEMINATION

Ethics review and approval was provided by participating universities and local IRBs, including Monash University Human Research Ethics Committee (Melbourne, Australia; protocol 9396), the Ministry of Research, Technology and Higher Education Ethics Committee of Medical Research at the Faculty of Medicine, Universitas Hasanuddin (Makassar, Indonesia; protocol UH18020110), and the College Human Health Research Ethics Committee (CHREC) at the Fiji Institute of Pacific Health Research (FIPHR) and College of Medicine, Nursing, and Health Sciences at Fiji National University (FNU) (Suva, Fiji; protocol 137.19). Animal ethics for rodent trapping has also been secured (Monash University project ID16351; Universitas Hasanuddin UH18080446). The trial is registered on the Australian New Zealand Clinical Trials Registry (ANZCTR) (Trial ID: ACTRN12618000633280). Prior to providing informed consent, all study settlements, households, and caregivers/respondents are given written explanatory statements describing the voluntary nature of study participation and the fact that they can withdraw from any or all of the study components at any time. Verbal consent is affirmed and documented at each subsequent approach prior to proceeding with survey and sample collection. The data collected in the study will be publicly distributed along with metadata and critical documents (i.e, protocols and questionnaires) following the publication of the primary results from the trials.



For peer review only - http://bmjopen.bmj.com/site/about/guidelines.xhtml

## References

1. United Nations. Sustainable Development Goals Report, 2019, New York. Accessed from https://unstats.un.org/sdgs/report/2019/The-Sustainable-Development-Goals-Report-2019.pdf, 13th June 2020.
2. Turley R, Saith R, Bhan N, et al. Slum upgrading strategies involving physical environment and infrastructure interventions and their effects on health and socio-economic outcomes. *Cochrane Database of Systematic Reviews* 2013(1.)
3. Ezeh A, Oyebode O, Satterthwaite D, et al. The history, geography, and sociology of slums and the health problems of people who live in slums. *The Lancet* 2017;389(10068):547-58.
4. United Nations. SDG Indicators, 2020. Accessed from https://unstats.un.org/sdgs/report/2019/Goal-11/, 13th June 2020.
5. Lilford RJ, Oyebode O, Satterthwaite D, et al. Improving the health and welfare of people who live in slums. *The Lancet* 2017;389(10068):559-70.
6. Weimann A, Oni T. A Systematised Review of the Health Impact of Urban Informal Settlements and Implications for Upgrading Interventions in South Africa, a Rapidly Urbanising Middle-Income Country. *International Journal of Environmental Research and Public Health* 2019;16(19):3608.
7. Clasen T, Boisson S, Routray P, et al. Effectiveness of a rural sanitation programme on diarrhoea, soil-transmitted helminth infection, and child malnutrition in Odisha, India: a cluster-randomised trial. *Lancet Glob Health* 2014;2(11):e645-53.
8. Luby SP, Rahman M, Arnold BF, et al. Effects of water quality, sanitation, handwashing, and nutritional interventions on diarrhoea and child growth in rural Bangladesh: a cluster randomised controlled trial. *Lancet Glob Health* 2018;6(3):e302-e15.
9. Null C, Stewart CP, Pickering AJ, et al. Effects of water quality, sanitation, handwashing, and nutritional interventions on diarrhoea and child growth in rural Kenya: a cluster-randomised controlled trial. *Lancet Glob Health* 2018;6(3):e316-e29.
10. Pickering AJ, Djebbari H, Lopez C, et al. Effect of a community-led sanitation intervention on child diarrhoea and child growth in rural Mali: a cluster-randomised controlled trial. *Lancet Glob Health* 2015;3(11):e701-11.
11. Brown R, Leder, K., Wong, T. et al. Improving human and environmental health in urban informal settlements: the Revitalising Informal Settlements and their Environments (RISE) programme *Lancet Planetary Health* 2018;2:S29.
12. Pickering AJ, Null C, Winch PJ, et al. The WASH Benefits and SHINE trials: interpretation of WASH intervention effects on linear growth and diarrhoea. *Lancet Glob Health* 2019;7(8):e1139-e46.
13. Cumming O, Arnold BF, Ban R, et al. The implications of three major new trials for the effect of water, sanitation and hygiene on childhood diarrhea and stunting: a consensus statement. *BMC Med* 2019;17(1):173.
14. Whitmee S, Haines A, Beyrer C, et al. Safeguarding human health in the Anthropocene epoch: report of The Rockefeller Foundation-Lancet Commission on planetary health. *Lancet* 2015;386(10007):1973-2028.
15. Chown SL, Duffy GA. Thermal physiology and urbanization: perspectives on exit, entry and transformation rules. *Functional Ecology* 2015;29(7):902-12.
16. Seto KC, Sánchez-Rodríguez R, Fragkias M. The New Geography of Contemporary Urbanization and the Environment. *Annual Review of Environment and Resources* 2010;35(1):167-94.
17. Elmqvist T, Fragkias M, Goodness J, et al. Urbanization, Biodiversity and Ecosystem Services: Challenges and Opportunities. Dordrecht, Germany: Springer 2013.
18. Seto KC, Güneralp B, Hutyra LR. Global forecasts of urban expansion to 2030 and direct impacts on biodiversity and carbon pools. *PNAS* 2012;109(40):16083-88.
19. Oke TR. The energetic basis of the urban heat island. *Quarterly Journal of the Royal Meteorological Society* 1982;108(455):1-24.
20. Peng S, Piao S, Ciais P, et al. Surface Urban Heat Island Across 419 Global Big Cities. *Environ Sci Technol* 2012;46(2):696-703.
21. Katukiza AY, Ronteltap M, Niwagaba CB, et al. Sustainable sanitation technology options for urban slums. *Biotechnology Advances* 2012;30(5):964-78.



For peer review only - http://bmjopen.bmj.com/site/about/guidelines.xhtml

22. Alirol E, Getaz L, Stoll B, et al. Urbanisation and infectious diseases in a globalised world. *Lancet Infect Dis* 2011;11(2):131-41.
23. Eisenstein M. Disease: Poverty and pathogens. *Nature* 2016;531(7594):S61-63.
24. Himsworth CG, Parsons KL, Jardine C, et al. Rats, cities, people, and pathogens: a systematic review and narrative synthesis of literature regarding the ecology of rat-associated zoonoses in urban centers. *Vector Borne Zoonotic Dis* 2013;13(6):349-59.
25. Franklinos LHV, Jones KE, Redding DW, et al. The effect of global change on mosquito-borne disease. *The Lancet Infectious Diseases* 2019;19(9):e302-e12.
26. Hassell JM, Begon M, Ward MJ, et al. Urbanization and Disease Emergence: Dynamics at the Wildlife–Livestock–Human Interface. *Trends in Ecology & Evolution* 2017;32(1):55-67.
27. Lau CL, Smythe LD, Craig SB, et al. Climate change, flooding, urbanisation and leptospirosis: fuelling the fire? *Trans R Soc Trop Med Hyg* 2010;104(10):631-38.
28. Carvalho S, Magalhães MdAFM, Medronho RdA. Analysis of the spatial distribution of dengue cases in the city of Rio de Janeiro, 2011 and 2012. *Rev Saude Publica* 2017;51:79.
29. Daudé É, Mazumdar S, Solanki V. Widespread fear of dengue transmission but poor practices of dengue prevention: A study in the slums of Delhi, India. *PLoS Med* 2017;12(2):e0171543.
30. Feleke H, Medhin G, Abebe A, et al. Enteric pathogens and associated risk factors among under-five children with and without diarrhea in Wegera District, Northwestern Ethiopia. *Pan Afr Med J* 2018;29:72.
31. Korpe PS, Valencia C, Haque R, et al. Epidemiology and Risk Factors for Cryptosporidiosis in Children From 8 Low-income Sites: Results From the MAL-ED Study. *Clin Infect Dis* 2018;67(11):1660-69.
32. Hagan JE, Moraga P, Costa F, et al. Spatiotemporal Determinants of Urban Leptospirosis Transmission: Four-Year Prospective Cohort Study of Slum Residents in Brazil. *PLoS Negl Trop Dis* 2016;10(1):e0004275.
33. Majorin F, Torondel B, Routray P, et al. Identifying Potential Sources of Exposure Along the Child Feces Management Pathway: A Cross-Sectional Study Among Urban Slums in Odisha, India. *The American Journal of Tropical Medicine and Hygiene* 2017;97(3):861-69.
34. World Health Organisation. Global antimicrobial resistance surveillance system (GLASS) report: Early implementation 2016-2017. Accessed from https://www.who.int/glass/resources/publications/early-implementation-report/en/, June 10th 2020.
35. Pehrsson EC, Tsukayama P, Patel S, et al. Interconnected microbiomes and resistomes in low-income human habitats. *Nature* 2016;533:212-16.
36. Nadimpalli ML, Marks SJ, Montealegre MC, et al. Urban informal settlements as hotspots of antimicrobial resistance and the need to curb environmental transmission. *Nat Microbiol* 2020;5(6):787-95.
37. Keusch GT, Rosenberg IH, Denno DM, et al. Implications of acquired environmental enteric dysfunction for growth and stunting in infants and children living in low- and middle-income countries. *Food Nutr Bull* 2013;34(3):357-64.
38. Naylor C, Lu M, Haque R, et al. Environmental Enteropathy, Oral Vaccine Failure and Growth Faltering in Infants in Bangladesh. *EBioMedicine* 2015;2(11):1759-66.
39. Vlahov D, Freudenberg N, Proietti F, et al. Urban as a Determinant of Health. *J Urban Health* 2007;84(Suppl 1):16-26.
40. Ferguson KT, Cassells RC, MacAllister JW, et al. The physical environment and child development: An international review. *International Journal of Psychology* 2013;48(4):437-68.
41. Guite HF, Clark C, Ackrill G. The impact of the physical and urban environment on mental well-being. *Public Health* 2006;120(12):1117-26.
42. Devoto F, Duflo E, Dupas P, et al. Happiness on Tap: Piped Water Adoption in Urban Morocco. *American Economic Journal: Economic Policy* 2012;4(4):68-99.
43. Evans GW. Child Development and the Physical Environment. *Annual Review of Psychology* 2006;57(1):423-51.
44. Lambert A, Vlaar J, Herrington S, et al. What Is the Relationship between the Neighbourhood Built Environment and Time Spent in Outdoor Play? A Systematic Review. *International Journal of Environmental Research and Public Health* 2019;16(20):3840.

For peer review only



For peer review only - http://bmjopen.bmj.com/site/about/guidelines.xhtml

45. Marx B, Stoker T, Suri T. The Economics of Slums in the Developing World. *Journal of Economic Perspectives* 2013;17(4):187-210.
46. Pieter van Dijk M, Etajak S, Mwalwega B, et al. Financing sanitation and cost recovery in the slums of Dar es Salaam and Kampala. *Habitat International* 2014;43:206-13.
47. Moreno EL. Slums of the world: The face of urban poverty in the new millennium. Nairobi, Kenya: UN-HABITAT, 2003.
48. Kadlac RH, Wallace SD. Treatment Wetlands, 2nd Ed. Florida, USA: CRC Press 2008.
49. Wu S, Carvalho PN, Müller JA, et al. Sanitation in constructed wetlands: A review on the removal of human pathogens and fecal indicators. *Science of The Total Environment* 2016;541:8-22.
50. Zhao Z, Song X, Wang Y, et al. Effects of algal ponds on vertical flow constructed wetlands under different sewage application techniques. *Ecological Engineering* 2016;93:120-28.
51. Bratieres K, Fletcher TD, Deletic A, et al. Nutrient and sediment removal by stormwater biofilters: A large-scale design optimisation study. *Water Research* 2008;42(14):3930-40.
52. Hatt BE, Fletcher TD, Deletic A. Hydraulic and Pollutant Removal Performance of Fine Media Stormwater Filtration Systems. *Environ Sci Technol* 2008;42(7):2535-41.
53. Kim H, Seagren EA, Davis AP. Engineered bioretention for removal of nitrate from stormwater runoff. *Water Environ Res* 2003;75(4):355-67.
54. Read J, Wevill T, Fletcher T, et al. Variation among plant species in pollutant removal from stormwater in biofiltration systems. *Water Research* 2008;42(4):893-902.
55. Davies CM, Bavor HJ. The fate of stormwater-associated bacteria in constructed wetland and water pollution control pond systems. *J Appl Microbiol* 2000;89(2):349-60.
56. García JA, Paredes D, Cubillos JA. Effect of plants and the combination of wetland treatment type systems on pathogen removal in tropical climate conditions. *Ecological Engineering* 2013;58:57-62.
57. Walker DJ, Hurl S. The reduction of heavy metals in a stormwater wetland. *Ecological Engineering* 2002;18(4):407-14.
58. Wong T, Breen PF, Somes NLG, et al. Managing Urban Stormwater using Constructed Wetlands, Industry Report 98/7. Melbourne, Australia: Cooperative Research Centre for Catchment Hydrology, 1998.
59. Austrade. China's Sponge City Program: Making China's cities watertight presents opportunities for Australian water technologies, 2016. Accessed from https://goo.gl/kHUcqh, May 10th 2020.
60. Cooperative Research Centre for Water Sensitive Cities. Kunshan awarded status as a National Ecological Garden City, 2016. Accessed from https://watersensitivecities.org.au/content/kunshan-awarded-status-national-ecological-garden-city/, May 8th 2020.
61. Jewish National Fund. New Bio-filters Purify Surface Runoff Water for Reuse, 2015. Accessed from http://www.kkl-jnf.org/about-kkl-jnf/green-israel-news/march-2015/biofilters-ramle-bat-yam, May 8th 2020.
62. O'Meara S. Why China Wants to Build Something Called Sponge Cities, CityLab, 2015. Accessed from https://www.citylab.com/design/2015/11/why-china-wants-to-build-sponge-cities/417114/, May 6th 2020.
63. Monitoring Singapore's first bioretention system: Rain garden at Balam Estate. In: Proceedings of Water Sensitive Urban Design; Building the Water Sensiitve Community, Melbourne, Australia, 2012.
64. Active, Beautiful and Clean (ABC) Waters Programme: towards sustainable stormwater management in Singapore. In: Proceedings of the 6th International Water Sensitive Urban Design Conference and Hydropolis, Perth, Australia, 2009.
65. Framework for Stormwater Quality Management in Singapore. Proceedings of the 12th International Conference on Urban Drainage, Porto Algere, Brazil, 2011.
66. A Dual-mode Biofilter System: Case study in Kfar Sava, Israel. Proceedings of the 12th International Conference on Urban Drainage, Porto Alegre, Brazil, 2011.
67. Liu J, Gratz J, Amour C, et al. A laboratory-developed TaqMan Array Card for simultaneous detection of 19 enteropathogens. *J Clin Microbiol* 2013;51(2):472-80.
68. Liu J, Platts-Mills JA, Juma J, et al. Use of quantitative molecular diagnostic methods to identify causes of diarrhoea in children: a reanalysis of the GEMS case-control study. *Lancet* 2016;388(10051):1291-301.



For peer review only - http://bmjopen.bmj.com/site/about/guidelines.xhtml

69. Wagner M, Peterson CGB, Ridefelt P, et al. Fecal markers of inflammation used as surrogate markers for treatment outcome in relapsing inflammatory bowel disease. *World J Gastroenterol* 2008;14(36):5584-89.
70. Bernier JJ, Florent C, Desmazures C, et al. Diagnosis of protein-losing enteropathy by gastrointestinal clearance of alpha1-antitrypsin. *Lancet* 1978;2(8093):763-64.
71. Campbell DI, McPhail G, Lunn PG, et al. Intestinal inflammation measured by fecal neopterin in Gambian children with enteropathy: association with growth failure, Giardia lamblia, and intestinal permeability. *J Pediatr Gastroenterol Nutr* 2004;39(2):153-57.
72. Overbey KN, Schwab KJ, Exum NG. Comparison of 1-week and 2-week recall periods for caregiver-reported diarrhoeal illness in children, using nationally representative household surveys. *Int J Epidemiol* 2019;48(4):1228-39.
73. de Onis M, Onyango AW, Van den Broeck J, et al. Measurement and standardization protocols for anthropometry used in the construction of a new international growth reference. *Food Nutr Bull* 2004;25(1 Suppl):S27-36.
74. Ayele B, Aemere A, Gebre T, et al. Reliability of measurements performed by community-drawn anthropometrists from rural Ethiopia. *PLOS ONE* 2012;7(1):e30345.
75. Varni JW, Seid M, Kurtin PS. PedsQL 4.0: reliability and validity of the Pediatric Quality of Life Inventory version 4.0 generic core scales in healthy and patient populations. *Med Care* 2001;39(8):800-12.
76. Krueger AB, Stone AA. Progress in measuring subjective well-being. *Science* 2014;436(42)
77. Organisation for Economic Co-operation and Development. OECD Guidelines on Measuring Subjective Wellbeing. Paris, France: OECD Publishing, 2013.
78. Grootaert C, Narayan D, Woolcock M, et al. Measuring social capital: an integrated questionnaire. Washington DC: World Bank, 2004.
79. Henry R, Schang C, Chandrasena GI, et al. Environmental monitoring of waterborne Campylobacter: evaluation of the Australian standard and a hybrid extraction-free MPN-PCR method. *Front Microbiol* 2015;6:74.
80. Jones NR, Millman C, van der Es M, et al. Novel Sampling Method for Assessing Human-Pathogen Interactions in the Natural Environment Using Boot Socks and Citizen Scientists, with Application to Campylobacter Seasonality. *Appl Environ Microbiol* 2017;83(14):e00162-17.
81. Schang C, Henry R, Kolotelo PA, et al. Evaluation of Techniques for Measuring Microbial Hazards in Bathing Waters: A Comparative Study. *PLOS ONE* 2016;11(5):e0155848.
82. Caporaso JG, Lauber CL, Walters WA, et al. Ultra-high-throughput microbial community analysis on the Illumina HiSeq and MiSeq platforms. *ISME J* 2012;6(8):1621-24.
83. Caporaso JG, Lauber CL, Walters WA, et al. Global patterns of 16S rRNA diversity at a depth of millions of sequences per sample. *Proc Natl Acad Sci USA* 2011;108 Suppl 1:4516-22.
84. Henry R, Schang C, Coutts S, et al. Into the deep: Evaluation of SourceTracker for assessment of faecal contamination of coastal waters. *Water Research* 2016;93:242-53.
85. Knights D, Kuczynski J, Charlson ES, et al. Bayesian community-wide culture-independent microbial source tracking. *Nat Methods* 2011;8(9):761-3.
86. Fu P, Weng Q. A time series analysis of urbanization induced land use and land cover change and its impact on land surface temperature with Landsat imagery. *Remote Sensing of Environment* 2016;175:205-14.
87. Williams CR, Long SA, Webb CE, et al. Aedes aegypti Population Sampling Using BG-Sentinel Traps in North Queensland Australia: Statistical Considerations for Trap Deployment and Sampling Strategy. *J Med Entomol* 2007;44(2):345-50.
88. Huang YM. The mosquitoes of Polynesia with a pictorial key to some species associated with filariasis and/or dengue fever. *Mosquito Systematics* 1977;9(3):289-322.
89. Reuben R, Tewari SC, Hiriyan J, et al. Illustrated keys to species of Culex (Culex) associated with Japanese encephalitis in Southeast Asia (Diptera: Culicidae). *Mosquito systematics* 1994;26(2):75-96.
90. Rueda L. Pictorial keys for the identification of mosquitoes (Diptera: Culicidae) associated with Dengue Virus Transmission. *Zootaxa* 2004;589.
91. Aplin KP, Brown PR, Jacob J, et al. Field methods for rodent studies in Asia and the Pacific. Canberra, Australia: Australian Centre for International Agricultural Research, 2003.



For peer review only - http://bmjopen.bmj.com/site/about/guidelines.xhtml

92. Himsworth CG, Jardine CM, Parsons KL, et al. The Characteristics of Wild Rat (Rattus spp.) Populations from an Inner-City Neighborhood with a Focus on Factors Critical to the Understanding of Rat-Associated Zoonoses. *PLOS ONE* 2014;9(3)
93. Mordecai EA, Caldwell JM, Grossman MK, et al. Thermal biology of mosquito-borne disease. *Ecology Letters* 2019;22(10):1690-708.
94. Bradfer-Lawrence T, Gardner N, Bunnefeld L, et al. Guidelines for the use of acoustic indices in environmental research. *Methods in Ecology and Evolution* 2019;10(10):1796-807.
95. Hooper R, Teerenstra S, de Hoop E, et al. Sample size calculation for stepped wedge and other longitudinal cluster randomised trials. *Stat Med* 2016;35(26):4718-28.
96. Clasen TF, Alexander KT, Sinclair D, et al. Interventions to improve water quality for preventing diarrhoea. *Cochrane Database Syst Rev* 2015(10):CD004794.
97. Ziegelbauer K, Speich B, Mausezahl D, et al. Effect of sanitation on soil-transmitted helminth infection: systematic review and meta-analysis. *PLoS Med* 2012;9(1):e1001162.
98. Mancl LA, DeRouen TA. A covariance estimator for GEE with improved small-sample properties. *Biometrics* 2001;57(1):126-34.
99. Schmidt WP. The elusive effect of water and sanitation on the global burden of disease. *Trop Med Int Health* 2014;19(5):522-7.
100. Luby SP, Agboatwalla M, Hoekstra RM. The variability of childhood diarrhea in Karachi, Pakistan, 2002-2006. *The American Journal of Tropical Medicine and Hygiene* 2011;84(6):870-77.
101. Taniuchi M, Sobuz SU, Begum S, et al. Etiology of diarrhea in Bangladeshi infants in the first year of life analyzed using molecular methods. *J Infect Dis* 2013;208(11):1794-802.
102. Kau AL, Planer JD, Liu J, et al. Functional characterization of IgA-targeted bacterial taxa from undernourished Malawian children that produce diet-dependent enteropathy. *Sci Transl Med* 2015;7(276):276ra24.
103. Smith MI, Yatsunenko T, Manary MJ, et al. Gut microbiomes of Malawian twin pairs discordant for kwashiorkor. *Science* 2013;339(6119):548-54.
104. Corburn J, Sverdlik A. Slum Upgrading and Health Equity. *Int J Environ Res Public Health* 2017;14(4)
105. Field E. KM. Impact Evaluation for Slum Upgrading Interventions. Harvard University: Cambridge, MA, USA, 2006. Accessed from https://scholar.harvard.edu/files/field/files/kremer_field.pdf, 17th October 2020.
106. Jaitman L, Brakarz, J. Evaluation of slum upgrading programs: Literature review and methodological approaches. Technical note No. IDB-TN-604, 2010. Accessed from https://publications.iadb.org/publications/english/document/Evaluation-of-Slum-Upgrading-Programs-Literature-Review-and-Methodological-Approaches.pdf, 17th October 2020.
107. Butala NM, VanRooyen MJ, Patel RB. Improved health outcomes in urban slums through infrastructure upgrading. *Soc Sci Med* 2010;71(5):935-40.



For peer review only - http://bmjopen.bmj.com/site/about/guidelines.xhtml

**Authors' contributions**

Devised the project's main conceptual ideas: KL, PA, TC, SLC, ABF, MF, DJ, DRL, SPL, DM, DR, JAS, TW, RB
Contributed to development of protocol details and implementation: KL, JO, AA, SFB, KB, TC, SLC, GAD, PAF, GF, MF, CG, RH, EH, DJ, RL, AL, SPL, DM, JO'T, DRL, SSS, RS, RT, AT, ART TW, RB
Designed the statistical analysis plan: ABF and JAS
Main drafting of the main manuscript: KL, JO
Critical review and approval of the manuscript: All authors

**Funding:** This trial has been primarily funded by the Wellcome Trust "Our Planet, Our Health" grant [205222/Z/16/Z]. The intervention is funded by the Asian Development Bank, the New Zealand Ministry of Foreign Affairs and Trade, and Monash University. The funders have no role in the study design, data collection and analysis, decision to publish, or preparation of manuscripts.

**Competing interests:** None

**Acknowledgements:** We acknowledge the hard work and dedication of the RISE project field teams in Fiji and Indonesia, and all the study participants. We also thank Dasha Spasjevic for providing Figure 3.

For peer review only



For peer review only - http://bmjopen.bmj.com/site/about/guidelines.xhtml

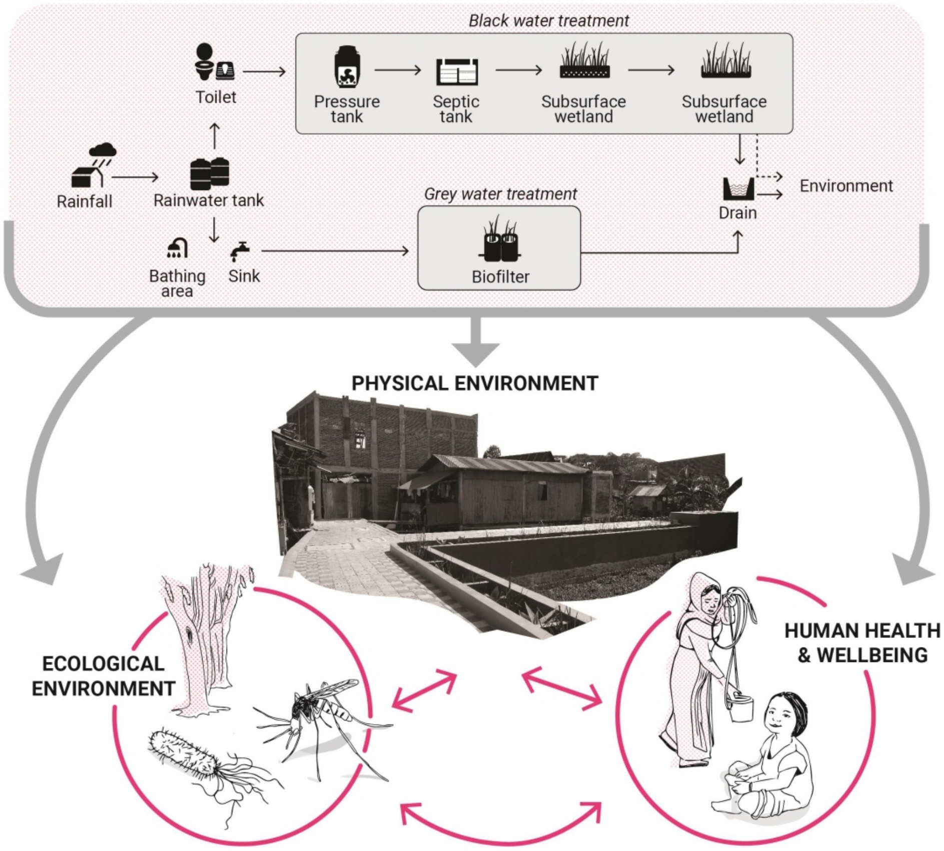


Figure 1: The RISE study hypothesised theory of impacts. The water sensitive intervention will improve the physical environmental conditions, resulting in both direct and indirect impacts on ecology and human physical health and wellbeing

209x191mm (300 x 300 DPI)

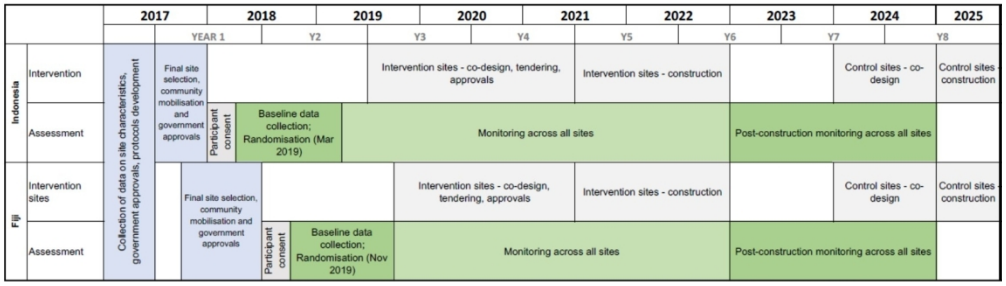


Figure 2: Anticipated RISE study timeline

146x41mm (300 x 300 DPI)

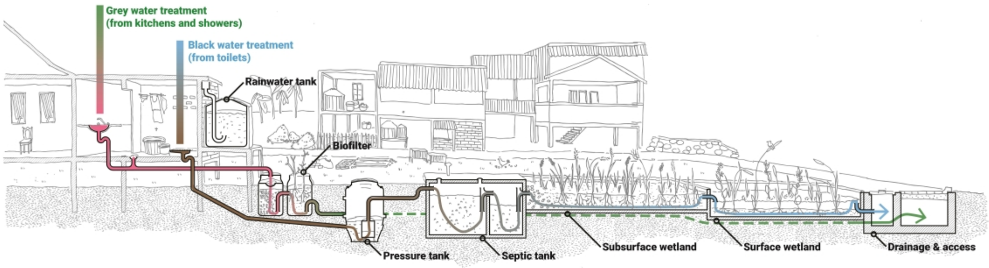


Figure 3: Schematics of proposed sanitation intervention

703x202mm (300 x 300 DPI)

**Checkllist SPIRIT -complete**

| Item no | Description | Information | Found on what Page of the protocol? - current Page numbers |
|---|---|---|---|
| 1 | Descriptive title identifying the study design, population, interventions, and, if applicable, trial acronym | All information in current title: "Study design, rationale and assessment methods of the Revitalising Informal Settlements and their Environments (RISE) study: a cluster randomised controlled trial to evaluate a human health impacts of a water sensitive intervention in informal settlements in Indonesia and Fiji" | Page 1 |
| 2a | Trial identifier and registry name. If not yet registered, name of intended registry | Registration: Australian New Zealand Clinical Trials Registry (ANZCTR) (Trial ID: ACTRN12618000633280). | Abstract (Page 2) and 'Ethics and Dissemination' section (Page 16) |
| 2b | All items from the World Health Organization Trial Registration Data Set | See WHO Trial Registration dataset collection | See WHO Trial Registration dataset collection |
| 3 | Date and version identifier | Article submission will be of version 1 with date of submission identifier | Version 2; 02/11/2020 |
| 4 | Sources and types of financial, material, and other support | See text prepared for end of article: Funding: This trial has been primarily funded by the Wellcome Trust "Our Planet, Our Health" grant 205222/Z/16/Z | See Funding (Page 22) |
| 5a | Names, affiliations, and roles of protocol contributors | | Pages 1 & 22 |
| 5b | Name and contact information for the trial sponsor | Professor Rebekah Brown<br>Senior Vice-Provost & Vice-Provost (Research) and Program Director RISE Revitalising Informal Settlements and their Environments (RISE) Monash University Office of the Provost and Senior Vice-President Chancellery 27 Chancellors Walk Clayton VIC 3800 Australia T: +61 3 9905 2122 E: rebekah.brown@monash.edu | See WHO Trial Registration data set |
| 5c | Role of study sponsor and funders, if any, in study design; collection, management, analysis, and interpretation of data; writing of the report; and the decision to submit the report for publication, including whether they will have ultimate authority over any of these activities | The funders have no role in study design, data collection and analysis, decision to publish, or preparation of manuscripts.' | See 4 above (Page 22) |
| 5d | Composition, roles, and responsibilities of the coordinating centre, steering committee, endpoint adjudication committee, data management team, and other individuals or groups overseeing the trial, if applicable (see Item 21a for data monitoring committee) | The RISE study has an independent International Scientific Advisory Panel (ISAP) and a Governance Committee, both of which provide oversight and advice including in relation to any unintended effects of the trial intervention.<br><br>Specifically, ISAP will provide strategic support and scientific guidance to ensure the highest levels of scientific rigour are adopted and followed. This will ensure that results can stand up to international scrutiny and inform policy and investments. ISAP also provides strategic independent advice on the development and implementation of the | See Page 14 |

Version 2: 2/11/2020

For peer review only

| | | | |
|---|---|---|---|
| | | RISE program, and, where relevant, informs the RISE team of state-of-the-art research methodologies, tools, and best practices. ISAP members will also serve as a DSMB, and will specifically advise on responses to ethical issues or harms if any are detected in the intervention groups. After significant discussion, and given the complex nature of the trial, using a Panel of experts already familiar with the project to serve in this role was deemed a more appropriate and expedient approach than having another separate (traditional DSMB) committee.<br><br>The Governance Advisory Panel serves as an advisory body to the Executive Team, specifically providing independent strategic risk and program management advice.<br><br>The study also has a Data Advisory Panel, comprised of representatives across Monash University who provide strategic advice and operational guidance to assure a holistically-advised research data strategy. Specifically, this panel provides advice on how data are collected, stored and shared. The Panel will strengthen the strategy through promotion of best practices and utilisation of fit-for-purpose research infrastructure that in-turn ensure research data accelerates research activity, whilst also maintaining data protection and privacy in accordance with ethical and regulatory requirements.<br><br>Also see Study oversight section | |
| 6a | Description of research question and justification for undertaking the trial, including summary of relevant studies (published and unpublished) examining benefits and harms for each intervention | This information is included in Introduction, Hypotheses, and Aims | Pages 4, 5, 6 |
| 6b | Explanation for choice of comparators | Yes – clearly explained in Study Methods and Analysis: “RISE is a parallel-cluster RCT involving 12 informal settlements in Suva, Fiji, and 12 in Makassar, Indonesia. In each country, half of the sites have been randomised to receive an early upgrade (‘intervention’ communities), and the remaining will serve as controls”. | Page 6 |
| 7 | Specific objectives or hypotheses | See Hypotheses, and Aims | Pages 4, 5, 6 |
| 8 | Description of trial design including type of trial (eg, parallel group, crossover, factorial, single group), allocation ratio, and framework (eg, superiority, equivalence, noninferiority, exploratory) | Parallel design in title and throughout (superiority trial framework)<br>Statistical analyses section | Page 14 |
| 9 | Description of study settings (eg, community clinic, academic hospital) and list of countries where data will be collected. Reference to where list of study sites can be obtained | See Study settings | Pages 6,7 |



For peer review only - http://bmjopen.bmj.com/site/about/guidelines.xhtml

| | | | |
|---|---|---|---|
| 10 | Inclusion and exclusion criteria for participants. If applicable, eligibility criteria for study centres and individuals who will perform the interventions (eg, surgeons, psychotherapists) | Inclusion and exclusion criteria given in section 'Settlement eligibility and selection criteria" and provided in Table 1 | Pages 7,8 |
| 11a | Interventions for each group with sufficient detail to allow replication, including how and when they will be administered | The intervention is fully described in Section 'Description of the intervention' – including codesign which allows for some variation between sites | Pages 8,9 |
| 11b | Criteria for discontinuing or modifying allocated interventions for a given trial participant (eg, drug dose change in response to harms, participant request, or improving/worsening disease) | Not applicable as the intervention is being implemented at the community level | NA |
| 11c | Strategies to improve adherence to intervention protocols, and any procedures for monitoring adherence (eg, drug tablet return, laboratory tests) | Long-term operation and maintenance responsibilities will be addressed via training and ongoing engagement of community representatives and local authorities. | Page 9 |
| 11d | Relevant concomitant care and interventions that are permitted or prohibited during the trial | Standard hygiene messages will be provided to households in both early- and delayed-intervention communities | Page 6 |
| 12 | Primary, secondary, and other outcomes, including the specific measurement variable (eg, systolic blood pressure), analysis metric (eg, change from baseline, final value, time to event), method of aggregation (eg, median, proportion), and time point for each outcome. Explanation of the clinical relevance of chosen efficacy and harm outcomes is strongly recommended | All primary and secondary outcomes are given under the Outcomes heading on Page 8 and onwards. The specific measurement variables are stated in Table 2<br>We state that presence, abundance and diversity will be compared. We detail the time point for each outcome.<br>We describe median values for height / weight measurements but otherwise method of aggregation in outcomes section; otherwise details in sample size section.<br>Action levels (harm) for Hb and Z score plus helminth given in 'Referral guidelines' Page 13/14 | Table 2 Pages 9- 10<br>Outcomes section, Pages 11, 12<br>Referral guidelines Pages 12,13<br>Sample size Page 13 |
| 13 | Time schedule of enrolment, interventions (including any run-ins and washouts), assessments, and visits for participants. A schematic diagram is highly recommended (see Figure) | Schematic diagram is provided | See Fig 2 |
| 14 | Estimated number of participants needed to achieve study objectives and how it was determined, including clinical and statistical assumptions supporting any sample size calculations | See sample size section | Page 13 |
| 15 | Strategies for achieving adequate participant enrolment to reach target sample size | Community cooperation was obtained prior to enrolment of HH– see section Site and HH recruitment  Page 8. | Page 8 |
| 16a | Method of generating the allocation sequence (eg, computer-generated random numbers), and list of any factors for stratification. To reduce predictability of a random sequence, details of any planned restriction (eg, blocking) should be provided in a separate document that is | See Section 'Randomisation' | Page 13 |



For peer review only - http://bmjopen.bmj.com/site/about/guidelines.xhtml

| | | | |
|---|---|---|---|
| | unavailable to those who enrol participants or assign interventions | | |
| 16b | Mechanism of implementing the allocation sequence (eg, central telephone; sequentially numbered, opaque, sealed envelopes), describing any steps to conceal the sequence until interventions are assigned | See 'Randomisation' section | Page 13 |
| 16c | Who will generate the allocation sequence, who will enrol participants, and who will assign participants to interventions | Statisticians generated a series of allocations of settlements to intervention and control groups satisfying the balance criteria, from which one allocation was randomly chosen by a child from the pilot community in each country (using the tombola method, see details in 'Randomisation section).<br>'Household recruitment was performed by trained local community field worker teams fluent in local languages.' | Page 13<br><br>Page 8 |
| 17a | Who will be blinded after assignment to interventions (eg, trial participants, care providers, outcome assessors, data analysts), and how | Unique, numeric identifiers have been generated for houses, households and participants.<br><br>Samples: Samples collected by field staff are identified with unique barcodes. Barcodes for human samples do not identify the settlement. Therefore, laboratory staff are blinded to the identity of sample collection location. After sample processing, all samples receive a second unique non-identifiable barcode prior to long-term storage at -80C.<br><br>Data: Information about participants (name, date of birth, etc.) is needed by surveyors to conduct household surveys. Paper copies of personal information used by the surveyors is stored in a locked cabinet and will be destroyed when no longer required. Prior to analysis of household survey data, personal details including names, phone numbers and dates of birth is removed, with data instead linked using the unique identifiers. | Page 14 |
| 17b | If blinded, circumstances under which unblinding is permissible, and procedure for revealing a participant's allocated intervention during the trial | Not applicable: no blinding of participants. It is impossible to blind community participants given this is a physical intervention. | Not applicable |
| 18a | Plans for assessment and collection of outcome, baseline, and other trial data, including any related processes to promote data quality (eg, duplicate measurements, training of assessors) and a description of study instruments (eg, questionnaires, laboratory tests) along with their reliability and validity, if known. Reference to where data collection forms can be found, if not in the protocol | Human anthropometrics will be collected as triplicate measurements –<br>Local trained community field are employed<br>Validated Questionnaires to be used | Page 11<br>Page 8<br>Page 11 |
| 18b | Plans to promote participant retention and complete follow-up, | To maintain participation over the trial period small culturally appropriate gifts of | Page 9 |

Version 2: 2/11/2020

| | | | |
|---|---|---|---|
| | including list of any outcome data to be collected for participants who discontinue or deviate from intervention protocols | nominal value – as recommended by our in-country partners – will be provided. | |
| 19 | Plans for data entry, coding, security, and storage, including any related processes to promote data quality (eg, double data entry; range checks for data values). Reference to where details of data management procedures can be found, if not in the protocol | Details available in Ethics application (available on request) | Details available in Ethics application (available on request) |
| 20a | Statistical methods for analysing primary and secondary outcomes. Reference to where other details of the statistical analysis plan can be found, if not in the protocol | See statistical section | Page 14 |
| 20b | Methods for any additional analyses (eg, subgroup and adjusted analyses) | See statistical section | Page 14 |
| 20c | Definition of analysis population relating to protocol non-adherence (eg, as randomised analysis), and any statistical methods to handle missing data (eg, multiple imputation) | Will be analysed as intention to treat | Page 14 |
| 21a | Composition of data monitoring committee (DMC); summary of its role and reporting structure; statement of whether it is independent from the sponsor and competing interests; and reference to where further details about its charter can be found, if not in the protocol. Alternatively, an explanation of why a DMC is not needed | See study oversight section and section #5d above. As noted, members of the International Scientific Advisory Panel will also serve as a DSMB, and will specifically advise on responses to ethical issues or harms if any are detected in the intervention groups. After significant discussion, and given the complex nature of the trial, using a Panel of experts already familiar with the project to serve in this role was deemed a more appropriate and expedient approach than having another separate (traditional DSMB) committee. | Page 14 |
| 21b | Description of any interim analyses and stopping guidelines, including who will have access to these interim results and make the final decision to terminate the trial | See Study Oversight section.<br>Given the nature of this trial, there are no stopping rules. Results of the intervention implementation in the first 12 settlements will be used to inform and optimise rollout in the control settlements following completion of the trial. | Page 14 |
| 22 | Plans for collecting, assessing, reporting, and managing solicited and spontaneously reported adverse events and other unintended effects of trial interventions or trial conduct | See study oversight section<br>"The RISE study has an independent International Scientific Advisory Panel and a Governance Committee, both of which provide oversight and advice, including in relation to any unintended effects of the trial intervention or trial conduct. | Page 14 |
| 23 | Frequency and procedures for auditing trial conduct, if any, and whether the process will be independent from investigators and the sponsor | See study oversight section | Page 14 |
| 24 | Plans for seeking research ethics committee/institutional review board (REC/IRB) approval | See 'Ethics and dissemination section' | Page 16 |
| 25 | Plans for communicating important protocol modifications (eg, changes to eligibility criteria, outcomes, | Trial registries, investigators and human research ethics committees are notified of changes, as relevant. | NA |

Version 2: 2/11/2020

For peer review only - http://bmjopen.bmj.com/site/about/guidelines.xhtml

| | | | |
|---|---|---|---|
| | analyses) to relevant parties (eg, investigators, REC/IRBs, trial participants, trial registries, journals, regulators) | | |
| 26a | Who will obtain informed consent or assent from potential trial participants or authorised surrogates, and how (see Item 32) | See Site and HH recruitment | Page 8 |
| 26b | Additional consent provisions for collection and use of participant data and biological specimens in ancillary studies, if applicable | Documented in Consent and participation forms | Available on request |
| 27 | How personal information about potential and enrolled participants will be collected, shared, and maintained in order to protect confidentiality before, during, and after the trial | Documented in Consent and participation forms.<br><br>Surveyors (community field workers) are independently employed as project staff, and none are from our study sites or from neighbouring settlements. They are all required to sign confidentiality agreements and undergo extensive training in objective survey and sample collection. This training is repeated before each household survey round.<br><br>No identifiable data will be published. | Available on request |
| 28 | Financial and other competing interests for principal investigators for the overall trial and each study site | See competing interests = none | Page 22 |
| 29 | Statement of who will have access to the final trial dataset, and disclosure of contractual agreements that limit such access for investigators | See Dissemination | Page 16 |
| 30 | Provisions, if any, for ancillary and post-trial care, and for compensation to those who suffer harm from trial participation | Not applicable as the intervention is being implemented at the community level | NA |
| 31a | Plans for investigators and sponsor to communicate trial results to participants, healthcare professionals, the public, and other relevant groups (eg, via publication, reporting in results databases, or other data sharing arrangements), including any publication restrictions | The data collected in the study will be publicly distributed along with metadata and critical documents (i.e, protocols and questionnaires) following the publication of the primary results from the trials.<br><br>We are engaging with local communities, participants and other stakeholders throughout the project in a number of ways).<br>• Participant feedback:<br>- Community feedback on preliminary findings is provided through tailored mechanisms, including (i) annual meetings of the Community Engagement Councils (e.g. presenting baseline findings at randomisation events), (ii) household information sheets; (iii) quarterly project newsletters explaining upcoming campaigns, changes to the program, and findings to date; and (iv) Community Engagement Committee meetings where our community field workers communicate the status of | In Ethics and Dissemination Section (Page 16) |

Version 2: 2/11/2020

| | | | |
|---|---|---|---|
| | | research and any significant findings since the last meeting.<br>- Health findings: abnormal Kato Katz, haemoglobin and anthropometric results are provided in written form to households, with support (including a referral letter and transportation costs) to seek care at local health facilities.<br>• Government stakeholders: Preliminary findings and results are presented at the Country-level Technical Coordination Committees, which meet 6-monthly and involve all local partners, relevant Ministries and local authorities. This incorporates feedback on sampling campaigns, response rates, and outcomes form project activities. Reports required for the Asian Development Bank (ADB), such as the ADB Knowledge Products, have also served as a vehicle for communicating preliminary outcomes from the intervention design process.<br>• Global - Feedback on emerging findings and research highlights through monthly newsletters, blog posts (on our website), and the usual additional mechanisms such as conference presentations, journal articles, etc. | |
| 31b | Authorship eligibility guidelines and any intended use of professional writers | See Author's contribution | Page 22 |
| 31c | Plans, if any, for granting public access to the full protocol, participant-level dataset, and statistical code | See Ethics and Dissemination Section | Page 16 |
| 32 | Model consent form and other related documentation given to participants and authorised surrogates | Documented in Consent and participation forms | Available on request |
| 33 | Plans for collection, laboratory evaluation, and storage of biological specimens for genetic or molecular analysis in the current trial and for future use in ancillary studies, if applicable | We will use various molecular approaches to assess the presence and concentration of pathogens, microbial communities, and AMR genes in human faecal samples p See Primary Outcomes<br>Documented in Consent and participation forms | Pages 11, 12<br><br>Available on request |

Version 2: 2/11/2020

BMJ Open

# Study design, rationale and methods of the Revitalising Informal Settlements and their Environments (RISE) study: a cluster randomised controlled trial to evaluate environmental and human health impacts of a water sensitive intervention in informal settlements in Indonesia and Fiji



| | |
|---|---|
| Journal: | *BMJ Open* |
| Manuscript ID | bmjopen-2020-042850.R2 |
| Article Type: | Protocol |
| Date Submitted by the Author: | 25-Nov-2020 |




| | |
|---|---|
| Complete List of Authors: | Leder, Karin; Monash University Faculty of Medicine Nursing and Health Sciences, School of Public Health and Preventive Medicine<br>Openshaw, John; Stanford University, Infectious diseases and Geographic Medicine Division<br>Allotey, P; United Nations University International Institute for Global Health<br>Ansariadi , Ansariadi; Hasanuddin University, Public Health Faculty<br>Barker, S Fiona; Monash University Faculty of Medicine Nursing and Health Sciences, School of Public Health and Preventive Medicine<br>Burge, Kerrie; Monash University, CRC for Water Sensitive Cities<br>Clasen, Thomas; Emory University, Department of Environmental Health Sciences, Rollins School of Public Health<br>Chown, Steven; Monash University, School of Biological Sciences<br>Duffy, Grant; Monash University, School of Biological Sciences<br>Faber, Peter; Monash University, School of Biological Sciences<br>Fleming, Genie; Monash University, School of Biological Sciences<br>Forbes, Andrew; Monash University Faculty of Medicine Nursing and Health Sciences, School of Public Health and Preventive Medicine<br>French, Matthew; Monash University, Monash Sustainable Development Institute<br>Greening, Chris; Monash University Faculty of Medicine Nursing and Health Sciences, Department of Microbiology<br>Henry, Rebekah; Monash University, Civil Engineering<br>Higginson, Ellen; University of Cambridge, Cambridge Institute for Therapeutic Immunology and Infectious Disease<br>Johnston, David W.; Monash University, Centre for Health Economics, Monash Business School<br>Lappan, Rachael; Monash University Faculty of Medicine Nursing and Health Sciences, Department of Microbiology<br>Lin, Audrie; University of California Berkeley, School of Public Health<br>Luby, Stephen; Stanford University, Infectious diseases and Geographic Medicine Division<br>McCarthy, David; Monash University, Civil Engineering<br>O'Toole, Joanne; Monash University Faculty of Medicine Nursing and Health Sciences, School of Public Health and Preventive Medicine |

Ramirez-Lovering, Diego; Monash University, Art, Design and Architecture
Reidpath, Daniel; Monash University - Malaysia Campus
Simpson, Julie; University of Melbourne School of Population and Global Health, Centre for Epidemiology and Biostatistics
Sinharoy, Sheela; Emory University, Rollins School of Public Health
Sweeney, Rohan; Monash University, Centre for Health Economics, Monash Business School
Taruc, Ruzka R; Hasanuddin University, Public Health Faculty
Tela, Autiko; Fiji National University College of Medicine Nursing and Health Sciences - Tamavua Campus, School of Public Health & Primary Care
Turagabeci, Amelia; Fiji National University College of Medicine Nursing and Health Sciences - Tamavua Campus, School of Public Health & Primary Care
Wardani, Jane; Monash University, Monash Sustainable Development Institute
Wong, Tony; Monash University, CRC for Water Sensitive Cities
Brown, Rebekah ; Monash University, Monash Sustainable Development Institute

| | |
|---|---|
| <b>Primary Subject Heading</b>: | Public health |
| Secondary Subject Heading: | Infectious diseases |
| Keywords: | PUBLIC HEALTH, Gastrointestinal infections < GASTROENTEROLOGY, Epidemiology < INFECTIOUS DISEASES, Public health < INFECTIOUS DISEASES, TROPICAL MEDICINE |

SCHOLARONE™
Manuscripts

*I, the Submitting Author has the right to grant and does grant on behalf of all authors of the Work (as defined in the below author licence), an exclusive licence and/or a non-exclusive licence for contributions from authors who are: i) UK Crown employees; ii) where BMJ has agreed a CC-BY licence shall apply, and/or iii) in accordance with the terms applicable for US Federal Government officers or employees acting as part of their official duties; on a worldwide, perpetual, irrevocable, royalty-free basis to BMJ Publishing Group Ltd ("BMJ") its licensees and where the relevant Journal is co-owned by BMJ to the co-owners of the Journal, to publish the Work in this journal and any other BMJ products and to exploit all rights, as set out in our licence.*

*The Submitting Author accepts and understands that any supply made under these terms is made by BMJ to the Submitting Author unless you are acting as an employee on behalf of your employer or a postgraduate student of an affiliated institution which is paying any applicable article publishing charge ("APC") for Open Access articles. Where the Submitting Author wishes to make the Work available on an Open Access basis (and intends to pay the relevant APC), the terms of reuse of such Open Access shall be governed by a Creative Commons licence – details of these licences and which Creative Commons licence will apply to this Work are set out in our licence referred to above.*

*Other than as permitted in any relevant BMJ Author's Self Archiving Policies, I confirm this Work has not been accepted for publication elsewhere, is not being considered for publication elsewhere and does not duplicate material already published. I confirm all authors consent to publication of this Work and authorise the granting of this licence.*

For peer review only

# Study design, rationale and methods of the Revitalising Informal Settlements and their Environments (RISE) study: a cluster randomised controlled trial to evaluate environmental and human health impacts of a water sensitive intervention in informal settlements in Indonesia and Fiji

Karin Leder[a], John J Openshaw[b], Pascale Allotey[c], Ansariadi Ansariadi [d], S Fiona Barker[a], Kerrie Burge[e], Thomas Clasen[f], Steven L Chown[g], Grant A Duffy[g], Peter A Faber[g], Genie Fleming[g], Andrew B Forbes[a], Matthew French[h], Chris Greening[i], Rebekah Henry[j], Ellen Higginson[k], David Johnston[l], Rachael Lappan[i] , Audrie Lin[m], Stephen P Luby[b], David McCarthy[j], Joanne E O'Toole[a], Diego Ramirez-Lovering[n], Daniel Reidpath[o], Julie A Simpson[p], Sheela S Sinharoy[f], Rohan Sweeney[l], Ruzka R Taruc[d], Autiko Tela[q], Amelia R Turagabeci[q], Jane Wardani[h], Tony Wong[e], Rebekah Brown[h], and the RISE Consortium

[a] School of Public Health and Preventive Medicine, Monash University, Australia
[b] Infectious diseases and Geographic Medicine Division, Stanford University, USA
[c] International Institute for Global Health, United Nations University, Malaysia
[d] Public Health Faculty, Hasanuddin University, Indonesia
[e] CRC for Water Sensitive Cities, Monash University, Australia
[f] Rollins School of Public Health, Emory University, USA
[g] School of Biological Sciences, Monash University, Australia
[h] Monash Sustainable Development Institute, Monash University, Australia
[i] Department of Microbiology, Monash University, Australia
[j] Civil Engineering, Monash University, Australia
[k] Cambridge Institute for Therapeutic Immunology and Infectious Disease, University of Cambridge
[l] Centre for Health Economics, Monash Business School, Monash University, Australia
[m] School of Public Health, University of California, Berkeley, USA
[n] Art, Design and Architecture, Monash University, Australia
[o] Monash University Malaysia, Bandar Sunway, Malaysia
[p] Centre for Epidemiology and Biostatistics, Melbourne School of Population and Global Health University of Melbourne, Australia
[q] School of Public Health & Primary Care, Fiji National University, Fiji

The RISE consortium: Fitriyanty Awaluddin [d], Becky Batagol[r], Lamiya Bata[j], Dieter Bulach[is], Bruce Cahan[t], Brett Davis[h], Mohammed El-Sioufi[n], Dusan Jovanovic [j], Michaela F Prescott[n], Emma Ramsay [g], Briony Rogers[h,u], Maghfira Saifuddaolah [d], Christelle Schang [j], Chi-Wen Tseng [j], Revoni Vamosi [q], Silivia Vilsoni [q], Isoa Vakarewa[v], Andi Zulkifli [d].

[r] Faculty of Law, Monash University, Australia
[s] Medicine, Dentistry and Health Sciences, Melbourne University, Australia
[t] School of Engineering Department of Management Science & Engineering, Stanford University, USA
[u] School of Social Sciences, Monash University, Australia
[v] Live and Learn, Fiji

Corresponding author: Prof K Leder, email: karin.leder@monash.edu

Key Words: RISE, health, environment, informal settlements, water sensitive, planetary health



For peer review only - http://bmjopen.bmj.com/site/about/guidelines.xhtml

**Abstract**

**Introduction:** Increasing urban populations have led to the growth of informal settlements, with contaminated environments linked to poor human health through a range of inter-linked pathways. Here we describe the design and methods for the Revitalising Informal Settlements and their Environments (RISE) study, a transdisciplinary randomised trial evaluating impacts of an intervention to upgrade urban informal settlements in two Asia-Pacific countries.

**Methods and analysis**: RISE is a cluster randomised controlled trial among twelve settlements in Makassar, Indonesia and twelve in Suva, Fiji. Six settlements in each country have been randomised to receive the intervention at the outset; the remainder will serve as controls and be offered intervention delivery after trial completion. The intervention involves a water sensitive approach, delivering site-specific, modular, decentralised infrastructure primarily aimed at improving health by decreasing exposure to environmental faecal contamination. Consenting households within each informal settlement site have been enrolled, with longitudinal assessment to involve health and wellbeing surveys, and human and environmental sampling. Primary outcomes will be evaluated in children aged under 5 years and include prevalence and diversity of gastrointestinal pathogens, abundance and diversity of antimicrobial resistance (AMR) genes in gastrointestinal microorganisms, and markers of gastrointestinal inflammation. Diverse secondary outcomes include changes in microbial contamination; abundance and diversity of pathogens and AMR genes in environmental samples; impacts on ecological biodiversity and microclimates; mosquito vector abundance; anthropometric assessments, nutrition markers and systemic inflammation in children; caregiver- and self-reported health symptoms and healthcare utilisation; and measures of individual and community psychological, emotional and economic wellbeing. The study aims to provide proof-of-concept evidence to inform policies on upgrading of informal settlements to improve environments and human health and wellbeing.

**Ethics**: Study protocols have been approved by ethics boards at Monash University, Fiji National University and Hasanuddin University. Registration: ANZCTR (Trial ID: ACTRN12618000633280).

For peer review only



For peer review only - http://bmjopen.bmj.com/site/about/guidelines.xhtml

**Strengths and Limitations of this study**

- RISE is a transdisciplinary randomised controlled trial (RCT) assessing a range of human and environmental health outcomes in urban informal settlements in Makassar, Indonesia and Suva, Fiji.
- The intervention involves a participatory co-design process to deliver site-specific, water-sensitive, modular infrastructure to reduce faecal environmental contamination and improve water cycle management.
- Primary health outcomes include objective markers of gastrointestinal health in children under five; secondary outcomes include measures of prevalence and diversity of pathogens and antimicrobial resistance markers in both environmental and human samples, as well as ecological, psychological, social and economic impacts.
- The RCT design enables settlement-level cluster randomisation, but blinding to the intervention is not possible.
- Logistic constraints include the number and size of communities enrolled, which limits external validity of findings.

For peer review only



For peer review only - http://bmjopen.bmj.com/site/about/guidelines.xhtml

## INTRODUCTION

Informal settlements are home to more than a billion people, mostly in rapidly growing urban areas of low- and middle-income countries (LMICs). With predictions suggesting that up to three billion people could be living in urban informal settlements by 2050[1], it is imperative to address vulnerabilities faced by informal settlement communities. Informal settlements are often located in inhospitable parts of cities prone to flooding, residents frequently face insecure land tenure, and communities often have inadequate provision of essential centralised urban services including clean water and appropriate sanitation facilities[2]. The confluence of factors faced by residents of informal settlements living in poor conditions results in exposure to compromised ecological conditions and environmental contamination with pathogens and disease vectors, which leads to deleterious impacts on health and wellbeing[3].

The Sustainable Development Goals (SDGs), a set of aspirational goals and targets for attaining sustainable development by 2030, envision that urban communities will become more inclusive, safe, resilient, and sustainable[4]. Reducing environmental exposure to infectious agents transmitted in human faeces is specifically addressed in the SDGs (SDG 6.1 and 6.2); given the impact of such exposures on the burden of disease, they are also implied as a priority by the health goals. However, conventional engineering solutions for water and sanitation—reticulated water and sewerage systems--often do not reach informal urban settlements[2 5 6], mainly as a result of the economic poverty, legal land tenure issues, and lack of political power among residents in these communities. Low-cost solutions, such as household water treatment and on-site sanitation, have had mixed results in recent trials[7-10]. Interventions attempting to address the adverse conditions must therefore consider the complex exposure pathways that link physical aspects of the environment to human health outcomes, and accordingly must interrupt multi-faceted vulnerabilities.

The Revitalising Informal Settlements and their Environments (RISE) trial will implement an intervention designed to address these challenges. The RISE study (www.rise-program.org)[11] will collaborate with communities to design and implement a decentralised wastewater infrastructure which integrates sustainable, water sensitive technology into buildings and landscapes. The infrastructure will be implemented at dwelling, neighborhood, and precinct scales. The focus of the RISE water sensitive intervention is to reduce direct contact with faecal contaminants among informal settlement residents. Delivery of conventional water and sanitation services, namely toilets and hand basins, is incorporated into the RISE study, but in contrast to traditional water, sanitation and hygiene (WASH) approaches, the intervention extends beyond promotion of improved sanitation, drinking water supplies, and handwashing practices[7-10]. Instead, the RISE study will also include settlement-scale infrastructure delivery to address environmental contamination from inadequate attention to management of faecal waste and poorly maintained septic systems, both of which are likely to contribute to suboptimal health impacts in high-density living settings such as occurs in urban informal settlements. The RISE study will also concurrently address upgrading of physical access within communities to limit residents' exposure to environmental pollutants, improve overall water cycle management, diversify water source supplies, and attend to water drainage and flood management. This holistic, settlement-scale approach aligns with recent calls for "transformative WASH" or "WASH-plus" solutions, incorporating a more comprehensive whole-of-systems framing to address a major planetary health challenge[12 13].

Here we describe the rationale of the RISE intervention and the study design and methods of a cluster randomized controlled trial (RCT) to assess the impact of the intervention on the environment and human health and wellbeing. The cluster RCT is being conducted in Makassar, Indonesia and Suva, Fiji in cooperation with local and national governments.

## HYPOTHESES AND AIMS



For peer review only - http://bmjopen.bmj.com/site/about/guidelines.xhtml

The hypothesised sequence of change in the RISE study is that implementing a water sensitive intervention in informal settlements leads to improved physical environments, which in turn results in reduced human exposure to pathogenic faecal contamination and flooding hazards, resulting in improved human and ecological health and wellbeing (Figure 1). Specifically, we hypothesise that the intervention will reduce environmental contamination with human faeces. We further hypothesise that this will reduce residents' exposure to gastrointestinal pathogens that cause diarrhoeal disease, poor gastrointestinal function, and nutritional deficits, which particularly impact on young children living in informal settlement conditions, as well as reducing exposure to faecal sources of antimicrobial resistance (AMR) genes. Our hypotheses align with the planetary health approach[14], acknowledging the complex interplay between multiple factors which include health, environmental conditions, urbanisation, water and sanitation management, gender and socio-economic equity, and climate change.

We have three central scientific questions and aims that align with our hypotheses.[1]

***Aim 1: Environment***

We will first assess whether the RISE intervention leads to improved environmental characteristics. Urbanisation alters physical environmental conditions such as air temperature, soil contamination, and water quality, and causes transformations in local biodiversity and changes in ecological communities and their functioning[15-18]. Cities tend to have warmer environments than surrounding areas due to the urban heat island effect[19 20], and in tropical informal settlements, high housing density and inadequate formal solid, greywater and faecal waste processing pathways result in considerable eutrophication and faecal contamination of soil and water[21]. Increased populations of mammal (e.g. rats) and arthropod (e.g. mosquito) vectors are often present[22-24], likely due to a combination of thermal changes, habitat modification, resource concentration, and high human population densities[25-27].

Our aim is to assess the impact of delivery of the water sensitive intervention on the physical, thermal, hydrological and microbiological environments. Changes are expected to make residents less vulnerable to the impacts of flooding, reduce the abundance of vectors, increase the diversity of urban ecological communities and alter the composition of microbial communities. Additionally, by improving human excreta management strategies, we aim to decrease faecal contamination of the environment, thereby reducing exposure to pathogens and AMR genes in water and soil.

***Aim 2: Human health***

Our second aim is to assess whether the intervention leads to improved physical health outcomes as a result of improved environmental conditions, as hypothesized above. Prevailing bio-physical conditions in informal settlements are conducive to the spread of infectious diseases. Ample vector breeding sites and poor preventative practices lead to mosquito-borne diseases[28 29]. Children suffer from diarrhoea and poor nutrition [30 31], with contaminated water and soil acting as a source for transmission of pathogens derived from animal, human, and environmental origins[32 33]. Antimicrobial-resistant infections are also a growing threat to human health globally[34], and inadequate faecal waste management leads to environmental contamination which may promote transmission of multi-drug resistant pathogens between humans and the environment[35 36]. Young children under the age of five living in these suboptimal conditions are at especially high risk for under-nutrition, diarrhoea, and poor growth and development. Further worsening their physical health is the high prevalence of environmental enteric dysfunction, a chronic inflammatory condition of the intestine which is associated with poor absorption of nutrients, growth faltering, and impaired response to live enteric vaccines[37 38].

---

[1] In addition to these three scientific questions, the RISE program includes two other research objectives: (1) to develop novel practices for built environment and community co-design, and (2) to develop the financial and policy frameworks for scaling up the intervention.



For peer review only - http://bmjopen.bmj.com/site/about/guidelines.xhtml

By designing and delivering decentralized infrastructure appropriate to and scalable within informal settlements, we aim to improve faecal sludge handling and reduce contact with faecal contamination in order to interrupt the connection between the human faecal stream and the environment. These impacts are expected to decrease children's exposure to gastrointestinal pathogens, as detected by molecular methods in faecal samples, to decrease environmental enteric dysfunction, and to decrease the abundance and diversity of AMR markers detected in children's gastrointestinal tract. Flood mitigation and improved access within communities will further decrease human contact with the overall reservoir of environmental pathogens, and falling vector abundance may decrease mosquito-borne disease.

***Aim 3: Wellbeing***

Our third aim is to determine whether the intervention affects the physical environment in ways which impact on community wellbeing and individual subjective psychological and economic wellbeing. Changes to the physical environment affect inhabitants' lifestyle, mental health, self-image, perceptions of safety, and social cohesion[39-41]. They also impact on water security, local economic activities, financial wellbeing, time allocation for water collection and time available for paid work[42-46].

We aim to change the physical environment to positively impact inhabitants' evaluative and hedonic subjective wellbeing; change how people allocate their time; and improve economic activity by increasing employment opportunities and time spent on income-generating activities. At a community level, we aim to improve residents' perceptions of social cohesion and, motivated through the local co-design approach during intervention development, improve community resilience by enhancing collective efficacy.

***Additional outcomes***

In addition to these three key scientific aims, we will evaluate the evidence on the feasibility and impact of the approach to explore the potential for scaling up water sensitive interventions throughout the Asia-Pacific region through rigorous generation of evidence. We will engage with governments, investors and implementers to determine priorities and economic drivers, and identify barriers to intervention to address the environmental challenges of urban informal settlements.

## STUDY METHODS AND ANALYSIS

### Overview of the study design and timeline

To evaluate the RISE intervention, we are conducting a cluster RCT involving 12 informal settlements in Suva, Fiji, and 12 in Makassar, Indonesia, with clustering at the settlement level. In each country, half of the sites have been randomised as intervention communities and will receive an early upgrade. The remaining communities will serve as controls and will receive only standard, basic hygiene and sanitation messages. Monitoring and assessment will be conducted in all settlements over an approximate five-year timeline which will include baseline data collection, the design and build implementation phase of the project, and 2 years of post-build monitoring (Figure 2). After completion of the study, the intervention will be implemented in the 12 control settlements.

### Study settings

The study is being conducted across two countries where large portions of the population are subject to inadequate water and sanitation infrastructure. Indonesia and Fiji were purposively selected to represent a diversity of characteristics across the Asia-Pacific which might influence intervention effects, including climatic factors, water security, tidal inundations, population density, sanitation practices, and socioeconomic and cultural conditions. Within each country, candidate sites were selected in consultation with local government authorities, intervention funders, research partners, communities, and other organisational stakeholders. Formative work consisted of four phases: a) extensive reconnaissance visits to both cities to understand the range of site conditions and to scope potentially suitable sites, b) early



For peer review only - http://bmjopen.bmj.com/site/about/guidelines.xhtml

engagement with local and national governments to determine priorities and preferences, and to ascertain tenure status for each site, c) household enumeration surveys to determine approximate population size and demographics in each settlement, and d) extensive discussions with community leaders and residents to determine their willingness to participate and priorities.

Makassar, the provincial capital of South Sulawesi, has seen rapid urbanisation and population growth in the last decade, with unplanned city growth resulting in a dramatic rise of informal settlements. The 12 settlements of the RISE project are dispersed across the city and represent a diversity of settlement typologies. While the settlements are located in various flood plains and coastal areas, they are all characterised by dense and precariously built housing, little open space, poor vehicular and pedestrian access, and water stressors. Water bodies and soil are typically contaminated with solid waste derived from animal and human sources, and with domestic black and grey wastewater. Seasonal and other flooding is common, requiring makeshift planking for access. Despite some variation in socio-economic status within and across settlements, sites typically house the most economically vulnerable populations. Households across the settlements predominantly live on small parcels of privately-owned land often with one house, or in some instances two or three houses occupied by extended family groups, with varying degrees of land tenure security.

Similar to the settlement sites in Indonesia, the 12 RISE informal settlements in Suva, Fiji, reflect a range of typologies that exist in Fiji and the broader Pacific context. While the urban morphology varies between settlements, they are typically high-to-medium density clusters with some open space and, in some instances, lush vegetation. Some settlements are prone to significant pluvial and fluvial flooding; housing is of varied quality and construction type but is typically characterised by lightweight structures raised off the ground. Tenure status is characteristic of informal settlements in Fiji and the broader Pacific region with limited private land ownership in the selected communities, instead relying on community leases on state land or informal agreements with traditional land-owning units on native lands.

### Settlement eligibility and selection criteria

Data gathered on potential sites characterised local water supplies, sanitation systems, and flooding events and risk. Inspections were conducted at potential sites to confirm feasibility and accessibility. Site locations and sizes were informed by the UN-Habitat informal settlement selection approach[47]. Key inclusion criteria for settlements consisted of location in urban areas of Makassar and Suva, suitability for delivering the planned intervention, and community receptiveness and commitment to study participation. Final selection criteria consisted of settlement size, environmental, and construction related factors (**Table 1**). The key individual inclusion criterion is residence in a settlement enrolled in the trial.

| **Table 1: Selection criteria for informal settlements** | |
|---|---|
| Settlement size, location, and demographics | 1. 30-100 houses, ideally all in one consolidated group<br>2. Physically separated from other settlements with clear physical boundaries<br>3. No conditions calling for future permanent relocation (i.e. landslide risk)<br>4. Representing the most vulnerable populations of the city<br>5. Location in vulnerable areas with presumed high risk of water-borne disease<br>6. Presence of at least 5-10 children under 5 years |
| Land tenure | 7. Occupants with secure tenure (ownership of the house and land that it sits on, or permission to occupy) |



For peer review only - http://bmjopen.bmj.com/site/about/guidelines.xhtml

| | |
|---|---|
| Environmental conditions | 8. Presence of water stressors including flooding, poor drainage, limited sanitation and water supply |
| Construction related | 9. Potential for replication and scaling of designs |
| Participation | 10. Settlement leaders and inhabitants consenting for health and environmental assessments and infrastructure modification |

**Site and household recruitment**

Community recruitment occurred after extensive discussion with stakeholders, government, community leaders and householders, and was performed by trained local community field worker teams. The unit of recruitment for assessment of the intervention is at the household level. We have attempted to recruit and enroll all households within the agreed boundaries of each informal settlement, with written informed consent obtained from heads of households. Householders were able to consent to participation in the overall RISE project with or without consenting to additional individual survey and sampling components.

**Description of the intervention**

The water sensitive approach to upgrading implemented in the RISE study integrates urban design and urban water cycle planning and management, focusing on water conservation and reducing environmental pollution by improving the quality of wastewater and stormwater prior to reuse or discharge to the environment. Implementation of water sensitive infrastructure will be site-specific, co-designed with each community, and adapted to the different biophysical and sociocultural conditions in each informal settlement. The intervention includes specific components collectively designed to reduce residents' exposure to faecal contamination, requiring evaluation of critical faecal contamination and community exposure pathways at each site to determine optimal available technology solutions. Delivery of traditional approaches to water and sanitation services such as private toilets and hand basins are incorporated. Importantly, solutions also include installation of broader decentralised water infrastructure options to control and reduce the release of contaminated sewage and household greywater into the environment, improve flood mitigation, lower exposure to polluted environments within settlements through better access options, and provide source water availability through rainwater harvesting and wastewater recycling.

Specific infrastructure options include (Figure 3):

- New integrated 'wetpods': these are comprised of a toilet, hand basin and rainwater tank;
- Toilet connections: pressure sewers and traditional collection and treatment systems (pipes, manholes, septic tanks and drains);
- Wastewater treatment: constructed wastewater treatment wetlands for black water[48-50], and biofilters and biofilter drains for grey water[51-54];
- Stormwater drainage and treatment: this includes resizing, reprofiling and formalising drains, and swales, raingardens and constructed stormwater treatment wetlands and permeable paving[55-58];
- Water supply security: rainwater tanks and collection, connection to municipal water supply, water supply disinfection and protection of existing shallow well water supplies;
- Access roads, utility corridors, raised pathways;
- Flood management: including backflow prevention, minor localised spot-filling, minor terrain modification and flood protection walls.

The water sensitive approach has been shown to successfully address urban water management challenges in an integrated way in high income economies[59-66], and will be adapted for informal settlements in the RISE study. We will initially design and deliver a pilot project in each country to test key water sensitive technologies and evaluate soils and plants best suited to local conditions. Monitoring



For peer review only - http://bmjopen.bmj.com/site/about/guidelines.xhtml

system performance from the two pilot communities will underpin adaptive design adjustments to full-scale implementation.

As a socio-technical intervention, infrastructure design and delivery will be adapted to context-specific social and bio-physical characteristics and constraints of each community through a participatory process of co-design. The co-design process will promote engagement and informed decision-making with residents of each settlement, particularly emphasising participation by women, children, elderly, disabled and other groups that may have specific needs. The process is characterised by active community participation to ensure participants' preferences for solutions are heard, including informed choices regarding the physical location of new infrastructure. It brings the international community of researchers and practitioners together with the residents living in the study communities, thereby building understanding and a sense of value of the infrastructure to increase the likelihood of success of long-term operations and maintenance. Communal infrastructure will be delivered by local contractors supervised by the project team. Long-term operation and maintenance responsibilities will be addressed via training and ongoing engagement of community representatives and local authorities. By utilising local construction expertise and engaging and empowering stakeholders at household, community, and city levels, we aim to build knowledge and trust with local communities, institutions, and governments to ensure the infrastructure is built and maintained for sustainable, long term benefit.

### Data collection

Data collection is conducted by field workers specifically employed for assessment of trial outcomes; this team is separate from the personnel implementing the intervention. Data collection comprises biospecimen sampling at both the settlement and household levels, field data measurements, and household surveys. Specimen collection involves blood and faeces from children under the age of 5 years; environmental soil, water and animal scat samples; and vector trapping, with intended sampling frequency as shown in Table 2. Ecological field data assessment involves continuous passive acoustic monitoring, temperature logging, and flood assessment including the use of satellite imagery. Anthropometric measures are taken annually among children under aged 5 years. Surveys are administered quarterly, with different modules asked of varying-aged participants to assess physical health, healthcare utilisation, psychological wellbeing, financial wellbeing, time-use, and perceptions of social cohesion and collective efficacy. Questions regarding household level data (i.e. household member composition, flooding) are administered preferentially to the adult female head of household and questions regarding children under the age of 15 are preferentially directed to the adult caregiver who has seen them most days in the past 7 days. Questions regarding adult health and wellbeing, financial wellbeing, time-use, and perceptions of social cohesion and collective efficacy are preferentially administered to: (i) primary caregivers where households contain children under 15 years, and (ii) female or (iii) male heads for households with no children. If the preferred respondent is not at home, field teams attempt two re-visits. To maintain participation over the trial period and to show appreciation for the time spent by participants on study-related activities, we will provide small culturally appropriate gifts of nominal value, as recommended by our in-country partners.

**Table 2: Data collection tools and specimens for primary and secondary outcomes**

| | Minimum Frequency* | | | |
|---|---|---|---|---|
| **Primary outcomes** | C | Q | B | A |
| Faeces collection, children < 5 years of age for: | | | X | |



For peer review only - http://bmjopen.bmj.com/site/about/guidelines.xhtml

| | | | | |
|---|---|---|---|---|
| • Prevalence, diversity and concentration of bacterial, viral, protozoan, and helminth gastrointestinal pathogens in children < 5 years of age (composite primary outcome)<br>• Concentration of intestinal inflammation markers representing environmental enteropathy in children < 5 years of age<br>• Diversity and abundance of AMR genes in gastrointestinal microorganisms of children < 5 years of age (composite primary outcome) | | | | |
| **Secondary outcomes** | | | | |
| ***Physical Health Measures*** | | | | |
| Blood collection for haemoglobin and blood inflammatory markers, children < 5 years | | | | X |
| Anthropometry measures (length-for-age, weight-for-age and weight-for-length Z-scores) measure, children < 5 years of age | | | | X |
| ***Surveys: Household and community demographic, health and wellbeing*** | | | | |
| Household members and demographics | | | | X |
| Household member behaviors and exposures | | | X | |
| Access to sanitation, water, and hygiene facilities^ | | | | ^ |
| Caregiver-reported acute symptoms (diarrhoea, respiratory infections and febrile illness), children < 5 years of age | | X | | |
| Healthcare system utilisation, children < 5 years of age | | X | | |
| Self-reported or caregiver-reported acute symptoms, healthcare system utilisation, and subjective general health (5 point rating scale) among adults and children 5-15 years | | | X | |
| Caregiver assessed quality of life (Pediatric Quality of Life Inventory, PedsQL, emotional functioning dimension), children 5-15 years | | | X | |
| Self-reported life satisfaction – multiple domains (10 point rating scale), adults | | | X | |
| Center for Epidemiologic Studies Depression Scale [CES-D-10], adults | | | | X |
| Self-reported major adverse life events, adults | | | X | |
| Time-use surveys, children 5 to 15 years and adults^ | | | | ^ |
| Housing tenure+ and collective efficacy (Likert 5 point scales) | | | | X |
| Indicators of household assets and expenditure^ | | | | ^ |
| Intermittent qualitative surveys and focus groups | NA | | | |
| ***Measures of environmental faecal contamination*** | | | | |
| Soil and water (potable, recreational, wastewater) samples and animal scat samples for chemical and microbiological analyses (including markers of faecal contamination; prevalence, diversity and concentration of pathogens; abundance and diversity of AMR genes) | | X | | |
| ***Ecological measures and biodiversity*** | | | | |
| Indoor and outdoor thermal data logging | X | | | |
| Passive acoustic monitoring | X | | | |
| Aerial and satellite imagery | | | | ^ |
| Flood and rain gauges+ | | | | X |
| Mosquito trapping | | X | | |
| Rodent trapping+ | | | | ^ |
| *C = continuous; Q = quarterly; B = Biannual; A = Annual ^First and final years of study +Indonesia baseline survey only | | | | |

**Public involvement**



For peer review only - http://bmjopen.bmj.com/site/about/guidelines.xhtml

Members of enrolled communities are involved in the design and conduct of this trial, including the design and implementation of the intervention as described above.

## OUTCOMES

### *Primary outcomes*

We define our first primary outcome as the prevalence and diversity of bacterial, viral, protozoan, and helminthic gastrointestinal pathogens in children under 5 years. Additional primary outcomes are i) the abundance and diversity of AMR genes in gastrointestinal microorganisms of these children; and ii) the concentration of intestinal inflammation and permeability biomarkers in stool representing environmental enteric dysfunction.

We will use various molecular approaches to assess the presence and concentration of pathogens, microbial communities, and AMR genes in human faecal samples. As indicated below, the same methodologies will be applied for processing of environmental samples, thereby optimising assessment of the impact of the intervention on both environmental and human health outcomes. Total nucleic acids will be extracted from faecal samples in-country. Pathogen levels in samples will be measured by quantitative PCR (qPCR) using TaqMan Array Cards [67 68] targeting >30 bacterial, viral, protozoan and helminthic pathogens. rRNA gene amplicon sequencing will measure microbial biodiversity. Shotgun metagenomic sequencing will be used to gain information about the functional capabilities of pathogens in each sample, including the classification and enumeration of AMR genes. Additionally, over 80 AMR genes will be directly detected by qPCR. Expected environmental enteric dysfunction serological markers include faecal myeloperoxidase[69], faecal α-1-antitrypsin[70], and faecal neopterin[71].

### *Secondary outcomes*

<u>*Human health secondary outcomes*</u>

Secondary health outcomes include caregiver- and self-reported acute symptoms, healthcare utilisation, inflammatory markers in blood, haemoglobin levels, and anthropometry measures. Caregiver- and self-reported symptoms will be collected via quarterly standardised questionnaires throughout the trial, including the presence of diarrhoea, respiratory and undifferentiated febrile illness over the 7 days prior to the survey visit[72]. Symptoms will be assessed quarterly in children under the age of 5 and every 6 months in children between 5-15 years of age and adults. In all cases, diarrhoea is defined as ≥ 3 loose or watery stools in a 24-hour period. Healthcare utilisation (visitation to healthcare provider, hospitalisation, antibiotic use) by children under 5 years of age within the three months prior to the visit will be surveyed quarterly; for children 5 -15 years of age and adults surveys will be 6-monthly.

Blood from children under 5 years of age will be taken annually by venipuncture. Haemoglobin levels will be measured in the field using a point-of-care device. Plasma and whole blood will be stored for further analyses which will include markers of nutrition and inflammation. To obtain length-for-age, weight-for-age and weight-for-length z-scores in children under 5 years of age, trained staff will follow standard procedures for anthropometric measurements[73]. Pairs of trained anthropometrists will measure standing (in children between 2 and 5 years of age) or recumbent (less than 2 years of age) length (accurate to 0.1 cm) and weight without clothing (accurate to 0.1 kg) in triplicate. In both cases, the median of the three measurements will be used in the analysis[74].

<u>*Community and individual wellbeing secondary outcomes*</u>

Secondary outcomes related to individual and community wellbeing include self-reported general and emotional wellbeing, time-use, measures of collective efficacy and water security, and indicators of socioeconomic status. We will assess the subjective general health and emotional wellbeing of children aged 5-15 years using the (parent-proxy) Paediatric Quality of Life InventoryTM 4.0 Emotional



For peer review only - http://bmjopen.bmj.com/site/about/guidelines.xhtml

Functioning Scale (PedsQL)[75]. Subjective general health, life satisfaction and major life events (i.e., family death, serious illness, marriage break-up, bankruptcy, victim of crime) of adults will also be collected 6-monthly[76 77]. Child (age 5-15 years) and adult time-use (e.g. minutes/hours spent on different activities in past week) will also be collected. Gender aspects will be examined, including analyses of sex-disaggregated schooling attendance and the intervention's impacts on water collection time and wellbeing for females in our sample. We will assess collective efficacy to monitor changes in community wellbeing, trust, and feelings of safety[78]. Indicators of socioeconomic status (i.e., education levels, house building materials, assets owned, bank account, primary activity, time spent working and subjective financial wellbeing) will also be collected to facilitate distributional analyses of any observed impacts. We will also estimate the costs of the intervention and report on cost-effectiveness and costs-benefits.

*Ecological and environmental secondary outcomes*

Secondary outcomes for environmental effects include quarterly chemical and microbiological measures to assess faecal contamination of both soil and water (municipal supply, recreational and wastewater), quarterly measures of vector abundance, and continuous measures of the thermal environment and overall biodiversity. We will compare faecal contamination in water and soil samples across intervention and control settlements. Sample collection and transport will be performed using previously described and optimised methods[79 80] and processed for faecal bacteria quantification[81]. Nucleic acid extraction will be performed in-country via methods that enable simultaneous detection of viruses, bacteria, protozoa, and helminths from environmental samples, as confirmed through optimisation experiments already performed. As with human faeces, pathogen presence and concentrations in samples will be measured using TaqMan Array Cards ([67 68] and AMR markers will be detected by qPCR and shotgun metagenomic sequencing. rRNA gene amplicon sequencing will be performed to determine microbial community composition and diversity[82 83]. The amplicon outputs will also be used for microbial source tracking using the SourceTracker tool[84 85]. Changes to the thermal environment will be assessed at the settlement level through remote sensed data supported by local thermal data and compared with changes to the built environment over time[86]. Continuous temperature tracking will use thermal dataloggers (Thermochron iButtons™). Ten randomly selected houses in each settlement will have thermal data loggers installed and a further 5 thermal data loggers will be deployed across the settlement to measure ambient temperature and humidity. Rainfall data will be derived from multiple (where available) local government-maintained weather stations enhanced with tipping bucket rain gauges installed to maximise coverage across the study area.

We will examine mosquito species variation and abundance over time based on quarterly trapping results. Mosquitoes will be sampled using BG Sentinel II traps[87] with 15 traps deployed per settlement, inside or outside randomly selected houses. Trap location will remain consistent throughout the assessment period. Mosquitoes will be identified to species under dissection microscopes using morphological keys[88-90]. Relative abundance and species composition of rodents in communal areas of settlements will also be surveyed in Makassar at baseline using standard traps and a grid-based trapping design to equate trapping effort across sites[91].The influence of season and physical environmental features, including temperature, humidity and rainfall, on the relative abundance of vector species will be examined[92 93]. Continuous passive acoustic monitoring at settlements will provide a measure of overall biodiversity[94]. The acoustic environment will be monitored using one or two (depending on settlement size) SongMeter SM4 bioacoustic recorders per site for human-audible sound frequencies. Additionally, in Indonesia we will deploy a single SongMeter SM4BAT recorder for ultrasonic frequencies emitted by echolocating bats at each site.

***Referral guidelines***



For peer review only - http://bmjopen.bmj.com/site/about/guidelines.xhtml

The study will refer participants for treatment to appropriate local healthcare providers in cases of acute malnutrition, anaemia, and intestinal helminth infection. WHO weight for length z-scores will be calculated in the field during anthropometry measurements for children under 5 years of age; children meeting WHO/UNICEF criteria for severe malnutrition (weight for length z-score ≤ 3) will be referred to a local health facility. At times of blood draws, all participating children under age 5 years will have their haemoglobin measured in the field. Children with severe anaemia (haemoglobin < 7.0g/dL) will be referred to a local health facility. A subset of faeces samples will be immediately assessed for helminths using the Kato-Katz technique, and individuals with positive results will be referred for treatment.

**Randomisation**

Randomisation was performed separately for each city: March 2019 in Makassar and November 2019 in Suva (Figure 2). Randomisation occurred following settlement recruitment and completion of 12 months of baseline surveys. Settlements (clusters) have been randomly assigned to either intervention or control groups, with equal numbers in each group. Covariate-constrained randomisation was utilised using the cycrand package in Stata V15 to achieve balance between the intervention and control groups on key baseline factors determined *a priori*.

For Makassar, intervention and control groups were first balanced on number of children aged under 5 years (4 settlements in each group: ≤10, 11-30 and >30 children) and flood risk (high and low, 6 settlements in each), producing 90 allocations. Subsequently, the 30 allocations most imbalanced on average asset score were removed, with the score determined from each household's ownership of selected assets. For Suva, intervention and control groups were first balanced on number of children aged under 5 years (<40 children, 8 settlements; >40 children, 4 settlements) and flood risk and site contamination grouping (2 extreme risk, 2 high risk, and 8 medium risk settlements). This resulted in 120 allocations, from which the 30 most imbalanced allocations of asset score were removed, followed by the 30 most imbalanced allocations of average number of children under 5 years. For each of the cities, this resulted in the 60 best balanced allocations being used for the randomisation (total 3600 [60 × 60] possible allocations for the trial). A randomisation ceremony was held in both cities, where a child from the pilot community selected a ping pong ball from a large glass jar that contained 60 ping pong balls numbered 1 to 60, representing the 60 determined random allocations that achieve the balance criteria specified above.

**Sample size**

The choice of 12 settlements per city, each of ~55 dwellings with 5–6 people per dwelling, has been made to balance the need for statistical power with the constraints of construction logistics and cost. Statistical power was calculated using formulae for cluster RCTs with repeated assessments of a cohort[95] (and confirmed by numerical simulation using generalised linear mixed models) for the three primary health outcomes assessed in children aged under 5 years: i) prevalence of gastrointestinal pathogens and diversity (number of bacterial, viral and parasitic gastrointestinal pathogens per sample); ii) diversity and abundance of AMR markers; and iii) concentration of intestinal inflammation markers. For measures of diversity and abundance of markers of drug resistance in environmental samples, the power calculations assumed the same intra-cluster correlations as for the health outcomes (see below).

For the health outcomes involving subsampling of an average 30 children under 5 years of age per settlement, there is 80% power to detect, from 6 months post-construction and beyond, a 40% relative reduction in the prevalence of gastrointestinal pathogens (assuming a baseline prevalence of 25%) and a 31% relative reduction in the average count of enteric pathogens per child (assuming a baseline average count of 1.0 and overdispersion factor of 2; with a baseline of 2.0 the relative reduction is 23%), and at 12 months an absolute difference of 0.30 SDs (and 0.34 SDs for environmental assessments) in the average concentration of intestinal inflammation markers and average number and abundance of AMR markers.



For peer review only - http://bmjopen.bmj.com/site/about/guidelines.xhtml

These calculations assume intra-cluster (i.e. within-settlement) correlations between: two children within the same time period 0.10[7]; two children in different time periods 0.067; and repeated measurements of the same child 0.10. The relative reduction of 30% has been informed by the findings reported in systematic reviews, which have found reductions of 30–50% due to water-quality interventions[96 97].

### Statistical analyses

Analyses will be by intention to treat. Descriptive statistics will report characteristics of settlements and of individuals by randomized arm. Estimation of the effects of the intervention for each outcome will use generalised estimating equations with an exchangeable correlation structure and robust standard errors clustered at settlement level, scaled with a small-sample degrees-of-freedom adjustment[98]. Data from 6-, 12-, 18- and 24-months follow-up visits will be included in the analysis and the intervention effect estimated for the 6 month follow-up timepoint for prevalence and mean number of gastrointestinal pathogens, and at 12 months follow-up for diversity and abundance of AMR markers and for intestinal inflammation markers. For binary and count outcomes, we will use a logarithmic link to estimate ratios of prevalence and mean counts, respectively; for continuous outcomes, the identity link will be used to estimate differences in means. All models will adjust for the baseline covariates, number of children under 5 years, flood risk category (see Randomisation section) and asset score. To enable temporal adjustment, indicator variables for seasonal patterns (e.g. rainy versus dry), local rainfall data for each site, and an indicator term for any major flood event at a particular settlement in a particular quarter will be included in the models. For households that drop out of the study, we will employ multiple imputation using imputation models that preserve the hierarchical data structure. Additional sensitivity analyses will exclude households who moved into the settlement post-construction. There will be no adjustment to the p-values for the assessment of multiple primary health outcomes, however, all findings will be fully reported and interpreted based on incremental evidence.

We will explore relationships between environmental changes and health outcomes by modelling health outcomes at 12-, 18- and 24-months post-construction, using changes in environmental measures at 6-, 12-, or 18-months post-construction, respectively, as principal covariates. We will estimate these effects using generalised estimating equations with pre-construction health and environmental parameters as baseline covariates aggregated at settlement-level, and will include interactions of environmental changes with the intervention for effect-modification assessment, together with adjustment for potential confounders.

Laboratory technicians conducting sample analyses will be blinded to the intervention assignment throughout the study duration through the use of barcodes for sample identification. Owing to the nature of the intervention, community participants will not be blinded.

### Study oversight

The RISE study has an independent International Scientific Advisory Panel (ISAP) and a Governance Committee, both of which provide oversight and advice including in relation to any unintended effects of the trial intervention or trial conduct. The ISAP committee will advise on ethical issues that arise. The study also has a Data Advisory Panel, comprised of representatives across Monash University who provide strategic advice and operational guidance to assure a holistically-advised research data strategy. Given the nature of this trial, there are no stopping rules. Results of the intervention implementation in the first 12 settlements will be used to inform and optimise rollout in the control settlements following completion of the trial.

### Limitations, justifications and additional analyses



For peer review only - http://bmjopen.bmj.com/site/about/guidelines.xhtml

Water and sanitation trials aiming for community-level intervention coverage pose great challenges for trial designers[99]. The relative lack of RCTs of such interventions reflects multiple inherent difficulties, including randomly allocating networked infrastructure, the long follow-up and large sample sizes required to study health outcomes, and the need for a complex engineering and construction phase[13]. The RISE study will deliver site-specific and bespoke interventions to each settlement, and while this is deliberate and a strength of our approach as it will address site-specific needs, it also results in a lack of intervention uniformity across communities. The purposive selection of settlements included and the relatively small size of each community compared with many informal settlement settings, while necessary from a logistics perspective, also limits broad external validity. Furthermore, while we intend to monitor the sustainability of the intervention, the RCT is funded to perform formal monitoring for an initial 2 years post intervention delivery.

Complete settlement revitalisation should ideally encompass a broad suite of measures incorporating comprehensive upgrading of solid waste management; improvements in housing conditions, roads, street lighting and other amenities in communal areas; and delivering a range of other services. However, to test our specific hypothesis (and given logistic and financial constraints), our planned site-specific intervention is restricted in scope to addressing many of these issues predominantly as they relate to our primary focus of delivering improved water and sanitation infrastructure and reduced flooding impacts, in order to minimise human exposure to environmental faecal contamination. For example, attention to solid waste management is important to ensure that the wetlands, which treat the wastewater on-site, will function as expected and not become blocked with solid waste, as this would reduce the ability of wetland plants to remove contaminants from the wastewater and potentially become a breeding site for vectors (i.e. mosquitoes).

Studies such as RISE which involve complex infrastructure upgrades face methodological evaluation challenges and cannot be performed in a blinded fashion, thereby potentially introducing biases in survey-based outcomes. For trials aimed at improving gastrointestinal health among children, the reliability of caregiver-reported diarrhoea is highly variable and in unblinded trials is particularly subject to bias[100]. An alternative approach of testing for organisms in human faeces therefore has advantages, but does not reliably distinguish between non-pathogenic carriage versus causal links of enteropathogens to diarrhoea[101] or impaired nutrient absorption and growth[102 103]. Hence, we will monitor a combination of primary objective outcomes as well as secondary clinical outcome biomarkers to assess children's physical health. However, while we have described our planned methodologies for analytical testing, the multi-year timeline of the RISE trial means that analytical methods may improve during the study (for example, methods for measuring environmental enteric dysfunction), and accordingly we may need to make changes to the specific tests mentioned in this protocol. The results of our initial analyses to identify which pathogens and AMR genes are most prevalent in the study areas will inform decisions regarding optimal approaches for detection throughout the study period.

Defining primary versus secondary aims and outcomes from complex multifactorial community upgrades requires somewhat artificial prioritisation of transdisciplinary effects. This reflects that anticipated impacts of multi-dimensional interventions on communities occur along an interconnected causal chain rather than in a simple hierarchical and linear cause-and-effect fashion. While we have pre-specified our primary outcomes to reflect objective markers of gastrointestinal health in children under the age of 5 years, our fundamentally transdisciplinary intervention and assessment protocol appropriately accounts for the complex interconnections between ecosystem health, environmental contamination, vector abundance, physical health and wellbeing. Our intervention has potential to improve liveability through improved access and opportunities for use of community spaces, better economic sustainability through improved micro-economies and urban farming opportunities, and climate resilience through the diversification of water supplies and flood management. We will therefore assess a range of additional secondary, albeit



For peer review only - http://bmjopen.bmj.com/site/about/guidelines.xhtml

equally important, impacts on environmental and ecological health, as well as on psychological, social and economic wellbeing outcomes, which we will measure at both the individual and community level. Our multi-faceted monitoring approach and our deliberate focus on assessing the links between human and environmental health will enable us to populate a novel conceptual planetary health model with a broad suite of real-world data. This will advance understanding of mechanisms and impacts of interactions between individual health indicators, and provide a surveillance and analytical framework for future studies.

There are significant challenges with both implementing and measuring impacts of infrastructure upgrades, and for determining which components of an intervention contribute most effectively to observed outcomes[104-106]. Co-design interventions and upgrading programs have often failed to include a counterfactual or have taken a quasi-experimental approach[107], and previous studies have often used narrowly focused or proxy markers of effect rather than direct outcome measures[104-107]. Our planned pragmatic cluster-RCT approach to rigorously assess intervention effects is a notable strength. However, the RISE program involves a complex intervention implemented into complex dynamic communities. Thus, we anticipate a range of outcomes, including potentially important consequences that we cannot accurately prespecify. While we can be most confident of the design-based inference from prespecified outcomes, we also foresee contributing broadly to literature on the impact of the interventions across a wider array of outcomes beyond those explicitly mentioned in this protocol, with findings interpreted based on direction, magnitude and incremental evidence. Additionally, data and specimen collection tools not mentioned here will be appended to address supplementary questions, thereby enabling examination of additional health, environmental and social assessments in the participating informal settlements.

## ETHICS AND DISSEMINATION

Ethics review and approval was provided by participating universities and local IRBs, including Monash University Human Research Ethics Committee (Melbourne, Australia; protocol 9396), the Ministry of Research, Technology and Higher Education Ethics Committee of Medical Research at the Faculty of Medicine, Universitas Hasanuddin (Makassar, Indonesia; protocol UH18020110), and the College Human Health Research Ethics Committee (CHREC) at the Fiji Institute of Pacific Health Research (FIPHR) and College of Medicine, Nursing, and Health Sciences at Fiji National University (FNU) (Suva, Fiji; protocol 137.19). Animal ethics for rodent trapping has also been secured (Monash University project ID16351; Universitas Hasanuddin UH18080446). The trial is registered on the Australian New Zealand Clinical Trials Registry (ANZCTR) (Trial ID: ACTRN12618000633280). Prior to providing informed consent, all study settlements, households, and caregivers/respondents are given written explanatory statements describing the voluntary nature of study participation and the fact that they can withdraw from any or all of the study components at any time. Verbal consent is affirmed and documented at each subsequent approach prior to proceeding with survey and sample collection. The data collected in the study will be publicly distributed along with metadata and critical documents (i.e, protocols and questionnaires) following the publication of the primary results from the trials.

### Figure Legends

Figure 1: The RISE study hypothesised theory of impacts. The water sensitive intervention will improve the physical environmental conditions, resulting in both direct and indirect impacts on ecology and human physical health and wellbeing

Figure 2: Anticipated RISE study timelines

Figure 3: Schematics of proposed sanitation intervention



For peer review only - http://bmjopen.bmj.com/site/about/guidelines.xhtml

## References


1. United Nations. Sustainable Development Goals Report, 2019, New York. Accessed from https://unstats.un.org/sdgs/report/2019/The-Sustainable-Development-Goals-Report-2019.pdf, 13th June 2020.
2. Turley R, Saith R, Bhan N, et al. Slum upgrading strategies involving physical environment and infrastructure interventions and their effects on health and socio-economic outcomes. *Cochrane Database of Systematic Reviews* 2013(1.)
3. Ezeh A, Oyebode O, Satterthwaite D, et al. The history, geography, and sociology of slums and the health problems of people who live in slums. *The Lancet* 2017;389(10068):547-58.
4. United Nations. SDG Indicators, 2020. Accessed from https://unstats.un.org/sdgs/report/2019/Goal-11/, 13th June 2020.
5. Lilford RJ, Oyebode O, Satterthwaite D, et al. Improving the health and welfare of people who live in slums. *The Lancet* 2017;389(10068):559-70.
6. Weimann A, Oni T. A Systematised Review of the Health Impact of Urban Informal Settlements and Implications for Upgrading Interventions in South Africa, a Rapidly Urbanising Middle-Income Country. *International Journal of Environmental Research and Public Health* 2019;16(19):3608.
7. Clasen T, Boisson S, Routray P, et al. Effectiveness of a rural sanitation programme on diarrhoea, soil-transmitted helminth infection, and child malnutrition in Odisha, India: a cluster-randomised trial. *Lancet Glob Health* 2014;2(11):e645-53.
8. Luby SP, Rahman M, Arnold BF, et al. Effects of water quality, sanitation, handwashing, and nutritional interventions on diarrhoea and child growth in rural Bangladesh: a cluster randomised controlled trial. *Lancet Glob Health* 2018;6(3):e302-e15.
9. Null C, Stewart CP, Pickering AJ, et al. Effects of water quality, sanitation, handwashing, and nutritional interventions on diarrhoea and child growth in rural Kenya: a cluster-randomised controlled trial. *Lancet Glob Health* 2018;6(3):e316-e29.
10. Pickering AJ, Djebbari H, Lopez C, et al. Effect of a community-led sanitation intervention on child diarrhoea and child growth in rural Mali: a cluster-randomised controlled trial. *Lancet Glob Health* 2015;3(11):e701-11.
11. Brown R, Leder, K., Wong, T. et al. Improving human and environmental health in urban informal settlements: the Revitalising Informal Settlements and their Environments (RISE) programme *Lancet Planetary Health* 2018;2:S29.
12. Pickering AJ, Null C, Winch PJ, et al. The WASH Benefits and SHINE trials: interpretation of WASH intervention effects on linear growth and diarrhoea. *Lancet Glob Health* 2019;7(8):e1139-e46.
13. Cumming O, Arnold BF, Ban R, et al. The implications of three major new trials for the effect of water, sanitation and hygiene on childhood diarrhea and stunting: a consensus statement. *BMC Med* 2019;17(1):173.
14. Whitmee S, Haines A, Beyrer C, et al. Safeguarding human health in the Anthropocene epoch: report of The Rockefeller Foundation-Lancet Commission on planetary health. *Lancet* 2015;386(10007):1973-2028.
15. Chown SL, Duffy GA. Thermal physiology and urbanization: perspectives on exit, entry and transformation rules. *Functional Ecology* 2015;29(7):902-12.
16. Seto KC, Sánchez-Rodríguez R, Fragkias M. The New Geography of Contemporary Urbanization and the Environment. *Annual Review of Environment and Resources* 2010;35(1):167-94.
17. Elmqvist T, Fragkias M, Goodness J, et al. Urbanization, Biodiversity and Ecosystem Services: Challenges and Opportunities. Dordrecht, Germany: Springer 2013.
18. Seto KC, Güneralp B, Hutyra LR. Global forecasts of urban expansion to 2030 and direct impacts on biodiversity and carbon pools. *PNAS* 2012;109(40):16083-88.
19. Oke TR. The energetic basis of the urban heat island. *Quarterly Journal of the Royal Meteorological Society* 1982;108(455):1-24.
20. Peng S, Piao S, Ciais P, et al. Surface Urban Heat Island Across 419 Global Big Cities. *Environ Sci Technol* 2012;46(2):696-703.
21. Katukiza AY, Ronteltap M, Niwagaba CB, et al. Sustainable sanitation technology options for urban slums. *Biotechnology Advances* 2012;30(5):964-78.
22. Alirol E, Getaz L, Stoll B, et al. Urbanisation and infectious diseases in a globalised world. *Lancet Infect Dis* 2011;11(2):131-41.




For peer review only - http://bmjopen.bmj.com/site/about/guidelines.xhtml

23. Eisenstein M. Disease: Poverty and pathogens. *Nature* 2016;531(7594):S61-63.
24. Himsworth CG, Parsons KL, Jardine C, et al. Rats, cities, people, and pathogens: a systematic review and narrative synthesis of literature regarding the ecology of rat-associated zoonoses in urban centers. *Vector Borne Zoonotic Dis* 2013;13(6):349-59.
25. Franklinos LHV, Jones KE, Redding DW, et al. The effect of global change on mosquito-borne disease. *The Lancet Infectious Diseases* 2019;19(9):e302-e12.
26. Hassell JM, Begon M, Ward MJ, et al. Urbanization and Disease Emergence: Dynamics at the Wildlife–Livestock–Human Interface. *Trends in Ecology & Evolution* 2017;32(1):55-67.
27. Lau CL, Smythe LD, Craig SB, et al. Climate change, flooding, urbanisation and leptospirosis: fuelling the fire? *Trans R Soc Trop Med Hyg* 2010;104(10):631-38.
28. Carvalho S, Magalhães MdAFM, Medronho RdA. Analysis of the spatial distribution of dengue cases in the city of Rio de Janeiro, 2011 and 2012. *Rev Saude Publica* 2017;51:79.
29. Daudé É, Mazumdar S, Solanki V. Widespread fear of dengue transmission but poor practices of dengue prevention: A study in the slums of Delhi, India. *PLoS Med* 2017;12(2):e0171543.
30. Feleke H, Medhin G, Abebe A, et al. Enteric pathogens and associated risk factors among under-five children with and without diarrhea in Wegera District, Northwestern Ethiopia. *Pan Afr Med J* 2018;29:72.
31. Korpe PS, Valencia C, Haque R, et al. Epidemiology and Risk Factors for Cryptosporidiosis in Children From 8 Low-income Sites: Results From the MAL-ED Study. *Clin Infect Dis* 2018;67(11):1660-69.
32. Hagan JE, Moraga P, Costa F, et al. Spatiotemporal Determinants of Urban Leptospirosis Transmission: Four-Year Prospective Cohort Study of Slum Residents in Brazil. *PLoS Negl Trop Dis* 2016;10(1):e0004275.
33. Majorin F, Torondel B, Routray P, et al. Identifying Potential Sources of Exposure Along the Child Feces Management Pathway: A Cross-Sectional Study Among Urban Slums in Odisha, India. *The American Journal of Tropical Medicine and Hygiene* 2017;97(3):861-69.
34. World Health Organisation. Global antimicrobial resistance surveillance system (GLASS) report: Early implementation 2016-2017. Accessed from https://www.who.int/glass/resources/publications/early-implementation-report/en/, June 10th 2020.
35. Pehrsson EC, Tsukayama P, Patel S, et al. Interconnected microbiomes and resistomes in low-income human habitats. *Nature* 2016;533:212-16.
36. Nadimpalli ML, Marks SJ, Montealegre MC, et al. Urban informal settlements as hotspots of antimicrobial resistance and the need to curb environmental transmission. *Nat Microbiol* 2020;5(6):787-95.
37. Keusch GT, Rosenberg IH, Denno DM, et al. Implications of acquired environmental enteric dysfunction for growth and stunting in infants and children living in low- and middle-income countries. *Food Nutr Bull* 2013;34(3):357-64.
38. Naylor C, Lu M, Haque R, et al. Environmental Enteropathy, Oral Vaccine Failure and Growth Faltering in Infants in Bangladesh. *EBioMedicine* 2015;2(11):1759-66.
39. Vlahov D, Freudenberg N, Proietti F, et al. Urban as a Determinant of Health. *J Urban Health* 2007;84(Suppl 1):16-26.
40. Ferguson KT, Cassells RC, MacAllister JW, et al. The physical environment and child development: An international review. *International Journal of Psychology* 2013;48(4):437-68.
41. Guite HF, Clark C, Ackrill G. The impact of the physical and urban environment on mental well-being. *Public Health* 2006;120(12):1117-26.
42. Devoto F, Duflo E, Dupas P, et al. Happiness on Tap: Piped Water Adoption in Urban Morocco. *American Economic Journal: Economic Policy* 2012;4(4):68-99.
43. Evans GW. Child Development and the Physical Environment. *Annual Review of Psychology* 2006;57(1):423-51.
44. Lambert A, Vlaar J, Herrington S, et al. What Is the Relationship between the Neighbourhood Built Environment and Time Spent in Outdoor Play? A Systematic Review. *International Journal of Environmental Research and Public Health* 2019;16(20):3840.
45. Marx B, Stoker T, Suri T. The Economics of Slums in the Developing World. *Journal of Economic Perspectives* 2013;17(4):187-210.



For peer review only - http://bmjopen.bmj.com/site/about/guidelines.xhtml

46. Pieter van Dijk M, Etajak S, Mwalwega B, et al. Financing sanitation and cost recovery in the slums of Dar es Salaam and Kampala. *Habitat International* 2014;43:206-13.
47. Moreno EL. Slums of the world: The face of urban poverty in the new millennium. Nairobi, Kenya: UN-HABITAT, 2003.
48. Kadlac RH, Wallace SD. Treatment Wetlands, 2nd Ed. Florida, USA: CRC Press 2008.
49. Wu S, Carvalho PN, Müller JA, et al. Sanitation in constructed wetlands: A review on the removal of human pathogens and fecal indicators. *Science of The Total Environment* 2016;541:8-22.
50. Zhao Z, Song X, Wang Y, et al. Effects of algal ponds on vertical flow constructed wetlands under different sewage application techniques. *Ecological Engineering* 2016;93:120-28.
51. Bratieres K, Fletcher TD, Deletic A, et al. Nutrient and sediment removal by stormwater biofilters: A large-scale design optimisation study. *Water Research* 2008;42(14):3930-40.
52. Hatt BE, Fletcher TD, Deletic A. Hydraulic and Pollutant Removal Performance of Fine Media Stormwater Filtration Systems. *Environ Sci Technol* 2008;42(7):2535-41.
53. Kim H, Seagren EA, Davis AP. Engineered bioretention for removal of nitrate from stormwater runoff. *Water Environ Res* 2003;75(4):355-67.
54. Read J, Wevill T, Fletcher T, et al. Variation among plant species in pollutant removal from stormwater in biofiltration systems. *Water Research* 2008;42(4):893-902.
55. Davies CM, Bavor HJ. The fate of stormwater-associated bacteria in constructed wetland and water pollution control pond systems. *J Appl Microbiol* 2000;89(2):349-60.
56. García JA, Paredes D, Cubillos JA. Effect of plants and the combination of wetland treatment type systems on pathogen removal in tropical climate conditions. *Ecological Engineering* 2013;58:57-62.
57. Walker DJ, Hurl S. The reduction of heavy metals in a stormwater wetland. *Ecological Engineering* 2002;18(4):407-14.
58. Wong T, Breen PF, Somes NLG, et al. Managing Urban Stormwater using Constructed Wetlands, Industry Report 98/7. Melbourne, Australia: Cooperative Research Centre for Catchment Hydrology, 1998.
59. Austrade. China's Sponge City Program: Making China's cities watertight presents opportunities for Australian water technologies, 2016. Accessed from https://goo.gl/kHUcqh, May 10th 2020.
60. Cooperative Research Centre for Water Sensitive Cities. Kunshan awarded status as a National Ecological Garden City, 2016. Accessed from https://watersensitivecities.org.au/content/kunshan-awarded-status-national-ecological-garden-city/, May 8th 2020.
61. Jewish National Fund. New Bio-filters Purify Surface Runoff Water for Reuse, 2015. Accessed from http://www.kkl-jnf.org/about-kkl-jnf/green-israel-news/march-2015/biofilters-ramle-bat-yam, May 8th 2020.
62. O'Meara S. Why China Wants to Build Something Called Sponge Cities, CityLab, 2015. Accessed from https://www.citylab.com/design/2015/11/why-china-wants-to-build-sponge-cities/417114/, May 6th 2020.
63. Monitoring Singapore's first bioretention system: Rain garden at Balam Estate. In: Proceedings of Water Sensitive Urban Design; Building the Water Sensiitve Community, Melbourne, Australia, 2012.
64. Active, Beautiful and Clean (ABC) Waters Programme: towards sustainable stormwater management in Singapore. In: Proceedings of the 6th International Water Sensitive Urban Design Conference and Hydropolis, Perth, Australia, 2009.
65. Framework for Stormwater Quality Management in Singapore. Proceedings of the 12th International Conference on Urban Drainage, Porto Algere, Brazil, 2011.
66. A Dual-mode Biofilter System: Case study in Kfar Sava, Israel. Proceedings of the 12th International Conference on Urban Drainage, Porto Alegre, Brazil, 2011.
67. Liu J, Gratz J, Amour C, et al. A laboratory-developed TaqMan Array Card for simultaneous detection of 19 enteropathogens. *J Clin Microbiol* 2013;51(2):472-80.
68. Liu J, Platts-Mills JA, Juma J, et al. Use of quantitative molecular diagnostic methods to identify causes of diarrhoea in children: a reanalysis of the GEMS case-control study. *Lancet* 2016;388(10051):1291-301.
69. Wagner M, Peterson CGB, Ridefelt P, et al. Fecal markers of inflammation used as surrogate markers for treatment outcome in relapsing inflammatory bowel disease. *World J Gastroenterol* 2008;14(36):5584-89.



For peer review only - http://bmjopen.bmj.com/site/about/guidelines.xhtml

70. Bernier JJ, Florent C, Desmazures C, et al. Diagnosis of protein-losing enteropathy by gastrointestinal clearance of alpha1-antitrypsin. *Lancet* 1978;2(8093):763-64.
71. Campbell DI, McPhail G, Lunn PG, et al. Intestinal inflammation measured by fecal neopterin in Gambian children with enteropathy: association with growth failure, Giardia lamblia, and intestinal permeability. *J Pediatr Gastroenterol Nutr* 2004;39(2):153-57.
72. Overbey KN, Schwab KJ, Exum NG. Comparison of 1-week and 2-week recall periods for caregiver-reported diarrhoeal illness in children, using nationally representative household surveys. *Int J Epidemiol* 2019;48(4):1228-39.
73. de Onis M, Onyango AW, Van den Broeck J, et al. Measurement and standardization protocols for anthropometry used in the construction of a new international growth reference. *Food Nutr Bull* 2004;25(1 Suppl):S27-36.
74. Ayele B, Aemere A, Gebre T, et al. Reliability of measurements performed by community-drawn anthropometrists from rural Ethiopia. *PLOS ONE* 2012;7(1):e30345.
75. Varni JW, Seid M, Kurtin PS. PedsQL 4.0: reliability and validity of the Pediatric Quality of Life Inventory version 4.0 generic core scales in healthy and patient populations. *Med Care* 2001;39(8):800-12.
76. Krueger AB, Stone AA. Progress in measuring subjective well-being. *Science* 2014;436(42)
77. Organisation for Economic Co-operation and Development. OECD Guidelines on Measuring Subjective Wellbeing. Paris, France: OECD Publishing, 2013.
78. Grootaert C, Narayan D, Woolcock M, et al. Measuring social capital: an integrated questionnaire. Washington DC: World Bank, 2004.
79. Henry R, Schang C, Chandrasena GI, et al. Environmental monitoring of waterborne Campylobacter: evaluation of the Australian standard and a hybrid extraction-free MPN-PCR method. *Front Microbiol* 2015;6:74.
80. Jones NR, Millman C, van der Es M, et al. Novel Sampling Method for Assessing Human-Pathogen Interactions in the Natural Environment Using Boot Socks and Citizen Scientists, with Application to Campylobacter Seasonality. *Appl Environ Microbiol* 2017;83(14):e00162-17.
81. Schang C, Henry R, Kolotelo PA, et al. Evaluation of Techniques for Measuring Microbial Hazards in Bathing Waters: A Comparative Study. *PLOS ONE* 2016;11(5):e0155848.
82. Caporaso JG, Lauber CL, Walters WA, et al. Ultra-high-throughput microbial community analysis on the Illumina HiSeq and MiSeq platforms. *ISME J* 2012;6(8):1621-24.
83. Caporaso JG, Lauber CL, Walters WA, et al. Global patterns of 16S rRNA diversity at a depth of millions of sequences per sample. *Proc Natl Acad Sci USA* 2011;108 Suppl 1:4516-22.
84. Henry R, Schang C, Coutts S, et al. Into the deep: Evaluation of SourceTracker for assessment of faecal contamination of coastal waters. *Water Research* 2016;93:242-53.
85. Knights D, Kuczynski J, Charlson ES, et al. Bayesian community-wide culture-independent microbial source tracking. *Nat Methods* 2011;8(9):761-3.
86. Fu P, Weng Q. A time series analysis of urbanization induced land use and land cover change and its impact on land surface temperature with Landsat imagery. *Remote Sensing of Environment* 2016;175:205-14.
87. Williams CR, Long SA, Webb CE, et al. Aedes aegypti Population Sampling Using BG-Sentinel Traps in North Queensland Australia: Statistical Considerations for Trap Deployment and Sampling Strategy. *J Med Entomol* 2007;44(2):345-50.
88. Huang YM. The mosquitoes of Polynesia with a pictorial key to some species associated with filariasis and/or dengue fever. *Mosquito Systematics* 1977;9(3):289-322.
89. Reuben R, Tewari SC, Hiriyan J, et al. Illustrated keys to species of Culex (Culex) associated with Japanese encephalitis in Southeast Asia (Diptera: Culicidae). *Mosquito systematics* 1994;26(2):75-96.
90. Rueda L. Pictorial keys for the identification of mosquitoes (Diptera: Culicidae) associated with Dengue Virus Transmission. *Zootaxa* 2004;589.
91. Aplin KP, Brown PR, Jacob J, et al. Field methods for rodent studies in Asia and the Pacific. Canberra, Australia: Australian Centre for International Agricultural Research, 2003.
92. Himsworth CG, Jardine CM, Parsons KL, et al. The Characteristics of Wild Rat (Rattus spp.) Populations from an Inner-City Neighborhood with a Focus on Factors Critical to the Understanding of Rat-Associated Zoonoses. *PLOS ONE* 2014;9(3)



For peer review only - http://bmjopen.bmj.com/site/about/guidelines.xhtml

93. Mordecai EA, Caldwell JM, Grossman MK, et al. Thermal biology of mosquito-borne disease. *Ecology Letters* 2019;22(10):1690-708.
94. Bradfer-Lawrence T, Gardner N, Bunnefeld L, et al. Guidelines for the use of acoustic indices in environmental research. *Methods in Ecology and Evolution* 2019;10(10):1796-807.
95. Hooper R, Teerenstra S, de Hoop E, et al. Sample size calculation for stepped wedge and other longitudinal cluster randomised trials. *Stat Med* 2016;35(26):4718-28.
96. Clasen TF, Alexander KT, Sinclair D, et al. Interventions to improve water quality for preventing diarrhoea. *Cochrane Database Syst Rev* 2015(10):CD004794.
97. Ziegelbauer K, Speich B, Mausezahl D, et al. Effect of sanitation on soil-transmitted helminth infection: systematic review and meta-analysis. *PLoS Med* 2012;9(1):e1001162.
98. Mancl LA, DeRouen TA. A covariance estimator for GEE with improved small-sample properties. *Biometrics* 2001;57(1):126-34.
99. Schmidt WP. The elusive effect of water and sanitation on the global burden of disease. *Trop Med Int Health* 2014;19(5):522-7.
100. Luby SP, Agboatwalla M, Hoekstra RM. The variability of childhood diarrhea in Karachi, Pakistan, 2002-2006. *The American Journal of Tropical Medicine and Hygiene* 2011;84(6):870-77.
101. Taniuchi M, Sobuz SU, Begum S, et al. Etiology of diarrhea in Bangladeshi infants in the first year of life analyzed using molecular methods. *J Infect Dis* 2013;208(11):1794-802.
102. Kau AL, Planer JD, Liu J, et al. Functional characterization of IgA-targeted bacterial taxa from undernourished Malawian children that produce diet-dependent enteropathy. *Sci Transl Med* 2015;7(276):276ra24.
103. Smith MI, Yatsunenko T, Manary MJ, et al. Gut microbiomes of Malawian twin pairs discordant for kwashiorkor. *Science* 2013;339(6119):548-54.
104. Corburn J, Sverdlik A. Slum Upgrading and Health Equity. *Int J Environ Res Public Health* 2017;14(4)
105. Field E. KM. Impact Evaluation for Slum Upgrading Interventions. Harvard University: Cambridge, MA, USA, 2006. Accessed from https://scholar.harvard.edu/files/field/files/kremer_field.pdf, 17th October 2020.
106. Jaitman L, Brakarz, J. Evaluation of slum upgrading programs: Literature review and methodological approaches. Technical note No. IDB-TN-604, 2010. Accessed from https://publications.iadb.org/publications/english/document/Evaluation-of-Slum-Upgrading-Programs-Literature-Review-and-Methodological-Approaches.pdf, 17th October 2020.
107. Butala NM, VanRooyen MJ, Patel RB. Improved health outcomes in urban slums through infrastructure upgrading. *Soc Sci Med* 2010;71(5):935-40.

For peer review only



For peer review only - http://bmjopen.bmj.com/site/about/guidelines.xhtml

**Authors' contributions**

Devised the project's main conceptual ideas: KL, PA, TC, SLC, ABF, MF, DJ, DRL, SPL, DM, DR, JAS, TW, RB
Contributed to development of protocol details and implementation: KL, JO, AA, SFB, KB, TC, SLC, GAD, PAF, GF, MF, CG, RH, EH, DJ, RL, AL, SPL, DM, JO'T, DRL, SSS, RS, RT, AT, ART, JW, TW, RB
Designed the statistical analysis plan: ABF and JAS
Main drafting of the main manuscript: KL, JO
Critical review and approval of the manuscript: All authors

**Funding:** This trial has been primarily funded by the Wellcome Trust "Our Planet, Our Health" grant [205222/Z/16/Z]. The intervention is funded by the Asian Development Bank, the New Zealand Ministry of Foreign Affairs and Trade, and Monash University. The funders have no role in the study design, data collection and analysis, decision to publish, or preparation of manuscripts.

**Competing interests:** None

**Acknowledgements:** We acknowledge the hard work and dedication of the RISE project field teams in Fiji and Indonesia, and all the study participants. We also thank Dasha Spasjevic for providing Figure 3.

For peer review only



For peer review only - http://bmjopen.bmj.com/site/about/guidelines.xhtml

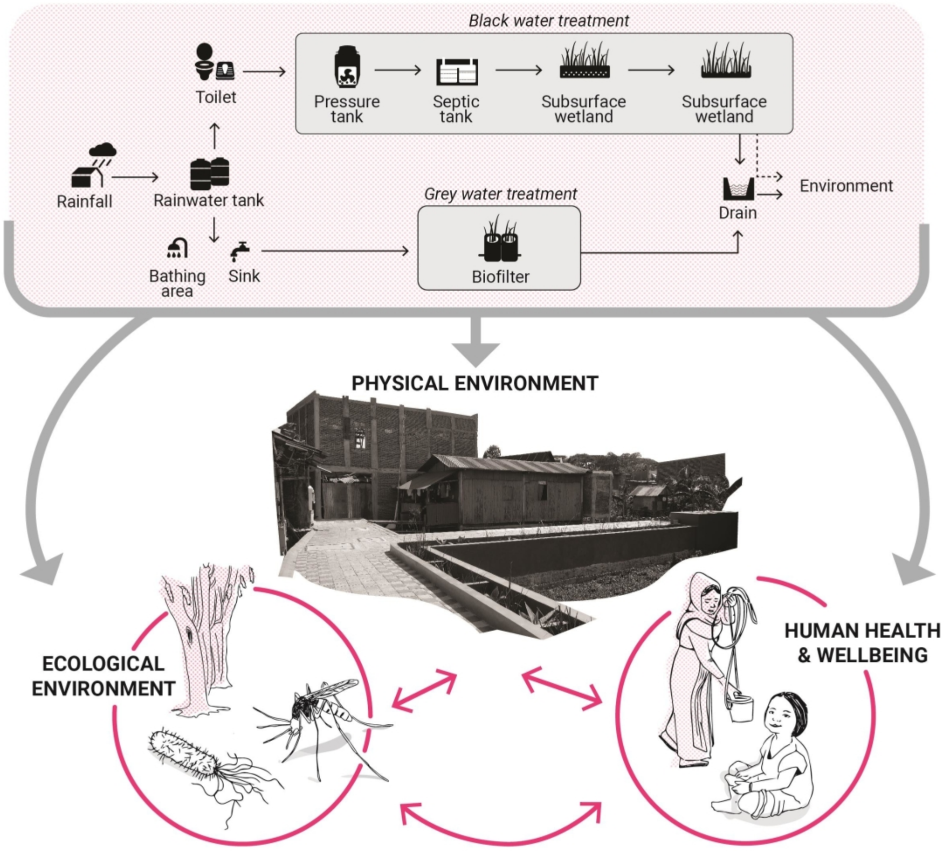


Figure 1: The RISE study hypothesised theory of impacts. The water sensitive intervention will improve the physical environmental conditions, resulting in both direct and indirect impacts on ecology and human physical health and wellbeing

209x191mm (300 x 300 DPI)

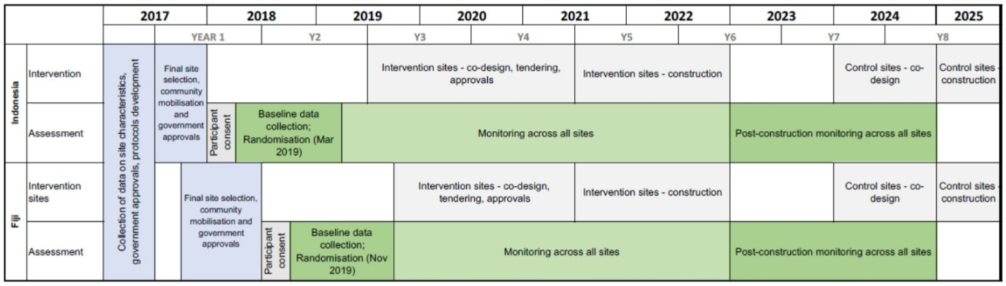


Figure 2: Anticipated RISE study timeline

146x41mm (300 x 300 DPI)

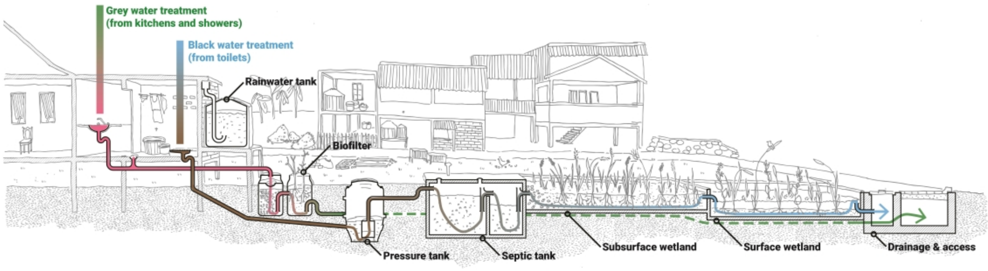


Figure 3: Schematics of proposed sanitation intervention

703x202mm (300 x 300 DPI)

**Checkllist SPIRIT -complete**

| Item no | Description | Information | Found on what Page of the protocol? - current Page numbers |
|---|---|---|---|
| 1 | Descriptive title identifying the study design, population, interventions, and, if applicable, trial acronym | All information in current title: "Study design, rationale and assessment methods of the Revitalising Informal Settlements and their Environments (RISE) study: a cluster randomised controlled trial to evaluate a human health impacts of a water sensitive intervention in informal settlements in Indonesia and Fiji" | Page 1 |
| 2a | Trial identifier and registry name. If not yet registered, name of intended registry | Registration: Australian New Zealand Clinical Trials Registry (ANZCTR) (Trial ID: ACTRN12618000633280). | Abstract (Page 2) and 'Ethics and Dissemination' section (Page 16) |
| 2b | All items from the World Health Organization Trial Registration Data Set | See WHO Trial Registration dataset collection | See WHO Trial Registration dataset collection |
| 3 | Date and version identifier | Article submission will be of version 1 with date of submission identifier | Version 2; 02/11/2020 |
| 4 | Sources and types of financial, material, and other support | See text prepared for end of article: Funding: This trial has been primarily funded by the Wellcome Trust "Our Planet, Our Health" grant 205222/Z/16/Z | See Funding (Page 22) |
| 5a | Names, affiliations, and roles of protocol contributors | | Pages 1 & 22 |
| 5b | Name and contact information for the trial sponsor | Professor Rebekah Brown Senior Vice-Provost & Vice-Provost (Research) and Program Director RISE Revitalising Informal Settlements and their Environments (RISE) Monash University Office of the Provost and Senior Vice-President Chancellery 27 Chancellors Walk Clayton VIC 3800 Australia T: +61 3 9905 2122 E: rebekah.brown@monash.edu | See WHO Trial Registration data set |
| 5c | Role of study sponsor and funders, if any, in study design; collection, management, analysis, and interpretation of data; writing of the report; and the decision to submit the report for publication, including whether they will have ultimate authority over any of these activities | The funders have no role in study design, data collection and analysis, decision to publish, or preparation of manuscripts.' | See 4 above (Page 22) |
| 5d | Composition, roles, and responsibilities of the coordinating centre, steering committee, endpoint adjudication committee, data management team, and other individuals or groups overseeing the trial, if applicable (see Item 21a for data monitoring committee) | The RISE study has an independent International Scientific Advisory Panel (ISAP) and a Governance Committee, both of which provide oversight and advice including in relation to any unintended effects of the trial intervention.<br><br>Specifically, ISAP will provide strategic support and scientific guidance to ensure the highest levels of scientific rigour are adopted and followed. This will ensure that results can stand up to international scrutiny and inform policy and investments. ISAP also provides strategic independent advice on the development and implementation of the | See Page 14 |

Version 2: 2/11/2020

| | | | |
|---|---|---|---|
| | | RISE program, and, where relevant, informs the RISE team of state-of-the-art research methodologies, tools, and best practices. ISAP members will also serve as a DSMB, and will specifically advise on responses to ethical issues or harms if any are detected in the intervention groups. After significant discussion, and given the complex nature of the trial, using a Panel of experts already familiar with the project to serve in this role was deemed a more appropriate and expedient approach than having another separate (traditional DSMB) committee.<br><br>The Governance Advisory Panel serves as an advisory body to the Executive Team, specifically providing independent strategic risk and program management advice.<br><br>The study also has a Data Advisory Panel, comprised of representatives across Monash University who provide strategic advice and operational guidance to assure a holistically-advised research data strategy. Specifically, this panel provides advice on how data are collected, stored and shared. The Panel will strengthen the strategy through promotion of best practices and utilisation of fit-for-purpose research infrastructure that in-turn ensure research data accelerates research activity, whilst also maintaining data protection and privacy in accordance with ethical and regulatory requirements.<br><br>Also see Study oversight section | |
| 6a | Description of research question and justification for undertaking the trial, including summary of relevant studies (published and unpublished) examining benefits and harms for each intervention | This information is included in Introduction, Hypotheses, and Aims | Pages 4, 5, 6 |
| 6b | Explanation for choice of comparators | Yes – clearly explained in Study Methods and Analysis: "RISE is a parallel-cluster RCT involving 12 informal settlements in Suva, Fiji, and 12 in Makassar, Indonesia. In each country, half of the sites have been randomised to receive an early upgrade ('intervention' communities), and the remaining will serve as controls". | Page 6 |
| 7 | Specific objectives or hypotheses | See Hypotheses, and Aims | Pages 4, 5, 6 |
| 8 | Description of trial design including type of trial (eg, parallel group, crossover, factorial, single group), allocation ratio, and framework (eg, superiority, equivalence, noninferiority, exploratory) | Parallel design in title and throughout (superiority trial framework)<br>Statistical analyses section | Page 14 |
| 9 | Description of study settings (eg, community clinic, academic hospital) and list of countries where data will be collected. Reference to where list of study sites can be obtained | See Study settings | Pages 6,7 |



For peer review only - http://bmjopen.bmj.com/site/about/guidelines.xhtml

| | | | |
|---|---|---|---|
| 10 | Inclusion and exclusion criteria for participants. If applicable, eligibility criteria for study centres and individuals who will perform the interventions (eg, surgeons, psychotherapists) | Inclusion and exclusion criteria given in section 'Settlement eligibility and selection criteria" and provided in Table 1 | Pages 7,8 |
| 11a | Interventions for each group with sufficient detail to allow replication, including how and when they will be administered | The intervention is fully described in Section 'Description of the intervention' – including codesign which allows for some variation between sites | Pages 8,9 |
| 11b | Criteria for discontinuing or modifying allocated interventions for a given trial participant (eg, drug dose change in response to harms, participant request, or improving/worsening disease) | Not applicable as the intervention is being implemented at the community level | NA |
| 11c | Strategies to improve adherence to intervention protocols, and any procedures for monitoring adherence (eg, drug tablet return, laboratory tests) | Long-term operation and maintenance responsibilities will be addressed via training and ongoing engagement of community representatives and local authorities. | Page 9 |
| 11d | Relevant concomitant care and interventions that are permitted or prohibited during the trial | Standard hygiene messages will be provided to households in both early- and delayed-intervention communities | Page 6 |
| 12 | Primary, secondary, and other outcomes, including the specific measurement variable (eg, systolic blood pressure), analysis metric (eg, change from baseline, final value, time to event), method of aggregation (eg, median, proportion), and time point for each outcome. Explanation of the clinical relevance of chosen efficacy and harm outcomes is strongly recommended | All primary and secondary outcomes are given under the Outcomes heading on Page 8 and onwards. The specific measurement variables are stated in Table 2<br>We state that presence, abundance and diversity will be compared. We detail the time point for each outcome.<br>We describe median values for height / weight measurements but otherwise method of aggregation in outcomes section; otherwise details in sample size section.<br>Action levels (harm) for Hb and Z score plus helminth given in 'Referral guidelines' Page 13/14 | Table 2 Pages 9- 10<br>Outcomes section, Pages 11, 12<br>Referral guidelines Pages 12,13<br>Sample size Page 13 |
| 13 | Time schedule of enrolment, interventions (including any run-ins and washouts), assessments, and visits for participants. A schematic diagram is highly recommended (see Figure) | Schematic diagram is provided | See Fig 2 |
| 14 | Estimated number of participants needed to achieve study objectives and how it was determined, including clinical and statistical assumptions supporting any sample size calculations | See sample size section | Page 13 |
| 15 | Strategies for achieving adequate participant enrolment to reach target sample size | Community cooperation was obtained prior to enrolment of HH– see section Site and HH recruitment Page 8. | Page 8 |
| 16a | Method of generating the allocation sequence (eg, computer-generated random numbers), and list of any factors for stratification. To reduce predictability of a random sequence, details of any planned restriction (eg, blocking) should be provided in a separate document that is | See Section 'Randomisation' | Page 13 |

Version 2: 2/11/2020

For peer review only

| | | | |
|---|---|---|---|
| | unavailable to those who enrol participants or assign interventions | | |
| 16b | Mechanism of implementing the allocation sequence (eg, central telephone; sequentially numbered, opaque, sealed envelopes), describing any steps to conceal the sequence until interventions are assigned | See 'Randomisation' section | Page 13 |
| 16c | Who will generate the allocation sequence, who will enrol participants, and who will assign participants to interventions | Statisticians generated a series of allocations of settlements to intervention and control groups satisfying the balance criteria, from which one allocation was randomly chosen by a child from the pilot community in each country (using the tombola method, see details in 'Randomisation section).<br>'Household recruitment was performed by trained local community field worker teams fluent in local languages.' | Page 13<br><br>Page 8 |
| 17a | Who will be blinded after assignment to interventions (eg, trial participants, care providers, outcome assessors, data analysts), and how | Unique, numeric identifiers have been generated for houses, households and participants.<br><br>Samples: Samples collected by field staff are identified with unique barcodes. Barcodes for human samples do not identify the settlement. Therefore, laboratory staff are blinded to the identity of sample collection location. After sample processing, all samples receive a second unique non-identifiable barcode prior to long-term storage at -80C.<br><br>Data: Information about participants (name, date of birth, etc.) is needed by surveyors to conduct household surveys. Paper copies of personal information used by the surveyors is stored in a locked cabinet and will be destroyed when no longer required. Prior to analysis of household survey data, personal details including names, phone numbers and dates of birth is removed, with data instead linked using the unique identifiers. | Page 14 |
| 17b | If blinded, circumstances under which unblinding is permissible, and procedure for revealing a participant's allocated intervention during the trial | Not applicable: no blinding of participants. It is impossible to blind community participants given this is a physical intervention. | Not applicable |
| 18a | Plans for assessment and collection of outcome, baseline, and other trial data, including any related processes to promote data quality (eg, duplicate measurements, training of assessors) and a description of study instruments (eg, questionnaires, laboratory tests) along with their reliability and validity, if known. Reference to where data collection forms can be found, if not in the protocol | Human anthropometrics will be collected as triplicate measurements –<br>Local trained community field are employed<br>Validated Questionnaires to be used | Page 11<br><br>Page 8<br>Page 11 |
| 18b | Plans to promote participant retention and complete follow-up, | To maintain participation over the trial period small culturally appropriate gifts of | Page 9 |

Version 2: 2/11/2020

| | | | |
|---|---|---|---|
| | including list of any outcome data to be collected for participants who discontinue or deviate from intervention protocols | nominal value – as recommended by our in-country partners – will be provided. | |
| 19 | Plans for data entry, coding, security, and storage, including any related processes to promote data quality (eg, double data entry; range checks for data values). Reference to where details of data management procedures can be found, if not in the protocol | Details available in Ethics application (available on request) | Details available in Ethics application (available on request) |
| 20a | Statistical methods for analysing primary and secondary outcomes. Reference to where other details of the statistical analysis plan can be found, if not in the protocol | See statistical section | Page 14 |
| 20b | Methods for any additional analyses (eg, subgroup and adjusted analyses) | See statistical section | Page 14 |
| 20c | Definition of analysis population relating to protocol non-adherence (eg, as randomised analysis), and any statistical methods to handle missing data (eg, multiple imputation) | Will be analysed as intention to treat | Page 14 |
| 21a | Composition of data monitoring committee (DMC); summary of its role and reporting structure; statement of whether it is independent from the sponsor and competing interests; and reference to where further details about its charter can be found, if not in the protocol. Alternatively, an explanation of why a DMC is not needed | See study oversight section and section #5d above. As noted, members of the International Scientific Advisory Panel will also serve as a DSMB, and will specifically advise on responses to ethical issues or harms if any are detected in the intervention groups. After significant discussion, and given the complex nature of the trial, using a Panel of experts already familiar with the project to serve in this role was deemed a more appropriate and expedient approach than having another separate (traditional DSMB) committee. | Page 14 |
| 21b | Description of any interim analyses and stopping guidelines, including who will have access to these interim results and make the final decision to terminate the trial | See Study Oversight section.<br>Given the nature of this trial, there are no stopping rules. Results of the intervention implementation in the first 12 settlements will be used to inform and optimise rollout in the control settlements following completion of the trial. | Page 14 |
| 22 | Plans for collecting, assessing, reporting, and managing solicited and spontaneously reported adverse events and other unintended effects of trial interventions or trial conduct | See study oversight section<br>"The RISE study has an independent International Scientific Advisory Panel and a Governance Committee, both of which provide oversight and advice, including in relation to any unintended effects of the trial intervention or trial conduct. | Page 14 |
| 23 | Frequency and procedures for auditing trial conduct, if any, and whether the process will be independent from investigators and the sponsor | See study oversight section | Page 14 |
| 24 | Plans for seeking research ethics committee/institutional review board (REC/IRB) approval | See 'Ethics and dissemination section' | Page 16 |
| 25 | Plans for communicating important protocol modifications (eg, changes to eligibility criteria, outcomes, | Trial registries, investigators and human research ethics committees are notified of changes, as relevant. | NA |

Version 2: 2/11/2020

For peer review only

| | | | |
|---|---|---|---|
| | analyses) to relevant parties (eg, investigators, REC/IRBs, trial participants, trial registries, journals, regulators) | | |
| 26a | Who will obtain informed consent or assent from potential trial participants or authorised surrogates, and how (see Item 32) | See Site and HH recruitment | Page 8 |
| 26b | Additional consent provisions for collection and use of participant data and biological specimens in ancillary studies, if applicable | Documented in Consent and participation forms | Available on request |
| 27 | How personal information about potential and enrolled participants will be collected, shared, and maintained in order to protect confidentiality before, during, and after the trial | Documented in Consent and participation forms.<br><br>Surveyors (community field workers) are independently employed as project staff, and none are from our study sites or from neighbouring settlements. They are all required to sign confidentiality agreements and undergo extensive training in objective survey and sample collection. This training is repeated before each household survey round.<br><br>No identifiable data will be published. | Available on request |
| 28 | Financial and other competing interests for principal investigators for the overall trial and each study site | See competing interests = none | Page 22 |
| 29 | Statement of who will have access to the final trial dataset, and disclosure of contractual agreements that limit such access for investigators | See Dissemination | Page 16 |
| 30 | Provisions, if any, for ancillary and post-trial care, and for compensation to those who suffer harm from trial participation | Not applicable as the intervention is being implemented at the community level | NA |
| 31a | Plans for investigators and sponsor to communicate trial results to participants, healthcare professionals, the public, and other relevant groups (eg, via publication, reporting in results databases, or other data sharing arrangements), including any publication restrictions | The data collected in the study will be publicly distributed along with metadata and critical documents (i.e, protocols and questionnaires) following the publication of the primary results from the trials.<br><br>We are engaging with local communities, participants and other stakeholders throughout the project in a number of ways).<br>• Participant feedback:<br>- Community feedback on preliminary findings is provided through tailored mechanisms, including (i) annual meetings of the Community Engagement Councils (e.g. presenting baseline findings at randomisation events), (ii) household information sheets; (iii) quarterly project newsletters explaining upcoming campaigns, changes to the program, and findings to date; and (iv) Community Engagement Committee meetings where our community field workers communicate the status of | In Ethics and Dissemination Section (Page 16) |

Version 2: 2/11/2020

For peer review only - http://bmjopen.bmj.com/site/about/guidelines.xhtml

| | | | |
|---|---|---|---|
| | | research and any significant findings since the last meeting.<br>- Health findings: abnormal Kato Katz, haemoglobin and anthropometric results are provided in written form to households, with support (including a referral letter and transportation costs) to seek care at local health facilities.<br>• Government stakeholders: Preliminary findings and results are presented at the Country-level Technical Coordination Committees, which meet 6-monthly and involve all local partners, relevant Ministries and local authorities. This incorporates feedback on sampling campaigns, response rates, and outcomes form project activities. Reports required for the Asian Development Bank (ADB), such as the ADB Knowledge Products, have also served as a vehicle for communicating preliminary outcomes from the intervention design process.<br>• Global - Feedback on emerging findings and research highlights through monthly newsletters, blog posts (on our website), and the usual additional mechanisms such as conference presentations, journal articles, etc. | |
| 31b | Authorship eligibility guidelines and any intended use of professional writers | See Author's contribution | Page 22 |
| 31c | Plans, if any, for granting public access to the full protocol, participant-level dataset, and statistical code | See Ethics and Dissemination Section | Page 16 |
| 32 | Model consent form and other related documentation given to participants and authorised surrogates | Documented in Consent and participation forms | Available on request |
| 33 | Plans for collection, laboratory evaluation, and storage of biological specimens for genetic or molecular analysis in the current trial and for future use in ancillary studies, if applicable | We will use various molecular approaches to assess the presence and concentration of pathogens, microbial communities, and AMR genes in human faecal samples p See Primary Outcomes<br>Documented in Consent and participation forms | Pages 11, 12<br><br><br><br><br>Available on request |

Version 2: 2/11/2020

For peer review only
